# STOPP/START criteria for potentially inappropriate prescribing in older people: version 3

**DOI:** 10.1007/s41999-023-00777-y

**Published:** 2023-05-31

**Authors:** Denis O’Mahony, Antonio Cherubini, Anna Renom Guiteras, Michael Denkinger, Jean-Baptiste Beuscart, Graziano Onder, Adalsteinn Gudmundsson, Alfonso J. Cruz-Jentoft, Wilma Knol, Gülistan Bahat, Nathalie van der Velde, Mirko Petrovic, Denis Curtin

**Affiliations:** 1 Department of Medicine (Geriatric Medicine), School of Medicine, University College Cork, Cork, Ireland; 2 Department of Geriatric Medicine, Cork University Hospital, Wilton, Cork, Ireland; 3 Geriatria, Accettazione Geriatrica e Centro di Ricerca Per l’invecchiamento, IRCCS INRCA, Ancona, Italy; 4 Department of Geriatric Medicine, Parc de Salut Mar, Barcelona, Spain; 5 Institute for Geriatric Research, University of Ulm Geriatric Center, Alb-Donau, Ulm, Germany; 6 Univ. Lille, CHU Lille, ULR 2694 - METRICS: Évaluation des Technologies de Santé et des Pratiques Médicales, 59000 Lille, France; 7 Fondazione Policlinico Gemelli IRCCS and Università Cattolica del Sacro Cuore, Rome, Italy; 8 Landspitali University Hospital, Reykjavik, Iceland; 9 Servicio de Geriatría, Hospital Universitario Ramón y Cajal (IRYCIS), Madrid, Spain; 10 Department of Geriatric Medicine and Expertise Centre Pharmacotherapy in Old Persons (EPHOR), University Medical Center Utrecht, Utrecht University, Utrecht, The Netherlands; 11 Division of Geriatrics, Department of Internal Medicine, Istanbul Medical School, Istanbul University, Istanbul, Turkey; 12 Amsterdam UMC Location University of Amsterdam, Internal Medicine, Section of Geriatric Medicine and Amsterdam Public Health Research Institute, Amsterdam, The Netherlands; 13 Department of Internal Medicine and Paediatrics, Faculty of Medicine and Health Sciences, University of Ghent, Ghent, Belgium

**Keywords:** STOPP/START criteria, Multimorbidity, Polypharmacy, Older people, Adverse drug events, Medication review

## Abstract

**Purpose:**

STOPP/START is a physiological systems-based explicit set of criteria that attempts to define the clinically important prescribing problems relating to potentially inappropriate medications (PIMs–STOPP criteria) and potential prescribing omissions (PPOs–START criteria). The previous two versions of STOPP/START criteria were published in 2008 and 2015. The present study describes the revised and updated third version of the criteria.

**Methods:**

A detailed system-by-system review of the published literature from April 2014 to March 2022 was undertaken with the aim of including clinically important new explicit PIM and PPO criteria and removing any criteria considered to be no longer correct or outdated. A panel of 11 academic physicians with recognized expertise in geriatric pharmacotherapy from 8 European countries participated in a Delphi panel with the task of validating the draft criteria. The panel was presented with the draft new criteria using the SurveyMonkey® on-line platform in which panelists were asked to indicate their level of agreement on a five-point Likert scale.

**Results:**

Two hundred and four evidence-based draft criteria (one hundred and forty-five STOPP criteria, fifty-nine START criteria) were presented to panelists for assessment using the Delphi validation method. Over the course of four rounds of Delphi validation, the panel achieved consensus on 133 STOPP criteria and 57 START criteria, i.e., 190 STOPP/START criteria in total representing a 66.7% increase in the number of criteria compared to STOPP/START version 2 published in 2015.

**Conclusion:**

A fully revised and updated version of STOPP/START criteria has been validated by a European expert panel using the Delphi consensus process.

**Supplementary Information:**

The online version contains supplementary material available at 10.1007/s41999-023-00777-y.

## Key summary points

**Aim:**

To update and validate STOPP/START criteria for potentially inappropriate prescribing.

**Findings:**

STOPP/START version 3 has been expanded and validated by an international European panel of experts in geriatric pharmacotherapy. Version 3, with 190 criteria, is significantly larger than version 2 (114 criteria), reflecting the expansion of the pharmacopeia and clinical trials evidence base relevant to older people since the publication of version 2.

**Message:**

STOPP/START version 3 represents an updated explicit list of potentially inappropriate medications and potential prescribing omissions aimed at optimizing medication and minimizing adverse drug reactions and events during medication review in older people, particularly those with multimorbidity and polypharmacy.

**Supplementary Information:**

The online version contains supplementary material available at 10.1007/s41999-023-00777-y.

## Background

In 2018, the Pharmaceutical Care Network of Europe defined medication review as “a structured evaluation of a patient’s medicines with the aim of optimizing medicines use and improving health outcomes [which] entails detecting drug-related problems and recommending interventions” [1]. Explicit criteria for potentially inappropriate prescribing (PIP) in older people have gained considerable attention and influence since the first publication of Beers criteria in 1991 [2]. Such criteria play an increasingly important part in both routine clinical practice and in research relating to PIP which should, in general, be avoided to minimize medication-related harm in older people. Detection and avoidance of PIMs is a fundamental aspect of routine medication review and is considered beneficial in older people with multimorbidity and associated polypharmacy undergoing comprehensive geriatric assessment.

Since the publication of the first and second versions of STOPP/START criteria in 2008 and 2015, respectively [3, 4], interest in the application of the criteria in routine practice has grown steadily. STOPP/START criteria are designed to help identify and support deprescribing of adverse medication and introduction of beneficial medication (where inappropriately unprescribed) as part of routine medication review in multimorbid older people with polypharmacy. STOPP criteria for detection of PIMs are based on physiological systems, as in most drug formularies, with additional categories relating to patients at risk of falls, patients taking opioid analgesics and patients taking drugs with anticholinergic properties. START criteria are designed to detect potential prescribing omissions (PPOs) which represent another critically important aspect of inappropriate prescribing, i.e., undertreatment or failure to prescribe appropriate medications despite clear and valid indications. In START criteria, PPOs are also arranged according to physiological systems for ease of use and include more important and common instances of potentially beneficial medication that may be inappropriately omitted. The first version of STOPP/START in 2008 included 65 STOPP and 22 START criteria [3]. STOPP/START version 2 in 2015 was expanded to include 80 STOPP PIM criteria and 34 START PPO criteria [4].

STOPP/START version 2 criteria have been approved by the UK National Institute for Clinical Excellence (NICE) [5] and by the Royal College of General Practitioners and British Geriatrics Society in the UK [6] for use in routine medication review in older people. In 2021, the British National Formulary recommended the use of STOPP/START criteria during routine medication review in older people as a means of identifying and minimizing inappropriate prescribing (IP) in this at-risk population [7]. STOPP/START criteria have also been implemented in the Dutch Multidisciplinary Guideline on polypharmacy in older people [8]. STOPP/START criteria have been validated in several populations in various clinical settings and assessed in relation to effectiveness in different settings in many different countries [9]. A number of published studies that reference STOPP/START criteria has increased steadily since 2008, indicating that STOPP/START criteria have practical clinical relevance in routine prescribing practice in many countries both in Europe and in many non-European countries.

With the expansion of the evidence base relating to pharmacotherapy in older people, it is acknowledged by most experts in geriatric pharmacotherapy that regular updating and revision of explicit PIM and PPO criteria are necessary. In recent years, updates of Beers criteria [10] and FORTA criteria [11] have also been published, reflecting advances in the evidence base relating to pharmacotherapy in older people. Furthermore, achieving the optimal blend of medication in older people often requires a combination of deprescribing PIMs and introduction of PPOs, a process referred to a ‘represcribing’ [12] that is facilitated by up-to-date explicit PIM and PPOs criteria.

Considering the substantial growth in the literature relating to PIMs and PPOs in older people 7 years on from the publication of STOPP/START version 2, it was expected that several new PIM and PPO criteria were required to reflect the continuing evolution of the evidence base relating to pharmacotherapy in older people. Accordingly, we aimed to revise and update STOPP/START criteria.

## Methods

We approached the third version of STOPP/START criteria with the following elements in sequence: (i) consensus on structure, (ii) revision of version 2 criteria for ongoing relevance and accuracy, (iii) a comprehensive review of the literature to support existing and proposed new criteria, and (iv) a Delphi validation exercise to achieve consensus on the list of proposed version 3 criteria generated from this consultation exercise. We agreed that the same systems-based design as had been used in the two previous versions of STOPP/START criteria would be maintained for version 3.

The approach taken was to proceed, system-by-system, to examine version 2 criteria and their evidence base and also for panel members to propose new criteria based on the published literature between April 2014 and March 2022, i.e., since the completion of version 2 criteria and the time limit on proposed version 3 criteria. First, we examined the 2015 STOPP/START version 2 criteria to assess if any criteria were no longer considered valid in routine prescribing practice. This was considered important to ensure that previously validated version 2 criteria were still appropriate to include in version 3 criteria and to remove any criteria that were outdated or no longer considered correct. Second, we assessed the various drug classes within each physiological system to determine if more recent treatment guidelines should be incorporated in version 3 criteria. Where it was clear that more recent treatment guidelines were available, these were adopted to support proposed new criteria. Third, each member of the Delphi panel was asked to propose any other new criteria that they considered important for consideration for Delphi validation in STOPP/START version 3.

For all criteria included in STOPP/START version 2 and to identify potential new criteria that reflect advances in pharmacotherapy since 2014, we undertook a comprehensive literature review, focusing on PubMed, Embase, and Cochrane Library on-line resources to identify the key references to support each proposed new criterion. As much as possible, we prioritized systematic reviews, meta-analyses, and randomized controlled clinical trials published in peer reviewed journals that supported the inclusion of each existing and newly proposed criterion, with two reviewers (DO’M and DC) examining the supporting references to ensure relevance (see on-line Supplement). The same reviewers evaluated STOPP/START version 2 to determine if any criteria were no longer considered correct or applicable according to the most up-to-date published evidence to 31 March 2022. They also examined each physiological system represented by the corresponding sections in STOPP and START criteria to identify potential new criteria based on published evidence since March 2014 (the time limit set for proposal of STOPP/START version 2 criteria). Within each physiological system, the most recently published literature relating to drug classes used to treat the most common disorders was evaluated as well as the evidence to support the novel use of certain drugs, such as SGLT-2 inhibitors (dapagliflozin, empagliflozin) in heart failure. If consensus on proposed new criteria could not be achieved, those criteria were not considered further for Delphi validation.

As with the previous versions of STOPP/START criteria, a Delphi validation process of the proposed criteria for STOPP/START version 3 was undertaken. For this purpose, an expert panel of 11 physicians with recognized academic profile in geriatric pharmacotherapy from 8 European countries was recruited (AC, ARG, MD, JBB, GO, AG, AJC, WK, GB, NV, MP). These physicians held senior academic appointments in their local university medical schools as well as being practicing clinicians.

Each panel member was presented with the full list of draft STOPP/ START version 3 criteria (*n *= 204, i.e., 145 STOPP and 59 START proposed criteria) and asked to review the criteria. In addition, each panel member was also invited to propose any additional new PIM or PPO criteria that they felt should be considered for adjudication if they considered them to be (i) clinically important, and (ii) clearly evidence based, and (iii) occurring frequently enough in clinical practice to justify possible inclusion in STOPP/START version 3 criteria.

For the Delphi validation exercise, we used the SurveyMonkey^®^ on-line platform. The criteria were presented as statements, with each statement rated on a 5-point Likert scale, ranging from 1 (strongly agree) to 5 (strongly disagree). Other points on the scale represented a range of opinion in between these diametric opposites, i.e., agree (=2), neutral (=3), and disagree (=4). Each panel member was asked to apply a Likert rating to each of the 145 STOPP and 59 START statements representing the proposed criteria. The rules for acceptance/inclusion of a proposed criterion were: (i) a median Likert value of 1 or 2, and (ii) a 75th centile Likert scale value that was not greater than 2.0. Proposed criteria with median Likert scale values greater than 2.0 were excluded. Proposed criteria with median Likert scale values of 2.0 but with a proportion in agreement of < 75% were re-assessed by the consensus panel in further Delphi validation rounds. We planned to continue sequential consensus Delphi rounds until clear accept/reject consensus was achieved on all proposed criteria.

## Results

The Delphi consensus panel judged 3 of the 114 STOPP/ START version 2 criteria to be obsolete or redundant and consequently these 3 criteria were removed, i.e., STOPP C2 and H7 and START B1. The specific details of these criteria were:

(i) STOPP C2: Aspirin with a previous history of peptic ulcer disease without concomitant PPI (risk of recurrent peptic ulcer).
(ii) STOPP H7: COX-2 selective NSAIDs with concurrent cardiovascular disease (increased risk of myocardial infarction and stroke).
(iii) START B1: Regular inhaled β-2 agonist or antimuscarinic bronchodilator (e.g., ipratropium, tiotropium) for mild to moderate asthma or COPD.

**Fig. 1:**
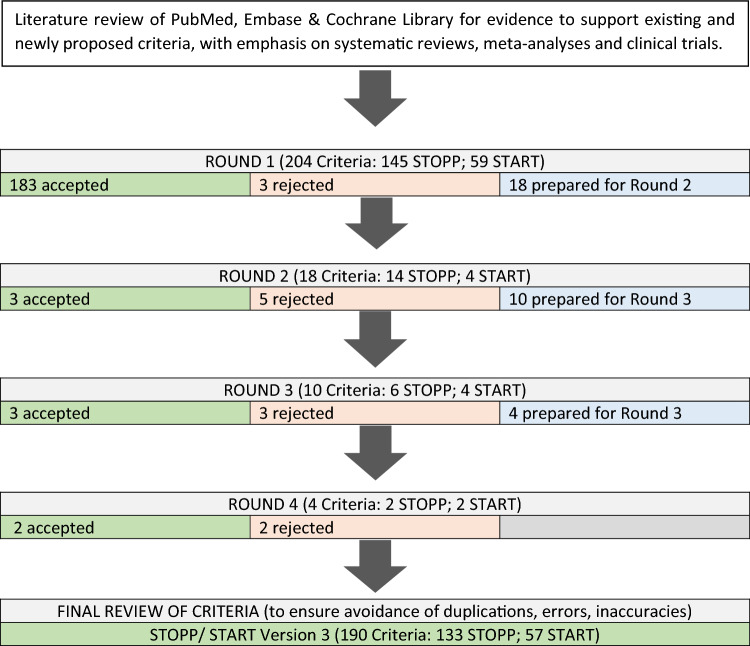
Flow diagram depicting Delphi validation process for validation of STOPP/START version 3 criteria

Ninety-three new STOPP/START criteria were recommended for Delphi validation. In consensus Round 1, 183 of the 204 proposed criteria (89.7%) were accepted for inclusion. Of the 21 proposed criteria that did not reach acceptance criteria by the expert panel, 3 criteria were rejected outright, and the remaining 18 criteria were presented to the expert panel for the second round of consensus validation. In consensus Round 2, 5 of the 18 criteria were accepted and 3 criteria were rejected by the consensus panel. The remaining ten criteria were considered in consensus Round 3, after which two criteria were accepted and four criteria were rejected. One of the remaining four proposed criteria was accepted for inclusion in consensus Round 4, and the remaining three proposed criteria were rejected; no further Delphi consensus rounds were required. Figure 1 summarizes the Delphi validation rounds process. The final total number of validated STOPP/START criteria was 190 (133 STOPP and 57 START criteria; see Appendix 1 for the criteria and Appendix 2 for the references of each criterion in the supplementary information). Compared to STOPP/START version 2, the number and percentage increase in criteria in each physiological system is shown in Table 1. Details of the rejected criteria are shown in Table 2.

**Table 1:** Proportionate changes in the numbers of STOPP and START criteria in version 3 compared to version 2.

Category	STOPP criteria (% change)	START criteria (% change)
Cardiovascular system	21 (61.5% increase)	11 (37.5% increase)
Coagulation system	16 (23.1% increase)	2 (new section in version 3)
Central nervous system	25 (78.6% increase)	7 (16.7% increase)
Renal system	10 (66.7% increase)	4 (new section in version 3)
Gastrointestinal system	8 (100.0% increase)	7 (350% increase)
Respiratory system	4 (0% increase)	3 (0% increase)
Musculoskeletal system	9 (0% increase)	9 (28.6% increase)
Urogenital system	8 (400% increase)	5 (66.7% increase)
Endocrine system	10 (66.7% increase)	1 (0% increase)
Falls risk increasing drugs	12 (300% increase)	Not applicable
Analgesic drugs	6 (100% increase)	3 (50% increase)
Vaccines	Not applicable	4 (100% increase)

**Table 2:** Proposed criteria rejected by the expert panel for inclusion in STOPP/START version 3 using Delphi consensus methodology

Rejected STOPP Criteria
Aspirin with a previous history of peptic ulcer disease without concomitant proton pump inhibitor (risk of recurrent peptic ulcer)
Mirabegron with known QTc interval prolongation (risk of exacerbation with associated ventricular arrhythmias)
Angiotensin converting enzyme inhibitors if eGFR < 30 ml/min/1.73m^2^ (risk of deterioration in renal function)
Angiotensin receptor blockers if eGFR < 30/min/1.73m^2^ (risk of deterioration in renal function)
Serotonin/noradrenaline reuptake inhibitors (SNRI’s e.g. venlafaxine, duloxetine) and chronic insomnia (likely to make insomnia worse)
Antiplatelet agents and Vitamin K antagonist, direct thrombin inhibitor or factor Xa inhibitors with a known history of cerebral amyloid angiopathy (increased risk of major intracerebral bleeding)
Thiazolidenediones (e.g. rosiglitazone, pioglitazone) with symptomatic hypotension (risk of exacerbation of hypotension)
Glucagon-like peptide-1 receptor (GLP-1) agonists (e.g. dulaglutide, exenatide, liraglutide, lixisenatide) with chronic constipation (risk of exacerbation of constipation)
Glucagon-like peptide-1 receptor (GLP-1) agonists (e.g. dulaglutide, exenatide, liraglutide, lixisenatide) with gastro-oesophageal reflux disease (risk of exacerbation of gastro-oesophageal reflux)
Mirabegron with current or previous tachyarrhythmia (risk of exacerbation or relapse of tachyarrhythmia)
Loop diuretics in patients with recurrent falls (may cause intravascular volume depletion and orthostatic hypotension)
*Rejected START Criteria*
Quinine sulphate in patients with recurrent painful lower limb muscle cramps
Memantine for moderate-severe Alzheimer’s disease

## Discussion

The substantially greater number of STOPP/START criteria in version 3 reflects the growth in published evidence between 2014 and 2022 relating to pharmacotherapy for common disorders encountered in older people such as heart disease, diabetes, and chronic obstructive pulmonary disease (COPD). The number of STOPP criteria has increased from 80 in version 2 to 133 in version 3, an 66.25% increase. Similarly, the number of START criteria has increased from 34 in version 2 to 57 in version 3, a 67.6% increase. The overall increase of STOPP/START criteria in version 3 compared to version 2, i.e., from 114 to 190 criteria represents a 66.7% increase. The growth in the overall number of STOPP/START criteria mostly reflects an expanding evidence base over the last decade.

Although expansion of therapeutic options for the treatment of common conditions in multimorbid older people is welcome, it carries increased possibilities of drug-related problems. The additional criteria in STOPP/START version 3 largely reflect the more common and important adverse drug–drug and drug–disease interactions encountered in current clinical practice. The increased number of criteria in version 3 is intended to assist clinicians to detect and prevent greater numbers of adverse drug–drug and drug–disease interactions and their consequences during routine medication review than in previous versions of STOPP/START.

This increased number of STOPP/START criteria does, however, present a challenge in terms of ease of application in routine medication review. Given the growing number of criteria from STOPP/START version 1 (87 criteria) to version 3 (190 criteria), deployment of the criteria using appropriate electronic applications is highly desirable to facilitate use. Without software applications to keep pace with the growing number of criteria, such an expansion in the number of criteria could inhibit their use in routine clinical practice. Previous multicentre clinical trials using STOPP/START criteria as an intervention, most notably the SENATOR and OPERAM trials [13, 14] have relied on bespoke electronic applications for deployment of STOPP/START version 2 criteria. Recent analysis of the specific STOPP and START criteria elicited in the OPERAM trial shows that over half of all the STOPP and START recommendations generated by the software tool were not  considered clinically relevant in individual cases [15]. Hence, there is a continuing need for electronically deployed STOPP and START criteria to be interpreted for clinical relevance by an appropriately trained healthcare professional [16].

Various clinical decision support software (CDSS) tools have been designed to optimize pharmacotherapy during medication review in older people with multimorbidity. A recent systematic review by Damoiseaux-Volman et al. [17] examined the impact of 18 different CDSS-based interventions on both process-related outcomes and patient-related clinical outcomes in this population. They concluded that CDSS interventions can clearly improve process-related outcomes but their effect on patient-related outcomes remains unclear. These findings further emphasize the need for CDSS-supported medication review to be coupled with trained clinical observation and interpretation for older patients and (where relevant) their carers. Another recent systematic review by Alshammari et al. [18] examining the role of explicit PIM criteria in the medication review of older hospitalized patients found that Beers criteria and STOPP/START criteria were the most widely used sets of explicit PIM criteria [22]. The same authors concluded that while explicit PIM detection tools such as Beers criteria and STOPP/START criteria were useful for the medication review process, their value in terms of securing better clinical outcomes and their cost-effectiveness remains uncertain. An earlier systematic review by Dalton et al. showed that CDSS-supported medication review can significantly reduce the prevalence of PIMs in hospitalized older patients [19]. However, once again, they concluded that it remains unclear whether reduction in PIM prevalence results in better patient outcomes such as fewer falls-related injuries or lower incidence of delirium.

The SENATOR and OPERAM multicentre trials [13, 14] used STOPP/START version 2 as a core component of the interventions tested. The focus in these trials was to examine the impact of a CDSS-based intervention on key patient-related endpoints, i.e., incident hospital-acquired adverse drug reactions (ADRs) in SENATOR and drug-related hospital readmissions in OPERAM. However, the impact of the interventions depended entirely on medication advice implementation in individual cases by attending physicians. The degree of implementation of STOPP and START criteria-related advice points was highly suboptimal in the intervention population of SENATOR, i.e., approximately 15%. In OPERAM, one or more STOPP/START version 2 recommendations was implemented in approximately 62% of patients in the intervention population where STOPP/START criteria were applicable, an improvement on the SENATOR trial, but still substantially suboptimal. These findings emphasize the need for trained physicians or pharmacists to interact with attending physicians to explain or qualify CDSS-generated STOPP and START criteria in individual cases and to provide advice as to clinical relevance of the specific criteria. The findings of one previous single-center, single-blinded clinical trial examining the effect of non-electronic application of STOPP/START version 1 criteria on incident ADRs during acute hospital admission in multimorbid older patients are noteworthy [20]. In that study, there was direct face-to-face contact between the physician primary researcher and the attending physicians to highlight and explain the clinical relevance of specific STOPP and START criteria. The implementation rates of STOPP and START criteria were 81% and 87%, respectively, with an associated highly significant reduction in hospital-acquired ADRs in the intervention patient cohort compared to the control cohort. Interestingly, there appears to be a significant difference in attending physician implementation of STOPP and START recommendations, depending on whether the recommendations are presented by a physician or a pharmacist, i.e., a higher implementation rate when presented by a physician [21].

Avoidance of PIMs should reduce the risk of potentially serious ADRs and adverse drug events (ADEs) (STOPP criteria). Similarly, avoidance of PPOs (START criteria) should reduce preventable morbidity such as major ischemic stroke through initiation of anticoagulants when untreated older patients have persistent atrial fibrillation. That is, the range of clinically relevant potential benefits to a heterogeneous population of older people with multimorbidity and associated polypharmacy remains very broad. The majority of the STOPP/START version 3 criteria are based on systematic review and clinical trial evidence. In the last decade, there has been substantial growth in the number of published clinical trials of a wide variety of drug therapies commonly prescribed to multimorbid older people. Nevertheless, there is no clear evidence that the rate of exclusion of older people from clinical trials in recent years has diminished compared to previous years [22]. Therefore, in routine clinical practice, prescribers should maintain caution when interpreting the relevance of clinical trial results to individual multimorbid older patients, particularly those who are highly frail. Nevertheless, the current updated version of STOPP/START criteria aims to reflect best practice in geriatric pharmacotherapy as supported by the expert consensus panel of senior academic physicians who participated in the Delphi process. The updated criteria continue to focus on situations that require caution in multimorbid older people, such as the prescribing of anticoagulants in patients with chronic atrial fibrillation and concurrent increased risk of bleeding (c.f. STOPP criteria C10, C12, C14). The degree of medication adjustment undertaken by prescribers when STOPP/START-defined PIMs and PPOs are identified will likely determine the clinical outcomes of older patients who experience multimorbidity and associated polypharmacy.

This Delphi validation exercise has a number of important strengths. All members of the Delphi validation panel were senior academic physicians in geriatric medicine with a special interest and expertise in pharmacotherapy. The eleven-member panel was drawn from eight countries representing northern, southern, eastern, and western Europe, i.e., a broad range of European clinical practice and perspective was represented in the Delphi panel. The updated criteria are evidence based and reflective of current European clinical therapeutics practice; very few proposed criteria were rejected by the panel. We contend that most of the common and clinically important PIMs and PPOs are incorporated into this new version of STOPP/START criteria, based primarily on our expert panel consensus.

Some limitations of the current Delphi validation process and outcomes are also acknowledged. First, a substantially larger version of STOPP/START than previously presents a challenge to application of the new version of the criteria in routine practice. Second, the consensus methodology deployed for the present study did not use live consensus panel meetings to discuss individual criteria as have been used for development of other sets of explicit PIM criteria, such as Beers criteria [10]. However, as in previous versions of STOPP/START criteria, the initial phase of criteria assessment was an examination of the most common and important instances of PIMs and PPOs encountered in current clinical practice followed by a careful examination of the literature to identify high-quality evidence to support the proposed criteria. Using an on-line consensus methodology instead of live consensus panel meetings had the advantage of avoiding any element of authority bias or group bias and to allow individual panel members to arrive at their own conclusions about proposed criteria based on the published evidence relating to each criterion. Finally, we did not employ formal systematic reviews to support individual criteria due to resource constraints.

In conclusion, we present a revised and updated version of STOPP/START explicit criteria to define clinically important potentially inappropriate medications and potential prescribing omissions relevant to medication review in older people. These criteria cannot replace clinical judgment in individual cases but may serve to guide physician prescribing and deprescribing practices and provide the basis for future clinical studies and interventions aimed at improving the quality of drug prescribing in older people. The increased number of STOPP/START version 3 criteria reflects both the growth in published evidence and the availability of several new medications to treat acute and chronic conditions in the last decade. Future studies should evaluate whether the application of these criteria can result in improved clinical outcomes for older patients.

## Supplementary Information

Below is the link to the electronic supplementary material.

Supplementary file 1 (PDF 525 KB) Appendix 1

Supplementary file 2 (DOCX 168 KB) Appendix 2

## Data Availability

The data relating to the Delphi validation process described in this article are available to external researchers on request.

## Declarations

### Disclaimer

In the preparation of STOPP/START criteria version 3, the authors have made every effort to ensure that all criteria as presented are accurate and evidence based. However, any and all decisions taken by prescribers on the initiation or avoidance of drugs or drug classes referred to in STOPP/START criteria version 3 are entirely the responsibility of the prescriber. We also emphasize that in the case of certain STOPP/START version 3 criteria, the evidence base for the criteria may change over time. For this reason, we recommend caution to prescribers to take account of the current evidence base relating to all drugs and medications referred to in individual STOPP/START version 3 criteria.
