# Correction: STOPP/START criteria for potentially inappropriate prescribing in older people: version 3

**DOI:** 10.1007/s41999-023-00812-y

**Published:** 2023-06-16

**Authors:** Denis O’Mahony, Antonio Cherubini, Anna Renom Guiteras, Michael Denkinger, Jean-Baptiste Beuscart, Graziano Onder, Adalsteinn Gudmundsson, Alfonso J. Cruz-Jentoft, Wilma Knol, Gülistan Bahat, Nathalie van der Velde, Mirko Petrovic, Denis Curtin

**Affiliations:** 1 Department of Medicine (Geriatric Medicine), School of Medicine, University College Cork, Cork, Ireland; 2 Department of Geriatric Medicine, Cork University Hospital, Wilton, Cork, Ireland; 3 Geriatria, Accettazione Geriatrica e Centro di Ricerca Per l’invecchiamento, IRCCS INRCA, Ancona, Italy; 4 Department of Geriatric Medicine, Parc de Salut Mar, Barcelona, Spain; 5 Institute for Geriatric Research, University of Ulm Geriatric Center, Alb-Donau, Ulm, Germany; 6 Univ. Lille, CHU Lille, ULR 2694 - METRICS: Évaluation des Technologies de Santé et des Pratiques Médicales, 59000 Lille, France; 7 Fondazione Policlinico Gemelli IRCCS and Università Cattolica del Sacro Cuore, Rome, Italy; 8 Landspitali University Hospital, Reykjavik, Iceland; 9 Servicio de Geriatría, Hospital Universitario Ramón y Cajal (IRYCIS), Madrid, Spain; 10 Department of Geriatric Medicine and Expertise Centre Pharmacotherapy in Old Persons (EPHOR), University Medical Center Utrecht, Utrecht University, Utrecht, The Netherlands; 11 Division of Geriatrics, Department of Internal Medicine, Istanbul Medical School, Istanbul University, Istanbul, Turkey; 12 Amsterdam UMC Location University of Amsterdam, Internal Medicine, Section of Geriatric Medicine and Amsterdam Public Health Research Institute, Amsterdam, The Netherlands; 13 Department of Internal Medicine and Paediatrics, Faculty of Medicine and Health Sciences, University of Ghent, Ghent, Belgium

**Correction: European Geriatric Medicine** 10.1007/s41999-023-00777-y

A supplementary file was missing from this article and has now been uploaded and the order of the supplementary files has been changed. In the second supplementary file, the title “Appendix 2” has been added. In addition, the reference to the supplementary files in the text has been amended to: ‘The final total number of validated STOPP/START criteria was 190 (133 STOPP and 57 START criteria; see Appendix 1 for the criteria and Appendix 2 for the references of each criterion in the supplementary information).’

The original article has been corrected.

## Supplementary Information

Below is the link to the electronic supplementary material.

Supplementary file 1 (PDF 525 KB) Appendix 1

Supplementary file 2 (DOCX 163 KB) Appendix 2

