# Supplementary material for: STOPP/START criteria for potentially inappropriate prescribing in older people: version 3
Source: Eur Geriatr Med. 2023 May 31;14(4):625–32. doi: 10.1007/s41999-023-00777-y (PMC10447584; doi:10.1007/s41999-023-00777-y)
Supplement: Supplementary file 1 — (PDF 525 KB) Appendix 1 [file 41999_2023_777_MOESM1_ESM.pdf]

Appendix 1

Screening Tool of Older Persons' Prescriptions (STOPP) version 3.

The following prescriptions are potentially inappropriate to use in patients aged 65 years and older.

Section A: Indication of medication

1. Any drug prescribed without a clinical indication.

2. Any drug prescribed beyond the recommended duration, where treatment duration is well defined.

3. Any duplicate drug class prescription for daily regular use (as distinct from PRN use) e.g., two concurrent NSAIDs, SSRIs, loop diuretics, ACE inhibitors, anticoagulants, antipsychotics, opioid analgesics (optimisation of monotherapy within a single drug class should be observed prior to considering a new agent).

Section B: Cardiovascular System

1. Digoxin for heart failure with normal systolic ventricular function (no clear evidence of benefit)

2. Verapamil or diltiazem with NYHA Class III or IV heart failure (may worsen heart failure with reduced ejection fraction i.e., HFREF).

3. Beta-blocker in combination with verapamil or diltiazem (risk of heart block).

4. Ventricular rate-limiting drugs i.e., beta blocker, verapamil, diltiazem, digoxin with bradycardia (< 50/min), type II heart block or complete heart block (risk of complete heart block, asystole).

5. Beta-blocker as monotherapy for uncomplicated hypertension i.e., not associated with angina pectoris, aortic aneurysm or other condition where beta-blocker therapy is indicated (no firm evidence of efficacy).

6. Amiodarone as first-line antiarrhythmic therapy in supraventricular tachyarrhythmias (higher risk of major side-effects than beta-blockers, digoxin, verapamil or diltiazem).

7. Loop diuretic as first-line treatment for hypertension unless there is concurrent heart failure requiring diuretic therapy (safer, more effective alternatives available).

8. Loop diuretic for dependent ankle oedema without clinical, biochemical or radiological evidence of heart failure, liver failure, nephrotic syndrome or renal failure (leg elevation and /or compression hosiery usually more appropriate).

9. Thiazide diuretic with current significant hypokalaemia (i.e., serum K+ < 3.0 mmol/l), hyponatraemia (i.e., serum Na+ < 130 mmol/l) hypercalcaemia (i.e., corrected serum calcium > 2.65 mmol/l) or with a history of gout (hypokalaemia, hyponatraemia, hypercalcaemia and gout can be precipitated by thiazide diuretic)

10. Loop diuretic for treatment of hypertension with concurrent urinary incontinence (may exacerbate incontinence).

11. Centrally-acting antihypertensives e.g., methyldopa, clonidine, moxonidine, rilmenidine, guanfacine (centrally-active antihypertensives are generally less well tolerated by older people than younger people)

12. Angiotensin-Converting Enzyme inhibitors (ACEIs) or Angiotensin Receptor Blockers (ARBs) in patients with hyperkalaemia i.e., serum K > 5.5 mmol/l.

13. Aldosterone antagonists (e.g., spironolactone, eplerenone) with concurrent potassium-conserving drugs (e.g., ACEI's, ARB's, amiloride, triamterene) without monitoring of serum potassium (risk of dangerous hyperkalaemia i.e., > 6.0 mmol/l – serum K should be monitored regularly, i.e., at least every 6 months).

14. Phosphodiesterase type-5 inhibitors (e.g., sildenafil, tadalafil, vardenafil) in severe heart failure characterised by hypotension i.e., systolic BP < 90 mmHg, or concurrent nitrate therapy for angina (risk of cardiovascular collapse).

15. Drugs that predictably prolong the QTc interval (QTc = QT/RR) in patients with known with demonstrable QTc prolongation (to >450 msec in males and >470 msec in females), including quinolones, macrolides, ondansetron, citalopram (doses > 20 mg/day), escitalopram (doses > 10 mg/day), tricyclic antidepressants, lithium, haloperidol, digoxin, class 1A antiarrhythmics, class III antiarrhythmics, tizanidine, phenothiazines, astemizole, mirabegron (risk of life-threatening ventricular arrhythmias).

16. Statins for primary cardiovascular prevention in persons aged ≥ 85 and established frailty with expected life expectancy likely less than 3 years (lack of evidence of efficacy).

17. Long-term systemic i.e., non-topical NSAIDs with known history of coronary, cerebral or peripheral vascular disease (increased risk of thrombosis).

18. Long-term antipsychotics with known history of coronary, cerebral or peripheral vascular disease (increased risk of thrombosis).

19. NSAIDs or systemic corticosteroids with heart failure requiring loop diuretic therapy (risk of exacerbation of heart failure).

20. Antihypertensive drugs in severe symptomatic aortic stenosis (risk of severe hypotension, syncope).

21. Digoxin as first line treatment for long-term (> 3 months) ventricular rate control in atrial fibrillation (increased mortality from long-term digoxin use; cardio-selective beta-blockers are generally preferable).

Section C: Coagulation System

1. Long-term aspirin at doses greater than 100mg per day (increased risk of bleeding, no evidence for increased efficacy).

2. Antiplatelet agents, vitamin K antagonists, direct thrombin inhibitors or factor Xa inhibitors with concurrent significant risk of major bleeding, i.e. uncontrolled severe hypertension, bleeding diathesis, recent non-trivial spontaneous bleeding (high risk of bleeding).

3. Aspirin plus clopidogrel as long-term secondary stroke prevention i.e., > 4 weeks, unless the patient has a coronary stent(s) inserted in the previous 12 months or concurrent acute coronary syndrome or has a high grade symptomatic carotid arterial stenosis (no evidence of added long-term benefit over clopidogrel monotherapy).

4. Antiplatelet agents in combination with vitamin K antagonist, direct thrombin inhibitor or factor Xa inhibitors in patients with chronic atrial fibrillation, unless there is concurrent coronary artery stent(s) inserted or angiographically proven high grade (> 50%) coronary artery stenosis (no added benefit from antiplatelet agents).

5. Antiplatelet agents with vitamin K antagonist, direct thrombin inhibitor or factor Xa inhibitors in patients with stable coronary, cerebrovascular or peripheral arterial disease (no evidence of added benefit from dual therapy).

6. Ticlopidine in any circumstances (clopidogrel and prasugrel have similar efficacy, stronger evidence and fewer side-effects).

7. Antiplatelet agents as alternatives to vitamin K antagonists, direct thrombin inhibitors or factor Xa inhibitors for stroke prevention in patients with chronic atrial fibrillation (no evidence of efficacy).

8. Vitamin K antagonist, direct thrombin inhibitor or factor Xa inhibitors for first deep venous thrombosis without continuing provoking risk factors (e.g., thrombophilia) for longer than 6 months, (no proven added benefit).

9. Vitamin K antagonist, direct thrombin inhibitor or factor Xa inhibitors for first pulmonary embolus without continuing provoking risk factors (e.g., thrombophilia) for longer than 12 months (no proven added benefit).

10. Nonsteroidal anti-inflammatory drugs (NSAIDs) and vitamin K antagonist, direct thrombin inhibitor or factor Xa inhibitors in combination (risk of major gastrointestinal bleeding).

11. Vitamin K antagonist as first-line anticoagulant for atrial fibrillation, unless there is concurrent metallic heart valve in-situ, moderate-to-severe mitral stenosis, or eGFR < 15 mls/min./1.73m^2 (direct thrombin inhibitors or factor Xa inhibitors are equally efficacious and safer than vitamin K antagonists).

12. Selective serotonin reuptake inhibitors (SSRIs) in combination with Vitamin K antagonist, direct thrombin inhibitor or factor Xa inhibitor with a previous history of major haemorrhage (increased risk of bleeding due to antiplatelet effects of SSRIs).

13. Direct thrombin inhibitor (e.g., dabigatran) and diltiazem or verapamil (increased risk of bleeding).

14. Apixaban, dabigatran, edoxaban, rivaroxaban and P-glycoprotein (P-gp) drug efflux pump inhibitors e.g., amiodarone, azithromycin, carvedilol, cyclosporin, dronedarone, itraconazole, ketoconazole (systemic), macrolides, quinine, ranolazine, tamoxifen, ticagrelor, verapamil (increased risk of bleeding).

15. Systemic oestrogens or androgens with pervious history of venous thromboembolism (increased risk of recurrent venous thromboembolism).

16. Aspirin for primary prevention in cardiovascular disease (no evidence of benefit).

Section D: Central Nervous System

1. TriCyclic Antidepressants (TCAs) in patients with dementia, narrow angle glaucoma, cardiac conduction abnormalities, prostatism, chronic constipation, recent falls, prior history of urinary retention or orthostatic hypotension (risk of worsening these conditions).

2. Initiation of TriCyclic Antidepressants (TCAs) as first-line treatment for major depression (higher risk of adverse drug reactions with TCAs than with SSRIs or SNRIs).

3. Serotonin/noradrenaline reuptake inhibitors (SNRI's e.g., venlafaxine, duloxetine) and severe hypertension i.e., systolic blood pressure > 180 mmHg +/- diastolic blood pressure > 105 mmHg (likely to make hypertension worse).

4. Antipsychotics with moderate-marked antimuscarinic/anticholinergic effects (acepromazine, chlorpromazine, clozapine, flupenthixol, fluphenzine, levomepromazine, olanzapine, pipothiazine, promazine, thioridazine) with a history of lower urinary tract symptoms associated with benign prostatic hyperplasia or previous urinary retention (high risk of urinary retention).

5. Antipsychotics prescribed for behavioural and psychological symptoms of dementia (BPSD) an unchanged dose for > 3 months without medication review (increased risk of extrapyramidal side-effects and chronic worsening of cognition, increased risk of major cardiovascular morbidity and mortality).

6. Selective serotonin re-uptake inhibitors (SSRI's) with current or recent significant hyponatraemia i.e., serum Na+ < 130 mmol/l (risk of exacerbating or precipitating hyponatraemia).

7. Selective serotonin re-uptake inhibitors (SSRI's) with current or recent significant bleeding (risk of exacerbation or recurrence of bleeding due to antiplatelet effects of SSRI's).

8. Benzodiazepines for ≥ 4 weeks (no indication for longer treatment; risk of prolonged sedation, confusion, impaired balance, falls, road traffic accidents; all benzodiazepines should be withdrawn gradually if taken for more than 4 weeks as there is a risk of causing a benzodiazepine withdrawal syndrome if stopped abruptly).

9. Benzodiazepines for agitated behaviour or psychotic symptoms of dementia (no evidence of efficacy).

10. Benzodiazepines for insomnia for ≥ 2 weeks (high risk of dependency, increased risk of falls, fractures and road traffic accidents).

11. Z-drugs (zolpidem, zopiclone, zaleplon) for insomnia for ≥ 2 weeks (increased risk of falls, fractures).

12. Antipsychotics (i.e., other than clozapine or quetiapine) in those with parkinsonism or Dementia with Lewy Bodies (risk of severe extra-pyramidal symptoms).

13. Anticholinergic/antimuscarinic drugs (biperiden, orphenadrine, procyclidine, trihexyphenidyl) to treat extra-pyramidal side-effects of antipsychotic medications (risk of anticholinergic toxicity).

14. Drugs with potent anticholinergics/antimuscarinic effects** in patients with delirium or dementia (risk of exacerbation of cognitive impairment).

** Commonly prescribed drugs with potent anticholinergic/ antimuscarinic effects include tricyclic antidepressants (e.g., amitriptyline, doxepin, impramine, nortriptyline), antipsychotics (chlorpromazine, clozapine, thioridazine), first generation antihistamines (e.g., diphenhydramine, chlorpheniramine), bladder anti-spasmodics (e.g., tolterodine, oxybutynin), hyoscine, procyclidine, benzatropine, tizanidine.

15. Antipsychotics in patients with behavioural and psychological symptoms of dementia (BPSD) for longer than 12 weeks unless BPSD symptoms are severe and other non-pharmacological treatments have failed (increased risk of stroke, myocardial infarction).

16. Antipsychotics as hypnotics, unless sleep disorder is due to psychosis or BPSD effects of dementia (not recommended in summary of product characteristics; increased risk of confusion, hypotension, extra-pyramidal side effects, falls).

17. Acetylcholinesterase inhibitors with a known history of persistent bradycardia (< 60 beats/min.), heart block or recurrent unexplained syncope (risk of cardiac conduction failure, syncope and injury).

18. Acetylcholinesterase inhibitors with concurrent treatment with drugs that induce persistent bradycardia (< 60 beats/min.) such as beta-blockers, digoxin, diltiazem, verapamil (risk of cardiac conduction failure, syncope and injury).

19. Memantine with known current or previous seizure disorder (increased risk of seizures).

20. Nootropics in dementia including Gingko Biloba, piracetam, pramiracetam, phenylpiracetam, aniracetam, phosphatidylserine, modafinil, L-theanine, omega-3 fatty acids, panax ginseng, rhodiola, creatine (no evidence of efficacy).

21. Phenothiazines as first-line treatment for psychosis or non-cognitive symptoms of dementia, since safer and more efficacious alternatives exist (phenothiazines are sedative, have significant anti-muscarinic toxicity in older people, except for prochlorperazine for

nausea/vomiting/vertigo, chlorpromazine for relief of persistent hiccoughs and levomepromazine as an anti-emetic in palliative care).

22. Levodopa or dopamine agonists for benign essential tremor (no evidence of efficacy)

23. Levodopa or dopamine agonists for treatment of extrapyramidal side-effects of antipsychotics or other forms of drug-induced Parkinsonism (inappropriate prescribing cascade to be avoided).

24. First-generation antihistamines as first-line treatment for allergy or pruritus (safer antihistamines with fewer side-effects now widely available).

25. First-generation antihistamines for insomnia (high risk of side-effects, Z-drugs safer and more appropriate for short-term use).

Section E: Renal System. The following commonly prescribed drugs are potentially inappropriate in older people with acute or chronic kidney disease with impaired renal function below particular levels of eGFR.

1. Digoxin at a long-term (i.e. more than 90 days) maintenance dose ≥ 125µg/day if eGFR < 30 ml/min/1.73m^2 (risk of digoxin toxicity if plasma levels not measured).

2. Direct thrombin inhibitors (e.g., dabigatran) if eGFR < 30 ml/min/1.73m^2 (risk of bleeding)

3. Factor Xa inhibitors (e.g., rivaroxaban, apixaban, edoxaban) if eGFR < 15 ml/min/1.73m^2 (risk of bleeding)

4. NSAID's if eGFR < 50 ml/min/1.73m^2 (risk of deterioration in renal function).

5. Colchicine if eGFR < 10 ml/min/1.73m^2 (risk of colchicine toxicity)

6. Metformin if eGFR < 30 ml/min/1.73m^2 (risk of lactic acidosis).

7. Mineralocorticoid receptor antagonists (e.g. spironolactone, eplerenone) if eGFR < 30 ml/min/1.73m^2 (risk of dangerous hyperkalaemia).

8. Nitrofurantoin if eGFR < 45 ml/min/1.73m^2 (increased risk of nitrofurantoin toxicity).

9. Bisphosphonates if eGFR<30 ml/min/1.73m^2 (increased risk of acute renal failure).

10. Methotrexate if eGFR <30 ml/min/1.73m^2 (increased risk of methotrexate toxicity).

Section F: Gastrointestinal System

1. Prochlorperazine or metoclopramide with Parkinsonism (risk of exacerbating Parkinsonian symptoms).

2. Proton pump inhibitor (PPI) for uncomplicated peptic ulcer disease at full therapeutic dosage for > 8 weeks (dose reduction or earlier discontinuation or H2 antagonist maintenance therapy usually indicated).

3. Drugs likely to cause constipation (e.g. systemic antimuscarinics, oral iron, opioids, verapamil, aluminium antacids) with chronic constipation where non-constipating alternatives are available (risk of exacerbation of constipation).

4. Oral elemental iron doses greater than 200 mg daily (e.g. ferrous fumarate> 600 mg/day, ferrous sulphate > 600 mg/day, ferrous gluconate> 1800 mg/day; no evidence of enhanced iron absorption above these doses).

5. Corticosteroids with a history of peptic ulcer disease or erosive oesophagitis (risk of relapse unless proton pump inhibitor is co-prescribed).

6. Antiplatelet or anticoagulant drugs with a history of Gastric Antral Vascular Ectasia (GAVE, “watermelon stomach”) (risk of major gastrointestinal bleeding).

7. Antipsychotics with dysphagia (increased risk of aspiration pneumonia).

8. Megestrol acetate to increase appetite (increased risk of thrombosis and death with unproven efficacy)

Section G: Respiratory System

1. Theophylline as monotherapy for COPD (safer, more effective alternative; risk of adverse effects due to narrow therapeutic index).

2. Systemic corticosteroids instead of inhaled corticosteroids for maintenance therapy in moderate-severe COPD (unnecessary exposure to long-term side-effects of systemic corticosteroids and effective inhaled therapies are available).

3. Long-acting muscarinic antagonists (e.g., tiotropium, aclidinium, umeclidinium, glycopyrronium) with a history of narrow angle glaucoma (may exacerbate glaucoma) or bladder outflow obstruction (may cause urinary retention).

4. Benzodiazepines with acute or chronic respiratory failure i.e. $pO_2 < 8.0$ kPa ± $pCO_2 > 6.5$ kPa (risk of exacerbation of respiratory failure).

Section H: Musculoskeletal System

1. Non-steroidal anti-inflammatory drugs (NSAIDs) other than COX-2 selective agents with history of peptic ulcer disease or gastrointestinal bleeding, unless with concurrent PPI or H2 antagonist (risk of peptic ulcer relapse).

2. NSAID’s with severe hypertension i.e., systolic blood pressure consistently above 170 mmHg and/or diastolic blood pressure consistently above 100 mmHg (risk of exacerbation of hypertension).

3. Long-term use of NSAID (>3 months) for symptom relief of osteoarthritis pain where paracetamol has not been tried (simple analgesics preferable and usually as effective for pain relief and safer).

4. Long-term corticosteroids (>3 months) as monotherapy for rheumatoid arthritis (risk of systemic corticosteroid side-effects).

5. Corticosteroids (other than periodic intra-articular injections for mono-articular pain) for osteoarthritis (risk of systemic corticosteroid side-effects).

6. Long-term NSAID or colchicine (>3 months) for chronic treatment of gout where there is no contraindication to a xanthine-oxidase inhibitor (e.g., allopurinol, febuxostat) (xanthine-oxidase inhibitors are first choice prophylactic drugs in gout).

7. NSAID with concurrent corticosteroids for treatment of arthritis/rheumatism of any kind (increased risk of peptic ulcer disease).

8. Oral bisphosphonates in patients with a current or recent history of upper gastrointestinal disease i.e. dysphagia, oesophagitis, gastritis, duodenitis, or peptic ulcer disease, or upper gastrointestinal bleeding (risk of relapse/exacerbation of oesophagitis, oesophageal ulcer, oesophageal stricture).

9. Long-term opioids for osteoarthritis (lack of evidence of efficacy, increased risk of serious side-effects).

Section I: Urogenital System

1. Systemic antimuscarinic drugs (e.g., oxybutynin, tolterodine, trospium) with dementia or chronic cognitive impairment (risk of increased confusion, agitation).

2. Systemic antimuscarinic drugs (e.g., oxybutynin, tolterodine, trospium) with narrow-angle glaucoma (risk of acute exacerbation of glaucoma).

3. Systemic antimuscarinic (e.g., oxybutynin, tolterodine, trospium) drugs for lower urinary tract symptoms with benign prostatic hyperplasia (BPH) and high post-void residual volume i.e. > 200 ml (uncertain efficacy and increased risk of urinary retention in older men).

4. Systemic antimuscarinic drugs (e.g., oxybutynin, tolterodine, trospium) with constipation (risk of exacerbation of constipation).

5. Alpha-1 receptor antagonists other than silodosin (e.g., alfuzosin, doxazosin, indoramin, tamsulosin, terazosin) with symptomatic orthostatic hypotension or history of syncope (risk of precipitating recurrent syncope).

6. Mirabegron in labile or severe hypertension (risk of exacerbation of hypertension).

7. Duloxetine with urinary urgency or urge incontinence (duloxetine is indicated in stress incontinence but not in urinary urgency or urge incontinence).

8. Antibiotic use in asymptomatic bacteriuria (no indication for treatment).

Section J. Endocrine System

1. Sulphonylureas with a long half-life (e.g., glibenclamide, chlorpropamide, glimepiride) with type 2 diabetes mellitus (risk of prolonged hypoglycaemia).

2. Thiazolidenediones (e.g., rosiglitazone, pioglitazone) with heart failure (risk of exacerbation of heart failure).

3. Non-selective beta-blockers in diabetes mellitus with frequent hypoglycaemic episodes (risk of suppressing hypoglycaemic symptoms).

4. Sodium glucose co-transporter (SGLT2) inhibitors (e.g., canagliflozin, dapagliflozin, empagliflozin, ertugliflozin) with symptomatic hypotension (risk of exacerbation of hypotension).

5. Systemic oestrogens with a history of breast cancer (increased risk of recurrence).

6. Systemic oestrogens with a history of venous thromboembolism (increased risk of recurrence).

7. Menopausal hormone therapy (oestrogen plus progestin) with a history of stenotic coronary, cerebral or peripheral arterial disease (increased risk of acute arterial thrombosis).

8. Systemic oestrogens without progestogens in patients with intact uterus (risk of endometrial cancer).

9. Levothyroxine in subclinical hypothyroidism i.e., normal free T4, elevated TSH but < 10 mU/L (no evidence of benefit, risk of iatrogenic thyrotoxicosis).

10. Vasopressin analogues (e.g., desmopressin, vasopressin) for urinary incontinence or urinary frequency (risk of symptomatic hyponatraemia).

Section K: Drug classes that predictably increase falls risk in susceptible older people.

1. Benzodiazepines in patients with recurrent falls (may cause reduced sensorium, impair balance).

2. Antipsychotic drugs in patients with recurrent falls (may cause Parkinsonism).

3. Vasodilator drugs in patients with recurrent falls with persistent postural hypotension i.e., systolic BP drop ≥ 20 mmHg and/or diastolic BP drop ≥ 10 mmHg (risk of syncope, falls).

4. Hypnotic Z-drugs i.e., zopiclone, zolpidem, zaleplon in patients with recurrent falls (may cause protracted daytime sedation, ataxia).

5. Anti-epileptic drugs in patients with recurrent falls (may impair sensorium, may adversely affect cerebellar function).

6. First generation antihistamines in patients with recurrent falls (may impair sensorium).

7. Opioids in patients with recurrent falls (may impair sensorium).

8. Antidepressants in patients with recurrent falls (may impair sensorium).

9. Alpha blockers as antihypertensives in patients with recurrent falls (may cause orthostatic hypotension).

10. Alpha blockers for prostatic bladder outflow symptoms, other than silodosin in patients with recurrent falls (may cause orthostatic hypotension).

11. Centrally acting antihypertensives (may impair sensorium and may cause orthostatic hypotension).

12. Antimuscarinics for treatment of overactive bladder or urge incontinence (may impair sensorium).

Section L: Analgesic Drugs

1. Use of oral or transdermal strong opioids (morphine, oxycodone, fentanyl, buprenorphine, diamorphine, methadone, tramadol, pethidine, pentazocine) as first line therapy for mild pain (WHO analgesic ladder not observed; paracetamol or NSAID not prescribed as first-line therapy).

2. Use of daily regular (as distinct from PRN) opioids without concomitant laxative (risk of severe constipation).

3. Long-acting opioids without short-acting opioids for break-through moderate or severe pain (risk of persistence of severe pain).

4. Topical lidocaine (lignocaine) patch for treatment of chronic osteoarthritis pain (lack of evidence of efficacy).

5. Gabapentinoids (e.g., gabapentin, pregabalin) for non-neuropathic pain (lack of evidence of efficacy).

6. Paracetamol at doses ≥ 3 g/24 hours in patients with poor nutritional status i.e., BMI < 18 or chronic liver disease (risk of hepatotoxicity).

Section M: Antimuscarinic/Anticholinergic Drug Burden

1. Concomitant use of two or more drugs with antimuscarinic/anticholinergic properties (e.g., bladder antispasmodics, intestinal antispasmodics, tricyclic antidepressants, first generation antihistamines, antipsychotics) (risk of increased antimuscarinic/anticholinergic toxicity).

Screening Tool to Alert to Right Treatment (START), version 3.

Unless an elderly patient's clinical status is end-of-life and therefore requiring a more palliative focus of pharmacotherapy, the following drug therapies should be considered where omitted for no valid clinical reason(s). It is assumed that the prescriber observes all the specific contraindications to these drug therapies prior to recommending them to older patients.

Section A: Indicated drugs.

1. Where a drug is clearly indicated and considered appropriate in the particular clinical context and there is no clear contraindication, that drug should be initiated as per formulary guidelines for dose and duration.

Section B: Cardiovascular System

1. Antihypertensive therapy where systolic blood pressure > 140 mmHg and /or diastolic blood pressure > 90 mmHg, unless established moderate or severe physical frailty in whom the threshold for therapy is 150 mmHg systolic pressure and/or 90 mmHg diastolic pressure.

2. Statin therapy with a documented history of coronary, cerebral or peripheral vascular disease, unless the patient's status is end-of-life or established moderate or severe frailty.

3. Angiotensin Converting Enzyme (ACE) inhibitor with coronary artery disease.

4. Beta-blocker with symptomatic coronary artery disease.

5. Angiotensin Converting Enzyme (ACE) inhibitor for heart failure with reduced ejection fraction.

6. Cardio-selective beta-blocker (bisoprolol, nebivolol, metoprolol or carvedilol) for stable heart failure with reduced ejection fraction.

7. Mineralocorticoid receptor antagonist (spironolactone, eplerenone) in heart failure without severe renal function impairment i.e., eGFR > 30 ml/min/m^2.

8. SGLT-2 inhibitors (canagliflozin, dapagliflozin, empagliflozin, ertugliflozin) in symptomatic heart failure with or without reduced ejection fraction regardless of diabetes being present or not.

9. Sacubitril/valsartan in heart failure with reduced ejection fraction causing persistent heart failure symptoms despite optimal dose of ACE inhibitor or Angiotensin Receptor Blocker (Sacubitril/valsartan to replace ACE inhibitor or Angiotensin Receptor Blocker).

10. Beta-blocker for chronic atrial fibrillation with uncontrolled heart rate.

11. Intravenous iron for symptomatic heart failure with reduced ejection fraction and iron deficiency.

Section C: Coagulation System

1. Vitamin K antagonists or direct thrombin inhibitors or factor Xa inhibitors in the presence of chronic or paroxysmal atrial fibrillation.

2. Antiplatelet therapy (aspirin or clopidogrel or prasugrel or ticagrelor) with a documented history of coronary, cerebral or peripheral vascular disease.

Section D: Central Nervous System

1. L-DOPA or a dopamine agonist in idiopathic Parkinson's disease with functional impairment and resultant disability.

2. Non-TCA antidepressant for major depression.

3. Acetylcholinesterase inhibitor (donepezil, rivastigmine, galantamine) for mild-moderate Alzheimer's dementia.

4. Rivastigmine for Dementia with Lewy Bodies or Parkinson's disease dementia.

5. Selective serotonin reuptake inhibitor (or SNRI or pregabalin if SSRI contraindicated) for persistent severe anxiety that affects independent functioning and quality of life.

6. Dopamine agonist (ropinirole or pramipexole or rotigotine) for Restless Legs Syndrome, once iron deficiency and severe chronic kidney disease (i.e., eGFR < 30 ml/min/m^2) have been excluded.

7. Propranolol for essential tremor with functional impairment and resultant disability.

Section E: Renal System

1. One-alpha hydroxycholecalciferol or calcitriol supplementation in severe chronic kidney (i.e., eGFR < 30 ml/min/m^2) disease with hypocalcaemia (corrected serum calcium < 2.10 mmol/l) and associated secondary hyperparathyroidism.

2. Phosphate binder in severe chronic kidney disease (i.e., eGFR < 30 ml/min/m2) if serum phosphate concentration persistently >1.76 mmol/l (5.5 mg/dl) despite adherence to renal diet.

3. Erythopoietin analogue in severe chronic kidney disease (i.e., eGFR < 30 ml/min/m^2) with symptomatic anaemia not attributable to haematinic or iron deficiency to achieve a haemoglobin concentration of 10.0 to 12.0 g/dl.

4. Angiotensin receptor blocker (ARB) or Angiotensin Converting Enzyme Inhibitor (ACE-I) in chronic kidney disease with proteinuria i.e., urine albumin excretion >300 mg/24 hours.

Section F: Gastrointestinal System

1. Proton Pump Inhibitor with severe gastro-oesophageal reflux disease or peptic oesophageal stricture requiring dilatation.

2. Proton pump inhibitor with initiation of low-dose aspirin and previous history of peptic ulcer or reflux oesophagitis.

3. Proton pump inhibitor with short-term (< 2 weeks) or longer-term (> 2 weeks) NSAID.

4. Fibre supplements (e.g., bran, ispaghula, methylcellulose, sterculia) for diverticulosis with a history of constipation.

5. Osmotic laxative (e.g., lactulose, macrogol, sorbitol) for chronic persistent idiopathic or secondary benign constipation.

6. Probiotics used with antibiotics in patients who are not immunocompromised or severely debilitated for the prevention of Clostridioides difficile-associated diarrhoea.

7. Helicobacter pylori eradication therapy in HP-associated active peptic ulcer disease.

Section G: Respiratory System

1. Long-acting muscarinic antagonist (LAMA e.g., tiotropium, aclidinium, umeclidinium, glycopyrronium) or long-acting beta 2 agonist (LABA e.g., bambuterol, formoterol, indacaterol, olodaterol, salmeterol) for symptomatic COPD of GOLD 1 or 2 severity and chronic asthma.

2. Regular i.e. daily inhaled corticosteroid (e.g., beclomethasone, budesonide, ciclesonide, fluticasone, mometasone) for moderate-severe asthma or COPD of GOLD 3 or 4 severity, where FEV1 <50% of predicted value and repeated exacerbations requiring treatment with oral corticosteroids.

3. Home continuous oxygen with documented chronic hypoxaemia (i.e., pO2 < 8.0 kPa or 60 mmHg or SaO2 < 89%).

Section H: Musculoskeletal System

1. Disease-modifying anti-rheumatic drug (DMARD) with chronic, active and disabling rheumatoid arthritis.

2. Bisphosphonates and vitamin D and calcium in patients taking long-term systemic corticosteroid therapy for prevention of steroid-induced osteoporosis.

3. Vitamin D in patients with known osteoporosis and/or previous fragility fracture(s) and/or Bone Mineral Density T-scores below -2.5 in one or multiple sites.

4. Bone anti-resorptive or anabolic therapy (e.g., bisphosphonate, teriparatide, denosumab) in patients with documented osteoporosis (Bone Mineral Density T-scores below -2.5 in one

or multiple sites) and/or previous history of fragility fracture(s) – where no pharmacological or clinical status contraindication exists such as poor one-year life expectancy.

5. Vitamin D supplement in older people with confirmed 25-hydroxycolecalciferol deficiency (< 20 micrograms/L, < 50 nmol/L) who are housebound or experiencing falls or with osteopenia (Bone Mineral Density T-score is less than -1.0 but above -2.5 in one or multiple sites).

6. Anti-resorptive treatment after discontinuation of at least two doses of denosumab (rebound increased bone turnover markers, BMD loss, and increased risk of vertebral fracture following denosumab discontinuation).

7. Anti-resorptive treatment after discontinuation of teriparatide/abaloparatide treatment for osteoporosis.

8. Xanthine-oxidase inhibitors (e.g. allopurinol, febuxostat) with a history of recurrent episodes of gout.

9. Folic acid supplement in patients taking methotrexate.

Section I: Urogenital System

1. Selective alpha-1 receptor blocker (e.g., tamsulosin, silodosin) for lower urinary tract symptoms related to benign prostatic hyperplasia where prostatectomy is not considered necessary or appropriate or safe.

2. 5-alpha reductase inhibitor (e.g., finasteride, dutasteride) for lower urinary tract symptoms related to benign prostatic hyperplasia where prostatectomy is not considered necessary or appropriate or safe.

3. Topical vaginal oestrogen or vaginal oestrogen pessary for symptomatic atrophic vaginitis.

4. Topical vaginal oestrogen or vaginal oestrogen pessary in women for recurrent urinary tract infections.

5. Phosphodiesterase type-5 inhibitors (e.g., avanafil, sildenafil, tadalafil, vardenafil) for persistent erectile dysfunction that causes distress.

Section J: Endocrine System

1. ACE inhibitor or Angiotensin Receptor Blocker (if intolerant of ACE inhibitor) in diabetes with evidence of renal disease i.e., dipstick proteinuria or microalbuminuria (>30 mg/24 hours) unless evidence of severe CKD (eGFR < 30 ml/min/m^2).

Section K: Analgesics

1. High-potency opioids in moderate-severe non-arthritis pain, where paracetamol, NSAIDs or low-potency opioids are not appropriate to the pain severity or have been ineffective.

2. Laxatives in patients receiving opioids regularly i.e., other than PRN use.

3. Topical 5% lidocaine (lignocaine) patch for localized neuropathic pain, e.g. post-herpetic neuralgia.

Section L: Vaccines

1. Seasonal influenza vaccine annually.

2. Pneumococcal vaccine at least once according to national guidelines.

3. Varicella-zoster vaccine according to national guidelines.

4. SARS-CoV2 vaccine according to national guidelines.
