# Supplementary material for: STOPP/START criteria for potentially inappropriate prescribing in older people: version 3
Source: Eur Geriatr Med. 2023 May 31;14(4):625–32. doi: 10.1007/s41999-023-00777-y (PMC10447584; doi:10.1007/s41999-023-00777-y)
Supplement: Supplementary file 2 — (DOCX 168 KB) Appendix 2 [file 41999_2023_777_MOESM2_ESM.docx]

**Appendix 2**

**STOPP Criteria Version 3 References**

**Section A: Drug indication criteria**

**A1. Any drug prescribed without an evidence-based clinical indication.**

No references (self-evident)

**A2. Any drug prescribed beyond the recommended duration, where treatment duration is well defined.**

No references (self-evident)

**A3. Any duplicate drug class prescription for daily regular use (as distinct from PRN use) e.g. two concurrent NSAIDs, SSRIs, loop diuretics, ACE inhibitors, anticoagulants, antipsychotics, opioid analgesics (optimisation of monotherapy within a single drug class should be observed prior to considering a new agent).**

A3 (i): Olsson J, Bergman A, Carlsten A, Oké T, Bernsten C, Schmidt IK, Fastbom J. Quality of drug prescribing in elderly people in nursing homes and special care units for dementia: a cross-sectional computerized pharmacy register analysis. Clin Drug Investig 2010; 30(5): 289-300. PubMed PMID: 20384385.

A3 (ii): Martin BC, Wiley-Exley EK, Richards S, Domino ME, Carey TS, Sleath BL. Contrasting measures of adherence with simple drug use, medication switching, and therapeutic duplication. Ann Pharmacother 2009; 43(1): 36-44. PubMed PMID: 19126828.

A3 (iii): Laurier C, Moride Y, Kennedy WA. Health survey data on potentially inappropriate geriatric drug use. Ann Pharmacother 2002; 36(3): 404-9. PubMed PMID: 11895051.

**Section B: Cardiovascular System criteria**

**B1. Digoxin for heart failure with preserved systolic ventricular function (no clear evidence of benefit)**

B1 (i). Jessup M, Abraham WT, Casey DE, Feldman AM, Francis GS, Ganiats TG, Konstam MA, Mancini DM, Rahko PS, Silver MA, Stevenson LW, Yancy CW. 2009 focused update: ACCF/AHA Guidelines for the Diagnosis and Management of Heart Failure in Adults: a report of the American College of Cardiology Foundation/American Heart Association Task Force on Practice Guidelines: developed in collaboration with the International Society for Heart and Lung Transplantation. Circulation 2009; 119(14): 1977-2016. PubMed PMID: 19324967.

B1 (ii): Cheng JW, Nayar M. A review of heart failure management in the elderly population. Am J Geriatr Pharmacother 2009; 7(5): 233-49. Review. PubMed PMID: 19948300.

**B2. Verapamil or diltiazem with NYHA Class III or IV heart failure (may worsen heart failure with reduced ejection fraction i.e. HFREF).**

B2 (i): Amabile CM, Spencer AP. Keeping your patient with heart failure safe: a review of potentially dangerous medications. Arch Intern Med 2004; 164(7): 709-20. Review. PubMed PMID:15078640.

B2 (ii): Opie LH, Yusuf S, Kübler W. Current status of safety and efficacy of calcium channel blockers in cardiovascular diseases: a critical analysis based on 100 studies. Prog Cardiovasc Dis 2000; 43(2): 171-96. Review. PubMed PMID:11014332.

**B3. Beta-blocker in combination with verapamil or diltiazem (risk of heart block).**

B3 (i): British National Formulary No. 81, March - September 2021. BMJ Group & the Royal Pharmaceutical Society of Great Britain 2021, volume 81, page 1430.

B3 (ii): Edoute Y, Nagachandran P, Svirski B, Ben-Ami H. Cardiovascular adverse drug reaction associated with combined beta-adrenergic and calcium entry-blocking agents. J Cardiovasc Pharmacol 2000; 35(4): 556-9. PubMed PMID: 10774785.

**B4. Ventricular rate-limiting drugs i.e. beta blocker, verapamil, diltiazem, digoxin with bradycardia (< 50/min), type II heart block or complete heart block (risk of profound hypotension, asystole).**

B4 (i). British National Formulary, No. 81, March - September 2021. BMJ Group the Royal Pharmaceutical Society of Great Britain, pages 107-118.

**B5. Beta-blocker as monotherapy for uncomplicated hypertension i.e. not associated with angina pectoris, aortic aneurysm or other condition where beta-blocker therapy is indicated (no firm evidence of efficacy).**

B5 (i): Wiysonge CS, Bradley HA, Volmink J, Mayosi BM, Opie LH. Beta-blockers for hypertension. Cochrane Database Syst Rev. 2017 Jan 20;1(1):CD002003. doi: 10.1002/14651858.CD002003.pub5. PMID: 28107561.

B5 (ii): Thomopoulos C, Bazoukis G, Tsioufis C, Mancia G. Beta-blockers in hypertension: overview and meta-analysis of randomized outcome trials. J Hypertens. 2020 Sep;38(9):1669-1681. doi: 10.1097/HJH.0000000000002523. PMID: 32649628.

**B6: Amiodarone as first-line antiarrhythmic therapy in supraventricular tachyarrhythmias (higher risk of major side-effects than beta-blockers, digoxin, verapamil or diltiazem)**

B6 (i): Lafuente-Lafuente C, Mouly S, Longás-Tejero MA, Mahé I, Bergmann JF. Antiarrhythmic drugs for maintaining sinus rhythm after cardioversion of atrial fibrillation: a systematic review of randomized controlled trials. Arch Intern Med 2006; 166(7):719-28. Review. PubMed PMID: 16606807.

B6 (ii): Piccini JP, Berger JS, O'Connor CM. Amiodarone for the prevention of sudden cardiac death: a meta-analysis of randomized controlled trials. Eur Heart J 2009; 30(10):1245-53. Review. PubMed PMID: 19336434.

**B7. Loop diuretic as first-line treatment for hypertension unless there is concurrent heart failure requiring diuretic therapy (lack of outcome data for this indication; safer, more effective alternatives available).**

B7 (i): Sica DA, Carter B, Cushman W, Hamm L. Thiazide and loop diuretics. J Clin Hypertens (Greenwich) 2011; 13(9): 639-43. Review. PubMed PMID: 21896142.

B7 (ii). Williams B, Poulter NR, Brown MJ, Davis M, McInnes GT, Potter JF, Sever PS, Thom SM; BHS guidelines working party, for the British Hypertension Society. British Hypertension Society guidelines for hypertension management 2004(BHS-IV): summary. BMJ 2004; 328(7440):634-40. Erratum in: BMJ 2004; 328(7445):926. PubMed PMID: 15016698.

**B8. Loop diuretic for dependent ankle oedema without clinical, biochemical evidence or radiological evidence of heart failure, liver failure, nephrotic syndrome or renal failure (leg elevation and /or compression hosiery usually more appropriate).**

B8 (i): Wehling M. Morbus diureticus in the elderly: epidemic overuse of a widely applied group of drugs. J Am Med Dir Assoc 2013; 14(6): 437-42. Review. PubMed PMID: 23510827.

B8 (ii). Sarafidis PA, Georgianos PI, Lasaridis AN. Diuretics in clinical practice. Part I: mechanisms of action, pharmacological effects and clinical indications of diuretic compounds. Expert Opin Drug Saf 2010; 9(2):243-57. Review. PubMed PMID: 20095917.

**B9. Thiazide diuretic with current significant hypokalaemia (i.e. serum K+ < 3.0 mmol/l), hyponatraemia (i.e. serum Na+ < 130 mmol/l) hypercalcaemia (i.e. corrected serum calcium > 2.65 mmol/l) or with a history of gout (hypokalaemia, hyponatraemia, hypercalcaemia and gout can be precipitated by thiazide diuretic).**

B9 (i).Sica DA, Carter B, Cushman W, Hamm L. Thiazide and loop diuretics. J Clin Hypertens (Greenwich) 2011; 13(9):639-43. Review. PubMed PMID: 21896142.

B9 (ii). Gurwitz JH, Kalish SC, Bohn RL, Glynn RJ, Monane M, Mogun H, Avorn J. Thiazide diuretics and the initiation of anti-gout therapy. J Clin Epidemiol 1997; 50(8): 953-9. PubMed PMID: 9291881.

**B10. Loop diuretic for treatment of hypertension with concurrent urinary incontinence (may exacerbate incontinence).**

B10 (i). Ekundayo OJ. The association between overactive bladder and diuretic use in the elderly. Curr Urol Rep 2009; 10(6):434-40. Review. PubMed PMID: 19863854.

B10 (ii) Ekundayo OJ, Markland A, Lefante C, Sui X, Goode PS, Allman RM, Ali M, Wahle C, Thornton PL, Ahmed A. Association of diuretic use and overactive bladder syndrome in older adults: a propensity score analysis. Arch Gerontol Geriatr 2009; 49(1):64-8. PubMed PMID: 18752858.

B10 (iii) Finkelstein MM. Medical conditions, medications, and urinary incontinence. Analysis of a population-based survey. Can Fam Physician 2002; 48:96-101.PubMed PMID: 11852617.

**B11. Centrally-acting antihypertensives (e.g. methyldopa, clonidine, moxonidine, rilmenidine, guanfacine), unless clear intolerance of, or lack of efficacy with, other classes of antihypertensives (centrally-active antihypertensives are generally less well tolerated by older people than younger people).**

B11 (i). Potter JF. Hypertension. In: Brocklehurst’s Textbook of Geriatric Medicine & Gerontology, 6^th^ edition, Churchill Livingstone, 2003, p403.

B11 (ii). Khindri S, Jackson S. Hypertension. In: Prescribing for Elderly Patients, S. Jackson, P. Jansen, A. Mangoni, eds., Wiley-Blackwell, Chichester, UK, 2009, pp97-98.

**B12. Angiotensin-Converting Enzyme (ACE) inhibitors or Angiotensin Receptor Blockers (ARBs) in patients with hyperkalaemia i.e. serum K > 5.5 mmol/L.**

B12 (i) Izzo JL Jr, Weir MR. Angiotensin-converting enzyme inhibitors. J Clin Hypertens (Greenwich) 2011; 13(9):667-75. Review. PubMed PMID: 21896148.

B12 (ii) Desai AS, Swedberg K, McMurray JJ, Granger CB, Yusuf S, Young JB, Dunlap ME, Solomon SD, Hainer JW, Olofsson B, Michelson EL, Pfeffer MA; CHARM Program Investigators. Incidence and predictors of hyperkalemia in patients with heart failure: an analysis of the CHARM Program. J Am Coll Cardiol 2007 Nov 13;50(20):1959-66. PubMed PMID: 17996561.

B12 (iii): Reardon LC, Macpherson DS. Hyperkalemia in outpatients using angiotensin-converting enzyme inhibitors. How much should we worry? Arch Intern Med 1998; 158(1):26-32. PubMed PMID: 9437375.

**B13. Aldosterone antagonists (e.g. spironolactone, eplerenone) with concurrent potassium-conserving drugs (e.g. ACEI’s, ARB’s, amiloride, triamterene) without monitoring of serum potassium (risk of dangerous hyperkalaemia i.e. > 6.0 mmol/l – serum K should be monitored regularly, i.e. at least every 6 months).**

B13 (i): Bauersachs J, Fraccarollo D. Aldosterone antagonism in addition to angiotensin-converting enzyme inhibitors in heart failure. Minerva Cardioangiol 2003; 51(2):155-64. Review. PubMed PMID: 12783071.

B13 (ii): Poggio R, Grancelli HO, Miriuka SG. Understanding the risk of hyperkalaemia in heart failure: role of aldosterone antagonism. Postgrad Med J 2010; 86 (1013):136-42. Review. PubMed PMID: 20237007.

B13 (iii) Marcy TR, Ripley TL. Aldosterone antagonists in the treatment of heart failure. Am J Health Syst Pharm 2006; 63(1): 49-58. PubMed PMID: 16373465.

B13 (iv): Tang WH, Parameswaran AC, Maroo AP, Francis GS. Aldosterone receptor antagonists in the medical management of chronic heart failure. Mayo Clin Proc 2005; 80(12): 1623-30. Review. PubMed PMID: 16342656.

**B14. Phosphodiesterase type-5 inhibitors (e.g. sildenafil, tadalafil, vardenafil) in severe heart failure characterised by hypotension i.e. systolic BP < 90 mmHg, or concurrent daily nitrate therapy for angina (risk of cardiovascular collapse)**

B14 (i): British National Formulary, No. 81, March – September 2021, BMJ Group & Pharmaceutical Press, pages 858-863.

B14 (ii): Kloner RA, Hutter AM, Emmick JT, Mitchell MI, Denne J, Jackson G. Time course of the interaction between tadalafil and nitrates. J Am Coll Cardiol 2003; 42 (10): 1855-60. PubMed PMID: 14642699.

**B15: Drugs that predictably prolong the QTc interval (QTc = QT/RR) in patients with known with known QTc prolongation (to >450 msec in males and >470 msec in females), including quinolones, macrolides, ondansetron, citalopram (doses > 20 mg/day), escitalopram (doses > 10 mg/day), tricyclic antidepressants, lithium, haloperidol, digoxin, class 1A antiarrhythmics, class III antiarrhythmics, tizanidine, phenothiazines, astemizole, mirabegron (risk of life-threatening ventricular arrhythmias).**

B15 (i): Skullbacka S, Airaksinen M, Puustinen J, Toivo T. Risk assessment tools for QT prolonging pharmacotherapy in older adults: a systematic review. Eur J Clin Pharmacol. 2022 May;78(5):765-779. doi: 10.1007/s00228-022-03285-3. Epub 2022 Feb 14. PMID: 35156131; PMCID: PMC9005415.

B15 (ii): Fazio G, Vernuccio F, Grutta G, Re GL. Drugs to be avoided in patients with long QT syndrome: Focus on the anaesthesiological management. World J Cardiol. 2013 Apr 26;5(4):87-93. doi: 10.4330/wjc.v5.i4.87. PMID: 23675554

**B16: Statins for primary cardiovascular prevention in persons aged ≥ 85 years (lack of evidence of efficacy) and established frailty with expected life expectancy less than 3 years.**

B16 (i): Cholesterol Treatment Trialists' (CTT) Collaborators, Mihaylova B, Emberson J, Blackwell L, Keech A, Simes J, Barnes EH, Voysey M, Gray A, Collins R, Baigent C. The effects of lowering LDL cholesterol with statin therapy in people at low risk of vascular disease: meta-analysis of individual data from 27 randomised trials. Lancet. 2012 Aug 11;380(9841):581-90. doi: 10.1016/S0140-6736(12)60367-5. Epub 2012 May 17. PMID: 22607822.

B16 (ii): Chou R, Dana T, Blazina I, Daeges M, Jeanne TL. Statins for Prevention of Cardiovascular Disease in Adults: Evidence Report and Systematic Review for the US Preventive Services Task Force. JAMA. 2016 Nov 15; 316(19):2008-2024. doi: 10.1001/jama.2015.15629. Erratum in: JAMA. 2020 Feb 18;323(7):669. PMID: 27838722.

B16 (iii): Marcellaud E, Jost J, Tchalla A, Magne J, Aboyans V. Statins in Primary Prevention in People Over 80 Years. Am J Cardiol. 2023 Jan 15; 187:62-73. doi: 10.1016/j.amjcard.2022.10.015. Epub 2022 Nov 29. PMID: 36459749.

**B17: Long-term systemic i.e. non-topical NSAIDs with known history of coronary, cerebral or peripheral vascular disease (increased risk of thrombosis).**

B17 (i): McGettigan P, Henry D. Cardiovascular risk and inhibition of cyclooxygenase: a systematic review of the observational studies of selective and nonselective inhibitors of cyclooxygenase 2. JAMA. 2006 Oct 4;296(13):1633-44. doi: 10.1001/jama.296.13.jrv60011. Epub 2006 Sep 12. PMID: 16968831.

B17 (ii): McGettigan P, Henry D. Cardiovascular risk with non-steroidal anti-inflammatory drugs: systematic review of population-based controlled observational studies. PLoS Med. 2011 Sep;8(9):e1001098. doi: 10.1371/journal.pmed.1001098. Epub 2011 Sep 27. PMID: 21980265.

B17 (iii): Coxib and traditional NSAID Trialists' (CNT) Collaboration, Bhala N, Emberson J, Merhi A, Abramson S, Arber N, Baron JA, Bombardier C, Cannon C, Farkouh ME, FitzGerald GA, Goss P, Halls H, Hawk E, Hawkey C, Hennekens C, Hochberg M, Holland LE, Kearney PM, Laine L, Lanas A, Lance P, Laupacis A, Oates J, Patrono C, Schnitzer TJ, Solomon S, Tugwell P, Wilson K, Wittes J, Baigent C. Vascular and upper gastrointestinal effects of non-steroidal anti-inflammatory drugs: meta-analyses of individual participant data from randomised trials. Lancet. 2013 Aug 31;382(9894):769-79. doi: 10.1016/S0140-6736(13)60900-9. Epub 2013 May 30. PMID: 23726390.

**B18: Long-term antipsychotics with known history of coronary, cerebral or peripheral vascular disease (increased risk of thrombosis).**

B18 (i): Yu ZH, Jiang HY, Shao L, Zhou YY, Shi HY, Ruan B. Use of antipsychotics and risk of myocardial infarction: a systematic review and meta-analysis. Br J Clin Pharmacol. 2016 Sep;82(3):624-32. doi: 10.1111/bcp.12985. Epub 2016 May 23. PMID: 27198162.

B18 (ii): Foley DL, Morley KI. Systematic review of early cardiometabolic outcomes of the first treated episode of psychosis. Arch Gen Psychiatry. 2011 Jun;68(6):609-16. doi: 10.1001/archgenpsychiatry.2011.2. Epub 2011 Feb 7. PMID: 21300937.

**B19: NSAIDs or systemic corticosteroids with heart failure requiring loop diuretic therapy (risk of exacerbation of heart failure**).

B19 (i): Coxib and traditional NSAID Trialists' (CNT) Collaboration, Bhala N, Emberson J, Merhi A, Abramson S, Arber N, Baron JA, Bombardier C, Cannon C, Farkouh ME, FitzGerald GA, Goss P, Halls H, Hawk E, Hawkey C, Hennekens C, Hochberg M, Holland LE, Kearney PM, Laine L, Lanas A, Lance P, Laupacis A, Oates J, Patrono C, Schnitzer TJ, Solomon S, Tugwell P, Wilson K, Wittes J, Baigent C. Vascular and upper gastrointestinal effects of non-steroidal anti-inflammatory drugs: meta-analyses of individual participant data from randomised trials. Lancet. 2013 Aug 31;382(9894):769-79. doi: 10.1016/S0140-6736(13)60900-9. Epub 2013 May 30. PMID: 23726390.

B19 (ii): Arfè A, Scotti L, Varas-Lorenzo C, Nicotra F, Zambon A, Kollhorst B, Schink T, Garbe E, Herings R, Straatman H, Schade R, Villa M, Lucchi S, Valkhoff V, Romio S, Thiessard F, Schuemie M, Pariente A, Sturkenboom M, Corrao G; Safety of Non-steroidal Anti-inflammatory Drugs (SOS) Project Consortium. Non-steroidal anti-inflammatory drugs and risk of heart failure in four European countries: nested case-control study. BMJ. 2016 Sep 28;354:i4857. doi: 10.1136/bmj.i4857. PMID: 27682515.

B19 (iii): Souverein PC, Berard A, Van Staa TP, Cooper C, Egberts AC, Leufkens HG, Walker BR. Use of oral glucocorticoids and risk of cardiovascular and cerebrovascular disease in a population based case-control study. Heart. 2004 Aug;90(8):859-65. doi: 10.1136/hrt.2003.020180. PMID: 15253953.

**B20. Antihypertensive drugs in severe symptomatic aortic stenosis except for RAS inhibitors (risk of severe hypotension, syncope).**

B20 (i): Kang TS, Park S. Antihypertensive Treatment in Severe Aortic Stenosis. J Cardiovasc Imaging. 2018 Jun;26(2):45-53. doi: 10.4250/jcvi.2018.26.e9. Epub 2018 Jun 22. PMID: 29971263.

B20 (ii): Saeed S, Mancia G, Rajani R, Parkin D, Chambers JB. Antihypertensive treatment with calcium channel blockers in patients with moderate or severe aortic stenosis: Relationship with all-cause mortality. Int J Cardiol. 2020 Jan 1;298:122-125. doi: 10.1016/j.ijcard.2019.09.007. Epub 2019 Sep 7. PMID: 31575497.

**B21. Digoxin as first line treatment for long-term (> 3 months) ventricular rate control in atrial fibrillation (increased mortality from long-term digoxin use; cardio-selective beta-blockers are generally preferable).**

B21 (i): Lopes RD, Rordorf R, De Ferrari GM, Leonardi S, Thomas L, Wojdyla DM, Ridefelt P, Lawrence JH, De Caterina R, Vinereanu D, Hanna M, Flaker G, Al-Khatib SM, Hohnloser SH, Alexander JH, Granger CB, Wallentin L; ARISTOTLE Committees and Investigators. Digoxin and Mortality in Patients With Atrial Fibrillation. J Am Coll Cardiol. 2018 Mar 13;71(10):1063-1074. doi: 10.1016/j.jacc.2017.12.060. PMID: 29519345.

B21 (ii): Vamos M, Erath JW, Hohnloser SH. Digoxin-associated mortality: a systematic review and meta-analysis of the literature. Eur Heart J. 2015 Jul 21;36(28):1831-8. doi: 10.1093/eurheartj/ehv143. Epub 2015 May 4. PMID: 25939649.

**Section C: Coagulation System criteria**

**C1. Long-term aspirin at doses greater than 100mg per day (increased risk of bleeding, no evidence for increased efficacy).**

C1 (i). Dalen JE. Aspirin to prevent heart attack and stroke: what's the right dose?

Am J Med 2006; 119(3):198-202. Review. PubMed PMID: 16490462.

C1 (ii). Fisher M, Knappertz V. The dose of aspirin for the prevention of cardiovascular and cerebrovascular events. Curr Med Res Opin 2006; 22(7):1239-48. Review. PubMed PMID: 16892516.

**C2. Antiplatelet agents, vitamin K antagonists, direct thrombin inhibitors or factor Xa inhibitors with concurrent significant bleeding risk, i.e. uncontrolled severe hypertension, bleeding diathesis, recent non-trivial spontaneous bleeding (high risk of bleeding).**

C2 (i): Lip GY. Implications of the CHA(2)DS(2)-VASc and HAS-BLED Scores for thromboprophylaxis in atrial fibrillation. Am J Med. 2011; 124(2):111-4. PubMed PMID: 20887966.

C2 (ii): Pisters R, Lane DA, Nieuwlaat R, de Vos CB, Crijns HJ, Lip GY. A novel user-friendly score (HAS-BLED) to assess 1-year risk of major bleeding in patients with atrial fibrillation: the Euro Heart Survey. Chest 2010; 138(5):1093-100. PubMed PMID: 20299623.

**C3. Aspirin plus clopidogrel as long-term secondary stroke prevention i.e. >4 weeks, unless the patient has a coronary stent(s) inserted in the previous 12 months or concurrent acute coronary syndrome or has a high grade symptomatic carotid arterial stenosis (no evidence of long-term benefit over clopidogrel monotherapy)**

C3 (i): Diener HC, Bogousslavsky J, Brass LM, Cimminiello C, Csiba L, Kaste M, Leys D, Matias-Guiu J, Rupprecht HJ; MATCH investigators. Aspirin and clopidogrel compared with clopidogrel alone after recent ischaemic stroke or transient ischaemic attack in high-risk patients (MATCH): randomised, double-blind, placebo-controlled trial. Lancet 2004; 364(9431):331-7. PubMed PMID:

15276392.

C3 (ii): Bhatt DL, Fox KA, Hacke W, Berger PB, Black HR, Boden WE, Cacoub P, Cohen EA, Creager MA, Easton JD, Flather MD, Haffner SM, Hamm CW, Hankey GJ, Johnston SC, Mak KH, Mas JL, Montalescot G, Pearson TA, Steg PG, Steinhubl SR, Weber MA, Brennan DM, Fabry-Ribaudo L, Booth J, Topol EJ; CHARISMA Investigators. Clopidogrel and aspirin versus aspirin alone for the prevention of atherothrombotic events. N Engl J Med. 2006; 354(16):1706-17. PubMed PMID: 16531616.

C3 (iii): Usman MH, Notaro LA, Nagarakanti R, Brahin E, Dessain S, Gracely E, Ezekowitz MD. Combination antiplatelet therapy for secondary stroke prevention: enhanced efficacy or double trouble? Am J Cardiol 2009;103(8):1107-12. Review. PubMed PMID: 19361598.

C3 (iv): Squizzato A, Keller T, Romualdi E, Middeldorp S. Clopidogrel plus aspirin versus aspirin alone for preventing cardiovascular disease. Cochrane Database Syst Rev 2011;(1):CD005158. Review. PubMed PMID: 21249668.

C3 (v): Fares RR, Lansing LS, Gallati CA, Mousa SA. Antiplatelet therapy with clopidogrel and aspirin in vascular diseases: clinical evidence for and against the combination. Expert Opin Pharmacother 2008; 9(3): 377-86. Review. PubMed PMID: 18220489.

**C4. Antiplatelet agents in combination with vitamin K antagonist, direct thrombin inhibitor or factor Xa inhibitors in patients with chronic atrial fibrillation , unless there is concurrent coronary artery stent(s) inserted or angiographically proven high grade (> 50%) coronary artery stenosis (no added benefit from antiplatelet agents).**

C4 (i): Flaker GC, Gruber M, Connolly SJ, Goldman S, Chaparro S, Vahanian A, Halinen MO, Horrow J, Halperin JL; SPORTIF Investigators. Risks and benefits of combining aspirin with anticoagulant therapy in patients with atrial fibrillation: an exploratory analysis of stroke prevention using an oral thrombin inhibitor in atrial fibrillation (SPORTIF) trials. Am Heart J 2006; 152(5):967-73. PubMed PMID: 17070169.

C4 (ii): Larson RJ, Fisher ES. Should aspirin be continued in patients started on warfarin? J Gen Intern Med 2004; 19(8):879-86. Review. PubMed Central PMCID: PMC1492499.

**C5. Antiplatelet agents with vitamin K antagonist, direct thrombin inhibitor or factor Xa inhibitors in patients with stable coronary, cerebrovascular or peripheral arterial disease without a clear indication for anticoagulant therapy (no added benefit from dual therapy).**

C5 (i): Holmes DR Jr, Kereiakes DJ, Kleiman NS, Moliterno DJ, Patti G, Grines CL. Combining antiplatelet and anticoagulant therapies. J Am Coll Cardiol 2009; 54(2):95-109. Review. PubMed PMID: 19573725.

C5 (ii): Rubboli A, Halperin JL, Airaksinen KE, Buerke M, Eeckhout E, Freedman SB, Gershlick AH, Schlitt A, Tse HF, Verheugt FW, Lip GY. Antithrombotic therapy in patients treated with oral anticoagulation undergoing coronary artery stenting. An expert consensus document with focus on atrial fibrillation. Ann Med 2008; 40(6):428-36. Review. PubMed PMID: 18608125.

**C6. Ticlopidine in any circumstances (clopidogrel and prasugrel have similar efficacy, stronger evidence and fewer side-effects).**

C6 (i): Furie KL, Kasner SE, Adams RJ, Albers GW, Bush RL, Fagan SC, Halperin JL, Johnston SC, Katzan I, Kernan WN, Mitchell PH, Ovbiagele B, Palesch YY, Sacco RL, Schwamm LH, Wassertheil-Smoller S, Turan TN, Wentworth D; American Heart Association Stroke Council, Council on Cardiovascular Nursing, Council on Clinical Cardiology, and Interdisciplinary Council on Quality of Care and Outcomes Research. Guidelines for the prevention of stroke in patients with stroke or transient ischemic attack: a guideline for healthcare professionals from the American heart association/American stroke association. Stroke 2011; 42(1):227-76. PubMed PMID: 20966421.

C6 (ii): Porto I, Giubilato S, De Maria GL, Biasucci LM, Crea F. Platelet P2Y12 receptor inhibition by thienopyridines: status and future. Expert Opin Investig Drugs 2009; 18(9):1317-32. Review. PubMed PMID: 19678800.

**C7. Antiplatelet agents as alternatives to vitamin K antagonists, direct thrombin inhibitors or factor Xa inhibitors for stroke prevention in patients with chronic atrial fibrillation (no evidence of efficacy).**

C7 (i): Hart RG, Pearce LA, Aguilar MI. Meta-analysis: antithrombotic therapy to prevent stroke in patients who have nonvalvular atrial fibrillation. Ann Intern Med. 2007 Jun 19;146(12):857-67. doi: 10.7326/0003-4819-146-12-200706190-00007. PMID: 17577005.

C7 (ii): Aguilar MI, Hart R, Pearce LA. Oral anticoagulants versus antiplatelet therapy for preventing stroke in patients with non-valvular atrial fibrillation and no history of stroke or transient ischemic attacks. Cochrane Database Syst Rev. 2007 Jul 18;(3):CD006186. doi: 10.1002/14651858.CD006186.pub2. PMID: 17636831.

**C8: Vitamin K antagonist, direct thrombin inhibitor or factor Xa inhibitors for first deep venous thrombosis without continuing provoking risk factors for longer than 6 months, (no proven added benefit).**

C8 (i): Pinede L, Ninet J, Duhaut P, Chabaud S, Demolombe-Rague S, Durieu I, Nony P, Sanson C, Boissel JP; Investigators of the "Durée Optimale du Traitement AntiVitamines K" (DOTAVK) Study. Comparison of 3 and 6 months of oral anticoagulant therapy after a first episode of proximal deep vein thrombosis or pulmonary embolism and comparison of 6 and 12 weeks of therapy after isolated calf deep vein thrombosis. Circulation 2001; 103(20): 2453-60. PubMed PMID: 11369685.

C8 (ii): Kearon C, Akl EA, Comerota AJ, Prandoni P, Bounameaux H, Goldhaber SZ, Nelson ME, Wells PS, Gould MK, Dentali F, Crowther M, Kahn SR; American College of Chest Physicians. Antithrombotic therapy for VTE disease: Antithrombotic Therapy and Prevention of Thrombosis, 9th ed: American College of Chest Physicians Evidence-Based Clinical Practice Guidelines. Chest 2012; 141(2 Suppl): e419S-94S. PubMed PMID: 22315268.

**C9. Vitamin K antagonist, direct thrombin inhibitor or factor Xa inhibitors for first pulmonary embolus without continuing provoking risk factors for longer than 6 months (no proven added benefit).**

C9 (i): Kearon C, Akl EA, Comerota AJ, Prandoni P, Bounameaux H, Goldhaber SZ, Nelson ME, Wells PS, Gould MK, Dentali F, Crowther M, Kahn SR; American College of Chest Physicians. Antithrombotic therapy for VTE disease: Antithrombotic Therapy and Prevention of Thrombosis, 9th ed: American College of Chest Physicians Evidence-Based Clinical Practice Guidelines. Chest 2012; 141(2 Suppl): e419S-94S. PubMed PMID: 22315268; PubMed Central PMCID: PMC3278049.

**C10. NSAID and vitamin K antagonist, direct thrombin inhibitor or factor Xa inhibitors in combination (risk of gastrointestinal bleeding).**

C10 (i): Knijff-Dutmer EA, Van der Palen J, Schut G, Van de Laar MA. The influence of cyclo-oxygenase specificity of non-steroidal anti-inflammatory drugs on bleeding complications in concomitant coumarin users. QJM 2003; 96(7):513-20. PubMed PMID: 12881594.

C10 (ii): Peng S, Duggan A. Gastrointestinal adverse effects of non-steroidal anti-inflammatory drugs. Expert Opin Drug Saf 2005; 4(2):157-69. Review. PubMed PMID: 15794710.

**C11: Vitamin K antagonist as first-line anticoagulant for atrial fibrillation, unless there is concurrent metallic heart valve in-situ, moderate-to-severe mitral stenosis, or creatinine clearance less than 15mls/ min (direct thrombin inhibitor or factor Xa inhibitors are equally efficacious and safer than vitamin K antagonists).**

C11 (i): Ruff CT, Giugliano RP, Braunwald E, Hoffman EB, Deenadayalu N, Ezekowitz MD, Camm AJ, Weitz JI, Lewis BS, Parkhomenko A, Yamashita T, Antman EM. Comparison of the efficacy and safety of new oral anticoagulants with warfarin in patients with atrial fibrillation: a meta-analysis of randomised trials. Lancet. 2014 Mar 15;383(9921):955-62. doi: 10.1016/S0140-6736(13)62343-0. Epub 2013 Dec 4. PMID: 24315724.

C11 (ii): Bruins Slot KM, Berge E. Factor Xa inhibitors versus vitamin K antagonists for preventing cerebral or systemic embolism in patients with atrial fibrillation. Cochrane Database Syst Rev. 2018 Mar 6;3(3):CD008980. doi: 10.1002/14651858.CD008980.pub3. PMID: 29509959.

C11 (iii): Salazar CA, del Aguila D, Cordova EG. Direct thrombin inhibitors versus vitamin K antagonists for preventing cerebral or systemic embolism in people with non-valvular atrial fibrillation. Cochrane Database Syst Rev. 2014 Mar 27;2014(3):CD009893. doi: 10.1002/14651858.CD009893.pub2. PMID: 24677203.

**C12: Selective serotonin reuptake inhibitors in combination with Vitamin K antagonist, direct thrombin inhibitor or factor Xa inhibitors with a previous history of major haemorrhage (increased risk of bleeding due to antiplatelet effects of SSRIs).**

C12 (i): Nochaiwong S, Ruengorn C, Awiphan R, Chai-Adisaksopha C, Tantraworasin A, Phosuya C, Kanjanarat P, Chongruksut W, Sood MM, Thavorn K. Use of serotonin reuptake inhibitor antidepressants and the risk of bleeding complications in patients on anticoagulant or antiplatelet agents: a systematic review and meta-analysis. Ann Med. 2022 Dec;54(1):80-97. doi: 10.1080/07853890.2021.2017474. PMID: 34955074.

C12 (ii): Laporte S, Chapelle C, Caillet P, Beyens MN, Bellet F, Delavenne X, Mismetti P, Bertoletti L. Bleeding risk under selective serotonin reuptake inhibitor (SSRI) antidepressants: A meta-analysis of observational studies. Pharmacol Res. 2017 Apr;118:19-32. doi: 10.1016/j.phrs.2016.08.017. Epub 2016 Aug 10. PMID: 27521835.

**C13: Direct thrombin inhibitor (e.g. dabigatran) and diltiazem or verapamil (increased risk of bleeding).**

C13 (i): Pham P, Schmidt S, Lesko L, Lip GYH, Brown JD. Association of Oral Anticoagulants and Verapamil or Diltiazem With Adverse Bleeding Events in Patients With Nonvalvular Atrial Fibrillation and Normal Kidney Function. JAMA Netw Open. 2020 Apr 1;3(4):e203593. Observational study. doi: 10.1001/jamanetworkopen.2020.3593. PMID: 32329770.

C13 (ii): Härtter S, Sennewald R, Nehmiz G, Reilly P. Oral bioavailability of dabigatran etexilate (Pradaxa(®) ) after co-medication with verapamil in healthy subjects. Br J Clin Pharmacol. 2013 Apr;75(4):1053-62. doi: 10.1111/j.1365-2125.2012.04453.x. PMID: 22946890.

**C14: Apixaban, dabigatran, edoxaban, rivaroxaban and P-glycoprotein (P-gp) drug efflux pump inhibitors e.g. amiodarone, azithromycin, carvedilol, cyclosporin, dronedarone, itraconazole, ketoconazole (systemic), macrolides, quinine, ranolazine, tamoxifen, ticagrelor, verapamil (increased risk of bleeding).**

C14 (i): Gronich N, Stein N, Muszkat M. Association Between Use of Pharmacokinetic-Interacting Drugs and Effectiveness and Safety of Direct Acting Oral Anticoagulants: Nested Case-Control Study. Clin Pharmacol Ther. 2021 Dec;110(6):1526-1536. doi: 10.1002/cpt.2369. Epub 2021 Aug 10. PMID: 34287842.

C14 (ii): British National Formulary, volume 81, March-September 2021. BMJ Group & The Royal Pharmaceutical Press of Great Britain 2021, pp 1513-4.

**C15: Systemic oestrogens or androgens with pervious history of venous thromboembolism (increased risk of recurrent venous thromboembolism).**

C15 (i) Canonico M, Plu-Bureau G, Lowe GD, Scarabin PY. Hormone replacement therapy and risk of venous thromboembolism in postmenopausal women: systematic review and meta-analysis. BMJ. 2008 May 31;336(7655):1227-31. doi: 10.1136/bmj.39555.441944.BE. Epub 2008 May 20. PMID: 18495631.

C15 (ii): Walker RF, Zakai NA, MacLehose RF, Cowan LT, Adam TJ, Alonso A, Lutsey PL. Association of Testosterone Therapy With Risk of Venous Thromboembolism Among Men With and Without Hypogonadism. JAMA Intern Med. 2020 Feb 1;180(2):190-197. doi: 10.1001/jamainternmed.2019.5135. PMID: 31710339.

**C16: Aspirin for primary prevention of cardiovascular disease.**

C16 (i): Zheng SL, Roddick AJ. Association of Aspirin Use for Primary Prevention With Cardiovascular Events and Bleeding Events: A Systematic Review and Meta-analysis. JAMA. 2019 Jan 22;321(3):277-287. doi: 10.1001/jama.2018.20578. Erratum in: JAMA. 2019 Jun 11;321(22):2245. PMID: 30667501.

C16 (ii): Gelbenegger G, Postula M, Pecen L, Halvorsen S, Lesiak M, Schoergenhofer C, Jilma B, Hengstenberg C, Siller-Matula JM. Aspirin for primary prevention of cardiovascular disease: a meta-analysis with a particular focus on subgroups. BMC Med. 2019 Nov 4;17(1):198. doi: 10.1186/s12916-019-1428-0. PMID: 31679516.

**Section D: Central Nervous System criteria**

**D1. Tricyclic antidepressants in patients with dementia, narrow angle glaucoma, cardiac conduction abnormalities, lower urinary tract symptoms related to benign prostatic hyperplasia, chronic constipation, recent falls, or prior history of urinary retention (risk of worsening these conditions).**

D1 (i): Mintzer J, Burns A. Anticholinergic side-effects of drugs in elderly people. J R Soc Med 2000; 93 (9):457-62. Review. PubMed PMID: 11089480.

D1 (ii): Verhamme KM, Sturkenboom MC, Stricker BH, Bosch R. Drug-induced urinary retention: incidence, management and prevention. Drug Saf 2008; 31(5):373-88. Review. PubMed PMID: 18422378.

D1 (iii): Vieweg WV, Wood MA, Fernandez A, Beatty-Brooks M, Hasnain M, Pandurangi AK. Proarrhythmic risk with antipsychotic and antidepressant drugs: implications in the elderly. Drugs Aging 2009; 26(12): 997-1012. Review. PubMed PMID: 19929028.

D1 (iv): Tripathi RC, Tripathi BJ, Haggerty C. Drug-induced glaucomas: mechanism and management. Drug Saf 2003; 26(11): 749-67. Review. PubMed PMID: 12908846.

D1 (v): Bhanu C, Nimmons D, Petersen I, Orlu M, Davis D, Hussain H, Magammanage S, Walters K. Drug-induced orthostatic hypotension: A systematic review and meta-analysis of randomised controlled trials. PLoS Med. 2021 Nov 9;18(11):e1003821. doi: 10.1371/journal.pmed.1003821. PMID: 34752479; PMCID: PMC8577726.

**D2. Initiation of tricyclic antidepressants as first-line treatment for major depression (higher risk of adverse drug reactions with TCAs than with SSRIs or SNRIs).**

D2 (i): Emslie G, Judge R. Tricyclic antidepressants and selective serotonin reuptake inhibitors: use during pregnancy, in children/adolescents and in the elderly. Acta Psychiatr Scand Suppl. 2000; 403: 26-34. Review. PubMed PMID: 11019932.

D2 (ii) Mottram P, Wilson K, Strobl J. Antidepressants for depressed elderly. Cochrane Database Syst Rev 2006 Jan 25; (1):CD003491. Review. PubMed PMID: 16437456.

**D3. Serotonin/noradrenaline reuptake inhibitors (SNRI’s e.g. venlafaxine, duloxetine) and severe hypertension i.e. systolic blood pressure > 180 mmHg +/- diastolic blood pressure > 105 mmHg (likely to make hypertension worse).**

D3 (i): Zhong Z, Wang L, Wen X, Liu Y, Fan Y, Liu Z. A meta-analysis of effects of selective serotonin reuptake inhibitors on blood pressure in depression treatment: outcomes from placebo and serotonin and noradrenaline reuptake inhibitor controlled trials. Neuropsychiatr Dis Treat. 2017 Nov 7;13:2781-2796. doi: 10.2147/NDT.S141832. PMID: 29158677.

D3 (ii): Thase ME. Effects of venlafaxine on blood pressure: a meta-analysis of original data from 3744 depressed patients. J Clin Psychiatry. 1998 Oct;59(10):502-8. doi: 10.4088/jcp.v59n1002. PMID: 9818630.

D3 (iii): Derby MA, Zhang L, Chappell JC, Gonzales CR, Callaghan JT, Leibowitz M, Ereshefsky L, Hoelscher D, Leese PT, Mitchell MI. The effects of supratherapeutic doses of duloxetine on blood pressure and pulse rate. J Cardiovasc Pharmacol. 2007 Jun;49(6):384-93. doi: 10.1097/FJC.0b013e31804d1cce. PMID: 17577103.

**D4. Antipsychotics with moderate-marked antimuscarinic/anticholinergic effects (acepromazine, chlorpromazine, clozapine, flupenthixol, fluphenzine, levomepromazine, olanzapine, pipothiazine, promazine, thioridazine) with a history of lower urinary tract symptoms associated with benign prostatic hyperplasia or previous urinary retention (high risk of urinary retention).**

D4 (i): Verhamme KM, Sturkenboom MC, Stricker BH, Bosch R. Drug-induced urinary retention: incidence, management and prevention. Drug Saf 2008; 31(5):373-88. Review. PubMed PMID: 18422378.

D4 (ii): British National Formulary, volume 81, March-September 2021. BMJ Group & The Royal Pharmaceutical Press of Great Britain 2021, p 1431.

**D5: Antipsychotics prescribed for behavioural and psychological. symptoms of dementia (BPSD) at an unchanged dose for > 3 months without medication review (increased risk of extrapyramidal side-effects and chronic worsening of cognition, increased risk of major cardiovascular morbidity and mortality).**

D5 (i): [Sink KM, Holden KF, Yaffe K. Pharmacological treatment of neuropsychiatric symptoms of dementia: a review of the evidence. JAMA 2005; 293:596.](https://www.uptodate.com/contents/management-of-neuropsychiatric-symptoms-of-dementia/abstract/50)

D5 (ii): [Lee PE, Gill SS, Freedman M, et al. Atypical antipsychotic drugs in the treatment of behavioural and psychological symptoms of dementia: systematic review. BMJ 2004; 329:75.](https://www.uptodate.com/contents/management-of-neuropsychiatric-symptoms-of-dementia/abstract/88)

D5 (iii): [Wang PS, Schneeweiss S, Avorn J, et al. Risk of death in elderly users of conventional vs. atypical antipsychotic medications. N Engl J Med 2005; 353:2335.](https://www.uptodate.com/contents/management-of-neuropsychiatric-symptoms-of-dementia/abstract/99)

D5 (iv): [Schneider LS, Dagerman KS, Insel P. Risk of death with atypical antipsychotic drug treatment for dementia: meta-analysis of randomized placebo-controlled trials. JAMA 2005; 294:1934.](https://www.uptodate.com/contents/management-of-neuropsychiatric-symptoms-of-dementia/abstract/97)

**D6. Selective serotonin re-uptake inhibitors (SSRI’s) with current or recent significant hyponatraemia i.e. serum Na+ < 130 mmol/l (risk of exacerbating or precipitating hyponatraemia).**

D6 (i): Jacob S, Spinler SA. Hyponatremia associated with selective serotonin-reuptake inhibitors in older adults. Ann Pharmacother 2006; 40(9):1618-22. Review. PubMed PMID: 16896026.

D6 (ii): Draper B, Berman K. Tolerability of selective serotonin reuptake inhibitors: issues relevant to the elderly. Drugs Aging 2008; 25(6): 501-19. Review. PubMed PMID: 18540689.

**D7. Selective serotonin re-uptake inhibitors (SSRI’s) with current or recent significant bleeding (risk of exacerbation or recurrence of bleeding due to antiplatelet effects of SSRI’s).**

D7 (i): Laporte S, Chapelle C, Caillet P, Beyens MN, Bellet F, Delavenne X, Mismetti P, Bertoletti L. Bleeding risk under selective serotonin reuptake inhibitor (SSRI) antidepressants: A meta-analysis of observational studies. Pharmacol Res. 2017 Apr; 118:19-32. doi: 10.1016/j.phrs.2016.08.017. Epub 2016 Aug 10. PMID: 27521835.

D7 (ii): Hackam DG, Mrkobrada M. Selective serotonin reuptake inhibitors and brain hemorrhage: a meta-analysis. Neurology. 2012 Oct 30;79(18):1862-5. doi: 10.1212/WNL.0b013e318271f848. Epub 2012 Oct 17. PMID: 23077009.

D7 (iii): Jiang HY, Chen HZ, Hu XJ, Yu ZH, Yang W, Deng M, Zhang YH, Ruan B. Use of selective serotonin reuptake inhibitors and risk of upper gastrointestinal bleeding: a systematic review and meta-analysis. Clin Gastroenterol Hepatol. 2015 Jan;13(1):42-50.e3. doi: 10.1016/j.cgh.2014.06.021. Epub 2014 Jun 30. PMID: 24993365.

**D8. Benzodiazepines for ≥ 4 weeks (no indication for longer treatment; risk of prolonged sedation, confusion, impaired balance, falls, road traffic accidents; all benzodiazepines should be withdrawn gradually if taken for > 2 weeks as there is a risk of causing a benzodiazepine withdrawal syndrome if stopped abruptly).**

D8 (i): Madhusoodanan S, Bogunovic OJ. Safety of benzodiazepines in the geriatric population. Expert Opin Drug Saf 2004; 3(5): 485-93. Review. PubMed PMID:15335303.

D8 (ii): Glass J, Lanctôt KL, Herrmann N, Sproule BA, Busto UE. Sedative hypnotics in older people with insomnia: meta-analysis of risks and benefits. BMJ 2005; 331(7526): 1169. Review. PubMed PMID: 16284208.

D8 (iii): Barker MJ, Greenwood KM, Jackson M, Crowe SF. Cognitive effects of long-term

benzodiazepine use: a meta-analysis. CNS Drugs 2004; 18(1):37-48. PubMed PMID: 14731058.

**D9. Benzodiazepines for agitated behaviour or non-cognitive symptoms of dementia (no evidence of efficacy).**

D9 (i): Tampi RR, Tampi DJ. Efficacy and tolerability of benzodiazepines for the treatment of behavioral and psychological symptoms of dementia: a systematic review of randomized controlled trials. Am J Alzheimers Dis Other Demen. 2014 Nov;29(7):565-74. doi: 10.1177/1533317514524813. PMID: 25551131.

D9 (ii): Defrancesco M, Marksteiner J, Fleischhacker WW, Blasko I. Use of Benzodiazepines in Alzheimer's Disease: A Systematic Review of Literature. Int J Neuropsychopharmacol. 2015 May 19;18(10):pyv055. doi: 10.1093/ijnp/pyv055. PMID: 25991652; PMCID: PMC4648159.

**D10. Benzodiazepines for insomnia for ≥ 2 weeks (high risk of dependency, increased risk of falls, fractures and road traffic accidents).**

D10 (i): Gerlach LB, Wiechers IR, Maust DT. Prescription Benzodiazepine Use Among Older Adults: A Critical Review. Harv Rev Psychiatry. 2018 Sep/Oct;26(5):264-273. doi: 10.1097/HRP.0000000000000190. PMID: 30188338.

D10 (ii): Woolcott JC, Richardson KJ, Wiens MO, et al. Meta-analysis of the impact of 9 medication classes on falls in elderly persons. Arch Intern Med. 2009;169:1952–60.

D10 (iii) Barbone F, McMahon AD, Davey PG, et al. Association of road-traffic accidents with benzodiazepine use. Lancet 1998; 352:1331–6.

**D11. Z-drugs (zolpidem, zopiclone, zaleplon) for insomnia for ≥ 2 weeks (increased risk of falls, fractures).**

D11 (i): Scharner V, Hasieber L, Sönnichsen A, Mann E. Efficacy and safety of Z-substances in the management of insomnia in older adults: a systematic review for the development of recommendations to reduce potentially inappropriate prescribing. BMC Geriatr. 2022 Feb 1;22(1):87. doi: 10.1186/s12877-022-02757-6. PMID: 35100976; PMCID: PMC9887772.

D11 (ii): Machado FV, Louzada LL, Cross NE, Camargos EF, Dang-Vu TT, Nóbrega OT. More than a quarter century of the most prescribed sleeping pill: Systematic review of zolpidem use by older adults. Exp Gerontol. 2020 Jul 15;136:110962. doi: 10.1016/j.exger.2020.110962. Epub 2020 Apr 30. PMID: 32360985.

**D12. Antipsychotics (i.e. other than clozapine or quetiapine) in those with parkinsonism or Dementia with Lewy Bodies (risk of severe extra-pyramidal symptoms).**

D12 (i): Mena MA, de Yébenes JG. Drug-induced parkinsonism. Expert Opin Drug Saf 2006; 5(6):759-71. Review. PubMed PMID: 17044803.

D12 (ii): Eng ML, Welty TE. Management of hallucinations and psychosis in Parkinson's disease. Am J Geriatr Pharmacother 2010; 8(4):316-30. Review. PubMed PMID:20869621.

**D13. Anticholinergics/antimuscarinic drugs (biperiden, ophenadrine, procyclidine, trihexyphenidyl) to treat extra-pyramidal side-effects of antipsychotic medications (risk of anticholinergic toxicity).**

D13 (i): Heinik J. Effects of trihexyphenidyl on MMSE and CAMCOG scores of medicated elderly patients with schizophrenia. Int Psychogeriatr 1998; 10(1): 103-8. PubMed PMID: 9629529.

D13 (ii): Drimer T, Shahal B, Barak Y. Effects of discontinuation of long-term anticholinergic treatment in elderly schizophrenia patients. Int Clin Psychopharmacol 2004; 19(1):27-9. PubMed PMID: 15101567.

**D14. Drugs with potent anticholinergics/antimuscarinic effects in patients with delirium or dementia (risk of exacerbation of cognitive impairment).**

** Commonly prescribed drugs with potent anticholinergic/ antimuscarinic effects include tricyclic antidepressants (e.g., amitriptyline, doxepin, impramine, nortriptyline), certain antipsychotics (chlorpromazine, clozapine, thioridazine), first generation antihistamines (e.g. diphenhydramine, chlorpheniramine), certain bladder anti-spasmodics (e.g., tolterodine, oxybutynin), hyoscine, procyclidine, benzatropine, tizanidine.

D14 (i): Pagoria D, O'Connor RC, Guralnick ML. Antimuscarinic drugs: review of the cognitive impact when used to treat overactive bladder in elderly patients. Curr Urol Rep 2011; 12 (5): 351-7. Review. PubMed PMID: 21607875.

D14 (ii): Gerretsen P, Pollock BG. Drugs with anticholinergic properties: a current perspective on use and safety. Expert Opin Drug Saf 2011; 10(5): 751-65. Review. PubMed PMID: 21635190.

**D15. Neuroleptic antipsychotics in patients with non-cognitive symptoms of dementia (NCSD) for longer than 12 weeks unless symptoms are severe and other treatments have failed (increased risk of stroke, myocardial infarction).**

D15 (i): Sacchetti E, Turrina C, Valsecchi P. Cerebrovascular accidents in elderly people treated with antipsychotic drugs: a systematic review. Drug Saf 2010; 33(4): 273-88. Review. PubMed PMID: 20297860.

D15 (ii): Mittal V, Kurup L, Williamson D, Muralee S, Tampi RR. Risk of cerebrovascular adverse events and death in elderly patients with dementia when treated with antipsychotic medications: a literature review of evidence. Am J Alzheimers Dis Other Demen 2011; 26(1): 10-28. Review. PubMed PMID: 21282274.

D15 (iii): Chahine LM, Acar D, Chemali Z. The elderly safety imperative and antipsychotic usage. Harvard Rev Psychiatr 2010; 18:3 158-172. PubMed PMID: 20415632.

**D16. Neuroleptic antipsychotics as hypnotics, unless sleep disorder is due to psychosis or non-cognitive symptoms of dementia (risk of confusion, hypotension, extra-pyramidal side effects, falls).**

D16 (i): RD McEvoy, KS Nyfort-Hansen. Sleep disorders in the elderly: the pros and cons of prescribing. In: Prescribing for Elderly Patients, eds. S. Jackson, P. Jansen, A. Mangoni. Wiley-Blackwell 2009, pp 45-52.

D16 (ii): Alexopoulos GS, Streim J, Carpenter D, Docherty JP. Expert Consensus Panel for Using Antipsychotic Drugs in Older Patients. Using antipsychotic agents in older patients. J Clin Psychiatry 2004; 65 Suppl 2:5-99; discussion 100-102; quiz 103-4. Review. PubMed PMID: 14994733.

**D17. Acetylcholinesterase inhibitors with a known history of persistent bradycardia (< 60 beats/min.), heart block or recurrent unexplained syncope (risk of cardiac conduction failure, syncope and injury).**

D17 (i): Salarbaks AM, Boomkamp-Snoeren CM, van Puijenbroek E, Jansen PA, van Marum RJ. [Cardiac effects of cholinesterase inhibitors: a reason for restraint?]. Tijdschr Gerontol Geriatr 2009; 40(2):79-84. PubMed PMID: 19472574.

D17 (ii): [Fisher A.A.](http://0-www.embase.com.library.ucc.ie/search/results) and [Davis M.W.](http://0-www.embase.com.library.ucc.ie/search/results) Prolonged QT interval, syncope, and delirium with galantamine Ann Pharmacother 2008 42; 2: 278-283. PubMed PMID: 18182475.

D17 (iii): [Suleyman T.](http://0-www.embase.com.library.ucc.ie/search/results), [Tevfik P.](http://0-www.embase.com.library.ucc.ie/search/results), [Abdulkadir G.](http://0-www.embase.com.library.ucc.ie/search/results) and [Ozlem S.](http://0-www.embase.com.library.ucc.ie/search/results) Complete atrioventricular block and ventricular tachyarrhythmia associated with donepezil**.** Emerg Med J 2006; 23(8): 641-2. PubMed PMID: 16858101.

D17 (iv): [Bordier P.](http://0-www.embase.com.library.ucc.ie/search/results), [Lanusse S.](http://0-www.embase.com.library.ucc.ie/search/results), [Garrigue S.](http://0-www.embase.com.library.ucc.ie/search/results), [Reynard C.](http://0-www.embase.com.library.ucc.ie/search/results), [Robert F.](http://0-www.embase.com.library.ucc.ie/search/results), [Gencel L.](http://0-www.embase.com.library.ucc.ie/search/results) and [Lafitte A.](http://0-www.embase.com.library.ucc.ie/search/results) Causes of syncope in patients with Alzheimer's disease treated with donepezil. Drugs Aging  2005;  22(8): 687-694. PubMed PMID: 16060718.

**D18. Acetylcholinesterase inhibitors with concurrent treatment with drugs that reduce heart rate such as beta-blockers, digoxin, diltiazem, verapamil (risk of cardiac conduction failure, syncope and injury).**

D18 (i): Salarbaks AM, Boomkamp-Snoeren CM, van Puijenbroek E, Jansen PA, van Marum RJ. [Cardiac effects of cholinesterase inhibitors: a reason for restraint?]. Tijdschr Gerontol Geriatr 2009; 40(2):79-84. PubMed PMID: 19472574.

D18 (ii): [Fisher A.A.](http://0-www.embase.com.library.ucc.ie/search/results) and [Davis M.W.](http://0-www.embase.com.library.ucc.ie/search/results) Prolonged QT interval, syncope, and delirium with galantamine Ann Pharmacother 2008 42; 2: 278-283. PubMed PMID: 18182475.

D18 (iii): [Suleyman T.](http://0-www.embase.com.library.ucc.ie/search/results), [Tevfik P.](http://0-www.embase.com.library.ucc.ie/search/results), [Abdulkadir G.](http://0-www.embase.com.library.ucc.ie/search/results) and [Ozlem S.](http://0-www.embase.com.library.ucc.ie/search/results) Complete atrioventricular block and ventricular tachyarrhythmia associated with donepezil**.** Emerg Med J 2006; 23(8): 641-2. PubMed PMID: 16858101.

D18 (iv): [Bordier P.](http://0-www.embase.com.library.ucc.ie/search/results), [Lanusse S.](http://0-www.embase.com.library.ucc.ie/search/results), [Garrigue S.](http://0-www.embase.com.library.ucc.ie/search/results), [Reynard C.](http://0-www.embase.com.library.ucc.ie/search/results), [Robert F.](http://0-www.embase.com.library.ucc.ie/search/results), [Gencel L.](http://0-www.embase.com.library.ucc.ie/search/results) and [Lafitte A.](http://0-www.embase.com.library.ucc.ie/search/results) Causes of syncope in patients with Alzheimer's disease treated with donepezil. Drugs Aging  2005;  22(8): 687-694. PubMed PMID: 16060718.

**D19. Memantine with known current or previous seizure disorder (increased risk of seizures).**

D19 (i): British National Formulary, No. 81, March - September 2021, BMJ Group & Pharmaceutical Press, p320.

**D20. Nootropics in dementia including Gingko Biloba, piracetam, pramiracetam, phenylpiracetam, aniracetam, phosphatidylserine, modafinil, L-theanine, omega-3 fatty acids, panax ginseng, rhodiola, creatine (no evidence of efficacy).**

D20 (i): Solomon PR, Adams F, Silver A, Zimmer J, DeVeaux R. Ginkgo for memory enhancement: a randomized controlled trial. JAMA. 2002 Aug 21;288(7):835-40. doi: 10.1001/jama.288.7.835. PMID: 12186600.

D20 (ii): Flicker L, Grimley Evans J. Piracetam for dementia or cognitive impairment. Cochrane Database Syst Rev. 2000;(2):CD001011. doi: 10.1002/14651858.CD001011. Update in: Cochrane Database Syst Rev. 2001;(2):CD001011. PMID: 10796585.

D20 (iii): Quinn JF, Raman R, Thomas RG, Yurko-Mauro K, Nelson EB, Van Dyck C, Galvin JE, Emond J, Jack CR Jr, Weiner M, Shinto L, Aisen PS. Docosahexaenoic acid supplementation and cognitive decline in Alzheimer disease: a randomized trial. JAMA. 2010 Nov 3;304(17):1903-11. doi: 10.1001/jama.2010.1510. PMID: 21045096.

**D21. Phenothiazines as first-line treatment for psychosis or non-cognitive symptoms of dementia (NCSD) since safer and more efficacious alternatives exist (phenothiazines are sedative, have significant anti-muscarinic toxicity in older people, with the exception of prochlorperazine for nausea/vomiting/vertigo, chlorpromazine for relief of persistent hiccoughs and levomepromazine as an anti-emetic in palliative care).**

D21 (i): Chahine L.M., Acar D., Chemali Z. [The elderly safety imperative and antipsychotic usage](http://0-www.embase.com.library.ucc.ie/search/results?subaction=viewrecord&id=L358740034&from=clipboard&L358740034). Harvard Review of Psychiatry 2010; 18(3): 158-72. PubMed PMID: 20415632.

D21 (ii): Love R.C. [The differential pharmacology of atypical antipsychotics: Impact on patients with comorbid conditions](http://0-www.embase.com.library.ucc.ie/search/results?subaction=viewrecord&id=L44956798&from=clipboard&L44956798). Consult Pharm 2006; 21; SUPPL. B: 11-18.

**D22. Levodopa or dopamine agonists for benign essential tremor (no evidence of efficacy)**

D22 (i): Zesiewicz TA, Elble RJ, Louis ED, Gronseth GS, Ondo WG, Dewey RB Jr, Okun MS, Sullivan KL, Weiner WJ. Evidence-based guideline update- treatment of essential tremor-report of the Quality Standards subcommittee of the American Academy of Neurology. Neurology 2011; 77(19):1752-5. Review. PubMed PMID: 22013182.

D22 (ii): Deuschl G, Raethjen J, Hellriegel H, Elble R. Treatment of patients with essential tremor. Lancet Neurol 2011; 10(2): 148-61. Review. PubMed PMID:21256454.

**D23. Levodopa or dopamine agonists for treatment of extrapyramidal side-effects of antipsychotics or other forms of drug-induced Parkinsonism (inappropriate prescribing cascade to be avoided).**

D23 (i): Hardie RJ, Lees AJ. Neuroleptic-induced Parkinson's syndrome: clinical features and results of treatment with levodopa. J Neurol Neurosurg Psychiatry. 1988;51:850–854.

D23 (ii): Hassin-Baer S, Sirota P, Korczyn AD, Treves TA, Epstein B, Shabtai H, et al. Clinical characteristics of neuroleptic-induced parkinsonism. J Neural Transm. 2001;108:1299–1308.

D23 (iii): Shin HW, Chung SJ. Drug-induced parkinsonism. J Clin Neurol. 2012 Mar;8(1):15-21. doi: 10.3988/jcn.2012.8.1.15. Epub 2012 Mar 31. PMID: 22523509.

**D24. First-generation antihistamines as first line treatment for allergy or pruritis (safer, less toxic antihistamines with fewer side effects now widely available).**

D24 (i): Hansen J, Klimek L, Hörmann K. Pharmacological management of allergic rhinitis in the elderly-safety issues with oral antihistamines. Drugs Aging 2005; 22(4): 289-96. Review. PubMed PMID: 15839718.

D24 (ii): Slavin RG. Special considerations in treatment of allergic rhinitis in the elderly: role of intranasal corticosteroids. Allergy Asthma Proc 2010; 31(3): 179-84. Review. PubMed PMID: 20615319.

D24 (iii): Church MK, Maurer M, Simons FE, Bindslev-Jensen C, van Cauwenberge P, Bousquet J, Holgate ST, Zuberbier T; Global Allergy and Asthma European Network. Risk of first-generation H(1)-antihistamines: a GA(2)LEN position paper. Allergy. 2010 Apr;65(4):459-66. doi: 10.1111/j.1398-9995.2009.02325.x. Epub 2010 Feb 8. PMID: 20146728.

**D25. First-generation antihistamines for insomnia (high risk of side-effects, Z-drugs safer and more appropriate for short-term use).**

D25 (i): National Institutes of Health. National Institutes of Health State of the Science Conference statement on Manifestations and Management of Chronic Insomnia in Adults, June 13-15, 2005. Sleep. 2005 Sep;28(9):1049-57. doi: 10.1093/sleep/28.9.1049. PMID: 16268373.

**Section E. Renal System criteria.**

**E1. Digoxin at a long-term (i.e. more than 90 days) maintenance dose ≥ 125µg/day if eGFR < 30 ml/min/1.73m^2^ (risk of digoxin toxicity if plasma levels not measured).**

E1 (i) Ziff OJ, Lane DA, Samra M, Griffith M, Kirchhof P, Lip GY, Steeds RP, Townend J, Kotecha D. Safety and efficacy of digoxin: systematic review and meta-analysis of observational and controlled trial data. BMJ 2015;351:h4451.

E (ii): Ahmed A. Digoxin and reduction in mortality and hospitalization in geriatric heart failure: importance of low doses and low serum concentrations. J Gerontol A Biol Sci Med Sci. 2007; 62(3): 323-9. PubMed PMID: 17389731.

E1 (iii): Podrazik PM, Schwartz JB. Cardiovascular pharmacology of aging. Cardiol Clin 1999; 17(1): 17-34. Review. PubMed PMID: 10093763.

**E2. Direct thrombin inhibitors (e.g. dabigatran) if eGFR < 30 ml/min/1.73m^2^ (risk of bleeding)**

E2 (i): Hariharan S, Madabushi R. Clinical pharmacology basis of deriving dosing recommendations for dabigatran in patients with severe renal impairment. J ClinPharmacol 2012; 52(1 Suppl):119S-25S. PubMed PMID: 21956605.

E2 (ii): Samama MM. Use of low-molecular-weight heparins and new anticoagulants in elderly patients with renal impairment. Drugs Aging 2011; 28(3): 177-93.PubMed PMID: 21329400.

**E3. Factor Xa inhibitors (e.g. rivaroxaban, apixaban) if eGFR < 15 ml/min/1.73m^2^ (risk of bleeding)**

E3 (i): Kubitza D, Becka M, Mueck W, Halabi A, Maatouk H, Klause N, Lufft V, Wand DD, Philipp T, Bruck H. Effects of renal impairment on the pharmacokinetics, pharmacodynamics and safety of rivaroxaban, an oral, direct Factor Xa inhibitor. Br J Clin Pharmacol 2010; 70(5): 703-12.

PubMed PMID: 21039764.

E3 (ii): Fox KA, Piccini JP, Wojdyla D, Becker RC, Halperin JL, Nessel CC, Paolini JF, Hankey GJ, Mahaffey KW, Patel MR, Singer DE, Califf RM. Prevention of stroke and systemic embolism with rivaroxaban compared with warfarin in patients withnon-valvular atrial fibrillation and moderate renal impairment. Eur Heart J 2011; 32(19): 2387-94. PubMed PMID: 21873708.

**E4. NSAID’s if eGFR < 50 ml/min/1.73m^2^ (risk of deterioration in renal function).**

E4 (i): Harirforoosh S, Jamali F. Renal adverse effects of non-steroidal anti-inflammatory drugs. Expert Opin Drug Saf 2009; 8(6): 669-81. Review. PubMed PMID: 19832117.

E4 (ii): Cheng HF, Harris RC. Renal effects of non-steroidal anti-inflammatory drugs and selective cyclooxygenase-2 inhibitors. Curr Pharm Des 2005; 11(14): 1795-804. Review. PubMed PMID: 15892676.

**E5. Colchicine if eGFR < 10 ml/min/1.73m2 (risk of colchicine toxicity).**

E5 (i): Hoskison KT, Wortmann RL. Management of gout in older adults: barriers to optimal control. Drugs Aging 2007; 24(1): 21-36. Review. PubMed PMID: 17233545.

E5 (ii): Hanlon JT, Aspinall SL, Semla TP, Weisbord SD, Fried LF, Good CB, Fine MJ, Stone RA, Pugh MJ, Rossi MI, Handler SM. Consensus guidelines for oral dosing of primarily renally cleared medications in older adults. J Am Geriatr Soc 2009; 57(2):335-40. Erratum in: J Am Geriatr Soc 2009; 57(11): 2179. Dosage error in article text. PubMed PMID: 19170784.

**E6. Metformin if eGFR < 30 ml/min/1.73m^2^ (risk of lactic acidosis).**

E6 (i): Germino FW. Non-insulin treatment of type 2 diabetes mellitus in geriatric patients: a review. Clin Ther 2011; 33(12): 1868-82. Review.PubMed PMID: 22136979.

E6 (ii): [Lalau JD.](http://0-www.embase.com.library.ucc.ie/search/results) Lactic acidosis induced by metformin: Incidence, management and prevention. Drug Safety 2010; 33(9): 727-40. PubMed PMID: 20701406.

**E7. Mineralocorticoid receptor antagonists (e.g. spironolactone, eplerenone) if eGFR < 30 ml/min/1.73m^2^ (risk of dangerous hyperkalaemia).**

E7 (i): Currie, G., Taylor, A.H.M., Fujita, T. *et al.* Effect of mineralocorticoid receptor antagonists on proteinuria and progression of chronic kidney disease: a systematic review and meta-analysis. *BMC Nephrol* 17, 127 (2016). <https://doi.org/10.1186/s12882-016-0337-0>.

E7 (ii): Juurlink DN, Mamdani MM, Lee DS, Kopp A, Austin PC, Laupacis A, Redelmeier DA. Rates of hyperkalemia after publication of the Randomized Aldactone Evaluation Study. N Engl J Med. 2004 Aug 5;351(6):543-51. doi: 10.1056/NEJMoa040135. PMID: 15295047.

E7 (iii): Lainscak M, Pelliccia F, Rosano G, Vitale C, Schiariti M, Greco C, Speziale G, Gaudio C. Safety profile of mineralocorticoid receptor antagonists: Spironolactone and eplerenone. Int J Cardiol. 2015 Dec 1;200:25-9. doi: 10.1016/j.ijcard.2015.05.127. Epub 2015 May 21. PMID: 26404748.

E7 (iv): Khan MS, Khan MS, Moustafa A, Anderson AS, Mehta R, Khan SS. Efficacy and Safety of Mineralocorticoid Receptor Antagonists in Patients With Heart Failure and Chronic Kidney Disease. Am J Cardiol. 2020 Feb 15;125(4):643-650. doi: 10.1016/j.amjcard.2019.11.014. Epub 2019 Nov 19. PMID: 31843235; PMCID: PMC7977068.

**E8. Nitrofurantoin if eGFR < 45 ml/min/1.73m^2^ (increased risk of nitrofurantoin toxicity).**

E8 (i): Sachs J, Geer T, Noell P, et al. Effect of renal function on urinary recovery of orally administered nitrofurantoin. N Engl J Med 1968; 278:1032–5.

E8 (ii): British National Formulary, No. 81, March – September, BMJ Group & Pharmaceutical Press, 2021, p629.

**E9. Bisphosphonates if eGFR<30 ml/min/1.73m^2^ (increased risk of acute renal failure).**

E9 (i): Miller PD, Jamal SA, Evenepoel P, Eastell R, Boonen S. Renal safety in patients treated with bisphosphonates for osteoporosis: a review. J Bone Miner Res. 2013 Oct;28(10):2049-59. doi: 10.1002/jbmr.2058. PMID: 23907861.

E9 (ii): British National Formulary, No. 81, March – September, BMJ Group & Pharmaceutical Press 2021, p768.

**E10. Methotrexate if eGFR <30 ml/min/1.73m^2^.**

E10 (i): Saag K.G., Teng G.G., Patkar N.M., Anuntiyo J., Finney C., Curtis J.R., Paulus H.E., Mudano A., Pisu M., Elkins-Melton M., et al. American College of Rheumatology 2008 recommendations for the use of nonbiologic and biologic disease-modifying antirheumatic drugs in rheumatoid arthritis. *Arthritis Rheum.*2008;59:762–784. doi: 10.1002/art.23721.

E10 (ii): British National Formulary, No. 81, March – September, BMJ Group & Pharmaceutical Press, 2021, p958 .

**Section F: Gastrointestinal System criteria.**

**F1. Prochlorperazine or metoclopramide with Parkinsonism (risk of exacerbating Parkinsonian symptoms).**

F1 (i): Stephen PJ, Williamson J. Drug-induced parkinsonism in the elderly. Lancet 1984; 2(8411): 1082-3. PubMed PMID: 6150149.

F1 (ii): Ganzini L, Casey DE, Hoffman WF, McCall AL. The prevalence of metoclopramide-induced tardive dyskinesia and acute extrapyramidal movement disorders. Arch Intern Med 1993; 153(12): 1469-75. PubMed PMID: 8512437.

F1 (iii): Pasricha PJ, Pehlivanov N, Sugumar A, Jankovic J. Drug Insight: from disturbed motility to disordered movement - a review of the clinical benefits and medicolegal risks of metoclopramide. Nat Clin Pract Gastroenterol Hepatol 2006; 3(3): 138-48. Review. PubMed PMID: 16511548.

**F2. PPI for uncomplicated peptic ulcer disease or erosive peptic oesophagitis at full therapeutic dosage for > 8 weeks (dose reduction or earlier discontinuation or H2 antagonist maintenance usually indicated).**

F2 (i): British National Formulary No. 81, March – September 2021, BMJ Group & Pharmaceutical Press, pp 83-88.

F2 (ii): NICE guideline 2000/022 (last updated 14 July 2008). [www.nice.org.uk](http://www.nice.org.uk).

**F3. Drugs likely to cause constipation (e.g. antimuscarinic/anticholinergic drugs, oral iron, opioids, verapamil, aluminium antacids) in patients with chronic constipation where non-constipating alternatives are appropriate (risk of exacerbation of constipation).**

F3 (i): Meek PD, Evang SD, Tadrous M, Roux-Lirange D, Triller DM, Gumustop B. Overactive bladder drugs and constipation: a meta-analysis of randomized, placebo-controlled trials. Dig Dis Sci 2011; 56(1): 7-18. Review. PubMed PMID: 20596778.

F3 (ii): Müller-Lissner S. General geriatrics and gastroenterology: constipation and faecal incontinence. Best Pract Res Clin Gastroenterol 2002; 16(1): 115-33. Review. PubMed PMID: 11977932.

F3 (iii): Harari D, Gurwitz JH, Avorn J, Choodnovskiy I, Minaker KL. Correlates of regular laxative use by frail elderly persons. Am J Med 1995; 99(5): 513-8. PubMed PMID: 7485209.

F3 (iv): Opie LH. Choosing the correct drug for the individual hypertensive patient. Drugs 1992; 44 Suppl 1: 147-55. Review. PubMed PMID: 1283579.

**F4. Oral elemental iron doses greater than 200 mg daily (e.g. ferrous fumarate > 600 mg/day, ferrous sulphate > 600 mg/day, ferrous gluconate > 1800 mg/day; no evidence of enhanced iron absorption above these doses).**

F4 (i): Rimon E, Kagansky N, Kagansky M, Mechnick L, Mashiah T, Namir M, Levy S. Are we giving too much iron? Low-dose iron therapy is effective in octogenarians. Am J Med 2005; 118(10): 1142-7. PubMed PMID: 16194646.

F4 (ii): British National Formulary No. 81, March - September, 2021, BMJ Group & Pharmaceutical Press, pp1065-71.

**F5. Corticosteroids with a history of peptic ulcer disease or erosive oesophagitis (risk of relapse unless proton pump inhibitor is co-prescribed).**

F5 (i): Ellershaw JE, Kelly MJ. Corticosteroids and peptic ulceration. Palliat Med. 1994 Oct;8(4):313-9. doi: 10.1177/026921639400800407. PMID: 7812482.

F5 (ii): Conn HO, Poynard T. Corticosteroids and peptic ulcer: meta-analysis of adverse events during steroid therapy. J Intern Med. 1994 Dec;236(6):619-32. doi: 10.1111/j.1365-2796.1994.tb00855.x. PMID: 7989897.

**F6. Antiplatelet or anticoagulant drugs with a history of Gastric Antral Vascular Ectasia (GAVE, “watermelon stomach”) (risk of major gastrointestinal bleeding).**

F6 (i): Eccles J, Falk V, Montano-Loza AJ, Zepeda-Gómez S. Long-term follow-up in patients with gastric antral vascular ectasia (GAVE) after treatment with endoscopic band ligation (EBL). Endosc Int Open. 2019 Dec;7(12):E1624-E1629. doi: 10.1055/a-0977-2870. Epub 2019 Nov 25. PMID: 31788543; PMCID: PMC6877425.

F6 (ii): Boltin D, Gingold-Belfer R, Lichtenstein L et al. Long-term treatment outcome of patients with gastric antral vascular ectasia treated with argon plasma coagulation. *Eur J Gastroenterol Hepatol.*2014;26:588–593.

**F7. Antipsychotics with dysphagia (increased risk of aspiration pneumonia).**

F7 (i): van der Maarel-Wierink CD, Vanobbergen JN, Bronkhorst EM, Schols JM, de Baat C. Risk factors for aspiration pneumonia in frail older people: a systematic literature review. J Am Med Dir Assoc. 2011 Jun;12(5):344-54. doi: 10.1016/j.jamda.2010.12.099. Epub 2011 Mar 21. PMID: 21450240.

F7 (ii): Trifirò G. Antipsychotic drug use and community-acquired pneumonia. Curr Infect Dis Rep. 2011 Jun;13(3):262-8. doi: 10.1007/s11908-011-0175-y. PMID: 21394430; PMCID: PMC3085099.

F7 (iii): Nosè M, Recla E, Trifirò G, Barbui C. Antipsychotic drug exposure and risk of pneumonia: a systematic review and meta-analysis of observational studies. Pharmacoepidemiol Drug Saf. 2015 Aug;24(8):812-20. doi: 10.1002/pds.3804. Epub 2015 May 27. PMID: 26017021.

F7 (iv): Miarons Font M, Rofes Salsench L. Antipsychotic medication and oropharyngeal dysphagia: systematic review. Eur J Gastroenterol Hepatol. 2017 Dec;29(12):1332-1339. doi: 10.1097/MEG.0000000000000983. PMID: 29023321.

#### F8. Megestrol acetate to increase appetite (increased risk of thrombosis and death with unproven efficacy).

F8 (i): Bolen JC, Andersen RE, Bennett RG. Deep vein thrombosis as a complication of megestrol acetate therapy among nursing home residents. J Am Med Dir Assoc. 2000 Nov-Dec;1(6):248-52. PMID: 12812608.

F8 (ii): Wen FK, Millar J, Oberst-Walsh L, Nashelsky J. Clinical Inquiry: Is megestrol acetate safe and effective for malnourished nursing home residents? J Fam Pract. 2018 Feb;67(2):112-113. PMID: 29400904

**Section G. Respiratory System criteria.**

**G1. Theophylline as monotherapy for COPD (safer, more effective alternative; risk of adverse effects due to narrow therapeutic index).**

G1 (i): Rabe KF, Hurd S, Anzueto A, Barnes PJ, Buist SA, Calverley P, Fukuchi Y, Jenkins C, Rodriguez-Roisin R, van Weel C, Zielinski J; Global Initiative for Chronic Obstructive Lung Disease. Global strategy for the diagnosis, management, and prevention of chronic obstructive pulmonary disease: GOLD executive summary. Am J Respir Crit Care Med 2007; 176(6): 532-55. Review. PubMed PMID: 17507545.

G1 (ii): Ramsdell J. Use of theophylline in the treatment of COPD. Chest 1995; 107(5 Suppl): 206S-209S. Review. PubMed PMID: 7743828.

**G2. Systemic corticosteroids instead of inhaled corticosteroids for maintenance therapy in moderate-severe COPD (unnecessary exposure to long-term side-effects of systemic corticosteroids and effective inhaled therapies are available).**

G2 (i): Rabe KF, Hurd S, Anzueto A, Barnes PJ, Buist SA, Calverley P, Fukuchi Y, Jenkins C, Rodriguez-Roisin R, van Weel C, Zielinski J; Global Initiative for Chronic Obstructive Lung Disease. Global strategy for the diagnosis, management, and prevention of chronic obstructive pulmonary disease: GOLD executive summary. Am J Respir Crit Care Med 2007; 176(6): 532-55. Review. PubMed PMID: 17507545.

G2 (ii): Wood-Baker R, Walters J, Walters EH. Systemic corticosteroids in chronic obstructive pulmonary disease: an overview of Cochrane systematic reviews. Respir Med 2007; 101(3): 371-7. Review. PubMed PMID: 16962307.

**G3. Long-acting muscarinic antagonists (e.g. tiotropium, aclidinium, umeclidinium, glycopyrronium) with a history of narrow angle glaucoma (may exacerbate glaucoma) or bladder outflow obstruction (may cause urinary retention).**

G3 (i): Gupta P, O'Mahony MS. Potential adverse effects of bronchodilators in the treatment of airways obstruction in older people: recommendations for prescribing. Drugs Aging 2008; 25(5): 415-43. Review. PubMed PMID: 18447405.

G3 (ii): Oba Y, Zaza T, Thameem DM. Safety, tolerability and risk benefit analysis of tiotropium in COPD. Int J Chron Obstruct Pulmon Dis 2008; 3(4): 575-84. Review. PubMed PMID: 19281075.

**G4. Benzodiazepines with acute or chronic respiratory failure i.e. pO2 < 8.0 kPa ± pCO2 > 6.5 kPa (risk of exacerbation of respiratory failure).**

G4 (i): Model DG, Berry DJ. Effects of chlordiazepoxide in respiratory failure due to chronic bronchitis. Lancet 1974; 2(7885): 869-70. PubMed PMID: 4137638.

G4 (ii): Hak E, Bont J, Hoes AW, Verheij TJ. Prognostic factors for serious morbidity and mortality from community-acquired lower respiratory tract infections among the elderly in primary care. Fam Pract 2005; 22(4): 375-80. PubMed PMID: 15805127.

**Section H: Musculoskeletal System criteria.**

**H1. Non-COX-2 selective non-steroidal anti-inflammatory drug (NSAID) with history of peptic ulcer disease or gastrointestinal bleeding, unless with concurrent PPI or H2 antagonist (risk of peptic ulcer relapse).**

H1 (i): Blandizzi C, Tuccori M, Colucci R, Gori G, Fornai M, Antonioli L, Ghisu N, Del Tacca M. Clinical efficacy of esomeprazole in the prevention and healing of gastrointestinal toxicity associated with NSAIDs in elderly patients. Drugs Aging 2008; 25(3): 197-208. Review. PubMed PMID: 18331072.

H1 (ii): Lanas A, Ferrandez A. Inappropriate prevention of NSAID-induced gastrointestinal events among long-term users in the elderly. Drugs Aging 2007; 24(2):121-31. Review. PubMed PMID: 17313200.

H1(iii): Alaa Rostom, Catherine Dube, George A Wells, Peter Tugwell, Vivian Welch, Emilie Jolicoeur, Jessie McGowan, Angel Lanas. Prevention of NSAID-induced gastroduodenal ulcers. Editorial Group: [Cochrane Upper Gastrointestinal and Pancreatic Diseases Group](http://onlinelibrary.wiley.com/o/cochrane/clabout/articles/UPPERGI/frame.html) Published Online: 20 Jan 2010. DOI: 10.1002/14651858.CD002296.

**H2. NSAID’s with severe hypertension i.e., systolic blood pressure consistently above 170 mmHg and/or diastolic blood pressure consistently above 100 mmHg (risk of exacerbation of hypertension).**

H2 (i): White WB. Defining the problem of treating the patient with hypertension and arthritis pain. Am J Med. 2009; 122(5 Suppl): S3-9. Review. PubMed PMID: 19393824.

H2 (ii): [Park KE.Qin Y](http://0-www.embase.com.library.ucc.ie/search/results), [Bavry AA.](http://0-www.embase.com.library.ucc.ie/search/results) Nonsteroidal anti-inflammatory drugs and their effects in the elderly. Aging Health 2012; 8(2): 167-177.

**H3. Long-term use of NSAID (>3 months) for symptom relief of osteoarthritis pain where paracetamol has not been tried (simple analgesics preferable and usually as effective for pain relief)**

H3 (i): Nikles CJ, Yelland M, Del Mar C, Wilkinson D. The role of paracetamol in chronic pain: an evidence-based approach. Am J Ther 2005; 12(1): 80-91. Review. PubMed PMID: 15662295.

H3 (ii): Seed SM, Dunican KC, Lynch AM. Osteoarthritis: a review of treatment options. Geriatrics 2009; 64(10): 20-9. Review. PubMed PMID: 20726384.

H3 (iii): Jawad AS. Analgesics and osteoarthritis: are treatment guidelines reflected in clinical practice? Am J Ther 2005; 12(1): 98-103. Review. PubMed PMID: 15662297.

**H4. Long-term corticosteroids (>3 months) as monotherapy for rheumatoid arthritis (risk of systemic corticosteroid side-effects).**

H4 (i): Onishi S, Iwmoto M, [Minota S.](http://0-www.embase.com.library.ucc.ie/search/results) Management of elderly-onset rheumatoid arthritis. J Clin Immunol 2010; 33(1): 1-7. PubMed PMID: 20190503.

H4 (ii): American College of Rheumatology Subcommittee on Rheumatoid Arthritis Guidelines. Guidelines for the management of rheumatoid arthritis: 2002 Update. Arthritis Rheum 2002; 46(2): 28-46. PubMed PMID: 11840435.

H4 (iii): Soubrier M, Mathieu S, Payet S, Dubost JJ, Ristori JM. Elderly-onset rheumatoid arthritis. Joint Bone Spine 2010; 77(4): 290-6. Review. PubMed PMID: 20554241.

**H5. Corticosteroids (other than periodic intra-articular injections for mono-articular pain) for osteoarthritis (risk of systemic corticosteroid side-effects).**

H5 (i): British National Formulary No. 81, March – September 2021, p1140.

H5 (ii): Recommendations for the medical management of osteoarthritis of the hip and knee: 2000 update. American College of Rheumatology Subcommittee on Osteoarthritis Guidelines. Arthritis Rheum 2000; 43(9): 1905-15. PubMed PMID: 11014340.

**H6. Long-term NSAID or colchicine (>3 months) for prevention of relapses of gout where there is no contraindication to a xanthine-oxidase inhibitor e.g. allopurinol, febuxostat (xanthine-oxidase inhibitors are first choice prophylactic drugs in gout).**

H6 (i): De Leonardis F, Govoni M, Colina M, Bruschi M, Trotta F. Elderly-onset gout: a review. Rheumatol Int 2007; 28(1): 1-6. Review. PubMed PMID: 17653719.

H6 (ii): Hoskison KT, Wortmann RL. Management of gout in older adults: barriers to optimal control. Drugs Aging 2007; 24(1): 21-36. Review. PubMed PMID: 17233545.

**H7. NSAID with concurrent corticosteroids for treatment of arthritis/rheumatism of any kind (increased risk of peptic ulcer disease).**

H7 (i): Peng S, Duggan A. Gastrointestinal adverse effects of non-steroidal anti-inflammatory drugs. Expert Opin Drug Saf 2005; 4(2): 157-69. Review. PubMed PMID: 15794710.

H7 (ii): Zullo A, Hassan C, Campo SM, Morini S. Bleeding peptic ulcer in the elderly: risk factors and prevention strategies. Drugs Aging 2007; 24(10): 815-28. Review. PubMed PMID: 17896831.

H7 (iii): Scheiman JM. NSAID-induced peptic ulcer disease: a critical review of pathogenesis and management. Dig Dis 1994; 12(4): 210-22. Review. PubMed PMID: 7851000.

**H8. Oral bisphosphonates in patients with a history of upper gastrointestinal disease i.e. dysphagia, oesophagitis, gastritis, duodenitis, or peptic ulcer disease, or upper gastrointestinal bleeding (risk of relapse/exacerbation of oesophagitis, oesophageal ulcer, oesophageal stricture)**

H8 (i): Pazianas M, Abrahamsen B. Safety of bisphosphonates. Bone 2011; 49(1): 103-10. Review.PubMed PMID: 21236370.

H8 (ii): Civitelli R, Napoli N, Armamento-Villareal R. Use of intravenous bisphosphonates in osteoporosis. Curr Osteoporos Rep 2007; 5(1): 8-13. PubMed PMID: 17320022.

H8 (iii): Gaudio A, Morabito N. Pharmacological management of severe postmenopausal

osteoporosis. Drugs Aging 2005; 22(5): 405-17. Review. PubMed PMID: 15903353.

**H9. Long-term opioids for osteoarthritis (lack of evidence of efficacy, increased risk of serious side-effects).**

H9 (i): Welsch P, Petzke F, Klose P, Häuser W. Opioids for chronic osteoarthritis pain: An updated systematic review and meta-analysis of efficacy, tolerability and safety in randomized placebo-controlled studies of at least 4 weeks double-blind duration. Eur J Pain. 2020 Apr;24(4):685-703. doi: 10.1002/ejp.1522. Epub 2020 Jan 17. Erratum in: Eur J Pain. 2020 Aug;24(7):1420. PMID: 31876347.

H9 (ii): Bialas P, Maier C, Klose P, Häuser W. Efficacy and harms of long-term opioid therapy in chronic non-cancer pain: Systematic review and meta-analysis of open-label extension trials with a study duration ≥26 weeks. Eur J Pain. 2020 Feb;24(2):265-278. doi: 10.1002/ejp.1496. Epub 2019 Nov 14. PMID: 31661587.

H9 (iii): da Costa BR, Nüesch E, Kasteler R, Husni E, Welch V, Rutjes AW, Jüni P. Oral or transdermal opioids for osteoarthritis of the knee or hip. Cochrane Database Syst Rev. 2014 Sep 17;(9):CD003115. doi: 10.1002/14651858.CD003115.pub4. PMID: 25229835.

**Section I: Urogenital System criteria.**

**I1. Systemic antimuscarinic drugs in patients with dementia or chronic cognitive impairment (risk of increased confusion, agitation).**

I1 (i): Pagoria D, O'Connor RC, Guralnick ML. Antimuscarinic drugs: review of the cognitive impact when used to treat overactive bladder in elderly patients. Curr Urol Rep 2011; 12(5): 351-7. Review. PubMed PMID: 21607875.

I1 (ii): Kay GG, Abou-Donia MB, Messer WS Jr, Murphy DG, Tsao JW, Ouslander JG. Antimuscarinic drugs for overactive bladder and their potential effects on cognitive function in older patients. J Am Geriatr Soc 2005; 53(12): 2195-201. Review. PubMed PMID: 16398909.

**I2. Systemic antimuscarinic drugs in patients with narrow-angle glaucoma (risk of acute exacerbation of glaucoma).**

I2 (i): Lazenby GW, Reed JW, Grant WM. Anticholinergic Medication in Open-Angle Glaucoma: Long-Term Tests. Arch Ophthalmol. 1970;84(6):719–723. doi:10.1001/archopht.1970.00990040721003

I2 (ii): British National Formulary, No. 81, March – September 2021, BMJ Group & Pharmaceutical Press, pp1223-24.

**I3. Systemic antimuscarinic drugs with lower urinary tract symptoms associated with benign prostatic hyperplasia and high post-void residual volume i.e. > 200 ml (uncertain efficacy and increased risk of urinary retention in older men).**

I3 (i): Wolff DT, Adler KA, Weinstein CS, Weiss JP. Managing Nocturia in Frail Older Adults. Drugs Aging. 2021 Feb;38(2):95-109. Review. doi: 10.1007/s40266-020-00815-5. Epub 2020 Nov 24. PMID: 33230803.

I3 (ii): Wang X, Wang X, Li S, Meng Z, Liu T, Zhang X. Comparative effectiveness of oral drug therapies for lower urinary tract symptoms due to benign prostatic hyperplasia: a systematic review and network meta-analysis. PLoS One. 2014 Sep 12;9(9):e107593. doi: 10.1371/journal.pone.0107593. PMID: 25216271; PMCID: PMC4162615.

I3(iii): Chapple C. Antimuscarinics in men with lower urinary tract symptoms suggestive of bladder outlet obstruction due to benign prostatic hyperplasia. Curr Opin Urol. 2010 Jan;20(1):43-8. doi: 10.1097/MOU.0b013e3283330862. PMID: 19875964.

**I4. Systemic antimuscarinic drugs with constipation (risk of exacerbation of constipation).**

I4 (i): Usmani SA, Reckenberg K, Johnson O, Stranges PM, Teshome BF, Kebodeaux CD, Vouri SM. Relative Risk of Adverse Events and Treatment Discontinuations Between Older and Non-Older Adults Treated with Antimuscarinics for Overactive Bladder: A Systematic Review and Meta-Analysis. Drugs Aging. 2019 Jul;36(7):639-645. doi: 10.1007/s40266-019-00674-9. PMID: 31054113.

**I5. Alpha-1 receptor antagonists other than silodosin (e.g. alfuzosin, doxazosin, indoramin, tamsulosin, terazosin) with symptomatic orthostatic hypotension or history of syncope (risk of precipitating recurrent syncope).**

I5 (i):Lowe FC. Role of the newer alpha, -adrenergic-receptor antagonists in the treatment of benign prostatic hyperplasia-related lower urinary tract symptoms. Clin Ther 2004; 26(11): 1701-13. Review. PubMed PMID: 15639685.

I5 (ii): Yoshida M, Kudoh J, Homma Y, Kawabe K. Safety and efficacy of silodosin for the treatment of benign prostatic hyperplasia. Clin Interv Aging. 2011;6:161-72. Review. doi: 10.2147/CIA.S13803. Epub 2011 Jun 22. PMID: 21753871; PMCID: PMC3131986.

**I6. Mirabegron in labile or severe hypertension (risk of exacerbation of hypertension).**

I6 (i): Sacco E, Bientinesi R. Mirabegron: a review of recent data and its prospects in the management of overactive bladder. Ther Adv Urol. 2012 Dec;4(6):315-24. doi: 10.1177/1756287212457114. PMID: 23205058; PMCID: PMC3491758.

I6 (ii): Nitti VW, Khullar V, van Kerrebroeck P, Herschorn S, Cambronero J, Angulo JC, Blauwet MB, Dorrepaal C, Siddiqui E, Martin NE. Mirabegron for the treatment of overactive bladder: a prespecified pooled efficacy analysis and pooled safety analysis of three randomised, double-blind, placebo-controlled, phase III studies. Int J Clin Pract. 2013 Jul;67(7):619-32. doi: 10.1111/ijcp.12194. Epub 2013 May 21. PMID: 23692526; PMCID: PMC3752932.

**I7: Duloxetine with urinary urgency or urge incontinence (duloxetine is indicated in stress incontinence but not in urinary urgency or urge incontinence).**

I7 (i): van Kerrebroeck P, Abrams P, Lange R, Slack M, Wyndaele JJ, Yalcin I, Bump RC; Duloxetine Urinary Incontinence Study Group. Duloxetine versus placebo in the treatment of European and Canadian women with stress urinary incontinence. BJOG. 2004 Mar;111(3):249-57. doi: 10.1111/j.1471-0528.2004.00067.x. PMID: 14961887.

I7 (ii): Norton PA, Zinner NR, Yalcin I, Bump RC; Duloxetine Urinary Incontinence Study Group. Duloxetine versus placebo in the treatment of stress urinary incontinence. Am J Obstet Gynecol. 2002 Jul;187(1):40-8. doi: 10.1067/mob.2002.124840. PMID: 12114886.

**I8. Antibiotic use in asymptomatic bacteriuria (no indication for treatment).**

I8 (i): Cortes-Penfield NW, Trautner BW, Jump RLP. Urinary Tract Infection and Asymptomatic Bacteriuria in Older Adults. Infect Dis Clin North Am. 2017 Dec;31(4):673-688. doi: 10.1016/j.idc.2017.07.002. PMID: 29079155; PMCID: PMC5802407.

I8 (ii): Nicolle LE, Bradley S, Colgan R, et al. Infectious Diseases Society of America guidelines for the diagnosis and treatment of asymptomatic bacteriuria in adults. *Clin Infect Dis.*2005;40(5):643–54.

**Section J: Endocrine System criteria.**

**J1. Sulphonylureas with a half-life (e.g. glibenclamide, chlorpropamide, glimepiride) with type 2 diabetes mellitus (risk of prolonged hypoglycaemia).**

J1(i): Chan SP, Colagiuri S. Systematic review and meta-analysis of the efficacy and hypoglycemic safety of gliclazide versus other insulinotropic agents. Diabetes Res Clin Pract. 2015 Oct;110(1):75-81. doi: 10.1016/j.diabres.2015.07.002. Epub 2015 Jul 9. PMID: 26361859

J1 (ii): Graal MB, Wolffenbuttel BH. The use of sulphonylureas in the elderly. Drugs Aging 1999; 15(6): 471-81. Review. PubMed PMID: 10641958.

J1(iii): Langtry HD, Balfour JA. Glimepiride. A review of its use in the management of type 2 diabetes mellitus. Drugs 1998; 55(4): 563-84. Review. PubMed PMID: 9561345.

**J2. Thiazolidenediones (e.g. rosiglitazone, pioglitazone) in patients with heart failure (risk of exacerbation of heart failure).**

J2(i): Germino FW. Noninsulin treatment of type 2 diabetes mellitus in geriatric patients: a review. Clin Ther 2011; 33(12): 1868-82. Review. PubMed PMID: 22136979.

J2(ii): Lago RM, Singh PP, Nesto RW. Congestive heart failure and cardiovascular death in patients with prediabetes and type 2 diabetes given thiazolidinediones: a meta-analysis of randomised clinical trials. Lancet 2007; 370(9593): 1129-36. Review. PubMed PMID: 17905165.

**J3. Non-selective beta-blockers in diabetes mellitus with frequent hypoglycaemic episodes (risk of suppressing hypoglycaemic symptoms).**

J3 (i): Chelliah A, Burge MR. Hypoglycaemia in elderly patients with diabetes mellitus: causes and strategies for prevention. Drugs Aging 2004; 21(8): 511-30. Review. PubMed PMID: 15182216.

J3 (ii): Sanon VP, Sanon S, Kanakia R, Yu H, Araj F, Oliveros R, Chilton R. Hypoglycemia from a cardiologist's perspective. Clin Cardiol. 2014 Aug;37(8):499-504. doi: 10.1002/clc.22288. Epub 2014 Jun 4. PMID: 24895268; PMCID: PMC6649367.

**J4. Sodium glucose co-transporter (SGLT2) inhibitors (e.g. canagliflozin, dapagliflozin, empagliflozin, ertugliflozin) with symptomatic hypotension (risk of exacerbation of hypotension).**

J4 (i): Baker WL, Smyth LR, Riche DM, Bourret EM, Chamberlin KW, White WB. Effects of sodium-glucose co-transporter 2 inhibitors on blood pressure: a systematic review and meta-analysis. J Am Soc Hypertens. 2014 Apr;8(4):262-75.e9. doi: 10.1016/j.jash.2014.01.007. Epub 2014 Jan 26. PMID: 24602971.

J4 (ii): Oliva RV, Bakris GL. Blood pressure effects of sodium-glucose co-transport 2 (SGLT2) inhibitors. J Am Soc Hypertens. 2014 May;8(5):330-9. doi: 10.1016/j.jash.2014.02.003. Epub 2014 Feb 12. PMID: 24631482.

**J5. Systemic oestrogens with a history of breast cancer (increased risk of recurrence).**

J5 (i): Calle EE, Feigelson HS, Hildebrand JS, Teras LR, Thun MJ, Rodriguez C. Postmenopausal hormone use and breast cancer associations differ by hormone regimen and histologic subtype. Cancer 2009; 115(5): 936-45. Erratum in: Cancer 2009; 115(7): 1587. PubMed PMID: 19156895.

J5 (ii): Diergaarde B, Potter JD, Jupe ER, Manjeshwar S, Shimasaki CD, Pugh TW, Defreese DC, Gramling BA, Evans I, White E. Polymorphisms in genes involved in sex hormone metabolism, estrogen plus progestin hormone therapy use, and risk of postmenopausal breast cancer. Cancer Epidemiol Biomarkers Prev 2008; 17(7): 1751-9. PubMed PMID: 18628428.

**J6.**  **Systemic oestrogens with a history of venous thromboembolism (increased risk of recurrence).**

J6 (i): Høibraaten E, Abdelnoor M, Sandset PM. Hormone replacement therapy with estradiol and risk of venous thromboembolism--a population-based case-control study. Thromb Haemost. 1999 Oct;82(4):1218-21. PMID: 10544901.

J6 (ii): Canonico M, Oger E, Plu-Bureau G, Conard J, Meyer G, Lévesque H, Trillot N, Barrellier MT, Wahl D, Emmerich J, Scarabin PY; Estrogen and Thromboembolism Risk (ESTHER) Study Group. Hormone therapy and venous thromboembolism among postmenopausal women: impact of the route of estrogen administration and progestogens: the ESTHER study. Circulation. 2007 Feb 20;115(7):840-5. doi: 10.1161/CIRCULATIONAHA.106.642280. PMID: 17309934.

J6 (iii): Høibraaten E, Qvigstad E, Arnesen H, Larsen S, Wickstrøm E, Sandset PM. Increased risk of recurrent venous thromboembolism during hormone replacement therapy--results of the randomized, double-blind, placebo-controlled estrogen in venous thromboembolism trial (EVTET). Thromb Haemost. 2000 Dec;84(6):961-7. PMID: 11154141.

**J7: Menopausal hormone therapy (oestrogen plus progestin) with a history of stenotic coronary, cerebral or peripheral arterial disease (increased risk of acute arterial thrombosis).**

J7 (i): Rossouw JE, Anderson GL, Prentice RL, LaCroix AZ, Kooperberg C, Stefanick ML, Jackson RD, Beresford SA, Howard BV, Johnson KC, Kotchen JM, Ockene J; Writing Group for the Women's Health Initiative Investigators. Risks and benefits of estrogen plus progestin in healthy postmenopausal women: principal results From the Women's Health Initiative randomized controlled trial. JAMA. 2002 Jul 17;288(3):321-33. doi: 10.1001/jama.288.3.321. PMID: 12117397.

J7 (ii): Oliver-Williams C, Glisic M, Shahzad S, Brown E, Pellegrino Baena C, Chadni M, Chowdhury R, Franco OH, Muka T. The route of administration, timing, duration and dose of postmenopausal hormone therapy and cardiovascular outcomes in women: a systematic review. Hum Reprod Update. 2019 Mar 1;25(2):257-271. doi: 10.1093/humupd/dmy039. PMID: 30508190.

**J8. Systemic oestrogens without progestogens in patients with intact uterus (risk of endometrial cancer).**

J8 (i): Dick SE, DeWitt DE, Anawalt BD. Postmenopausal hormone replacement therapy and major clinical outcomes: a focus on cardiovascular disease, osteoporosis, dementia, and breast and endometrial neoplasia. Am J Manag Care 2002; 8(1): 95-104. Review. PubMed PMID: 11814176.

J8 (ii): Furness S, Roberts H, Marjoribanks J, Lethaby A. Hormone therapy in postmenopausal women and risk of endometrial hyperplasia. Cochrane Database Syst Rev 2012 Aug 15;8:CD000402. doi: 10.1002/14651858.CD000402.pub4. Review. PubMed PMID: 22895916.

J8 (iii): Marjoribanks J, Farquhar C, Roberts H, Lethaby A. Long term hormone therapy for perimenopausal and postmenopausal women. Cochrane Database Syst Rev 2012 Jul 11;7:CD004143. doi: 10.1002/14651858.CD004143.pub4. Review. PubMed PMID: 22786488.

**J9. Levothyroxine in subclinical hypothyroidism i.e., normal free T4, elevated TSH but < 10 mU/L (no evidence of benefit, risk of iatrogenic thyrotoxicosis).**

J9 (i): Stott DJ, Rodondi N, Kearney PM, Ford I, Westendorp RGJ, Mooijaart SP, Sattar N, Aubert CE, Aujesky D, Bauer DC, Baumgartner C, Blum MR, Browne JP, Byrne S, Collet TH, Dekkers OM, den Elzen WPJ, Du Puy RS, Ellis G, Feller M, Floriani C, Hendry K, Hurley C, Jukema JW, Kean S, Kelly M, Krebs D, Langhorne P, McCarthy G, McCarthy V, McConnachie A, McDade M, Messow M, O'Flynn A, O'Riordan D, Poortvliet RKE, Quinn TJ, Russell A, Sinnott C, Smit JWA, Van Dorland HA, Walsh KA, Walsh EK, Watt T, Wilson R, Gussekloo J; TRUST Study Group. Thyroid Hormone Therapy for Older Adults with Subclinical Hypothyroidism. N Engl J Med. 2017 Jun 29;376(26):2534-2544. doi: 10.1056/NEJMoa1603825. Epub 2017 Apr 3. PMID: 28402245.

J9 (ii): Zhao C, Wang Y, Xiao L, Li L. Effect of Levothyroxine on Older Patients With Subclinical Hypothyroidism: A Systematic Review and Meta-Analysis. Front Endocrinol (Lausanne). 2022 Jul 14;13:913749. doi: 10.3389/fendo.2022.913749. PMID: 35909574; PMCID: PMC9329610.

**J10. Vasopressin analogues (e.g. desmopressin, vasopressin) for urinary incontinence or urinary frequency (risk of symptomatic hyponatraemia).**

J10 (i): Choi EY, Park JS, Kim YT, Park SY, Kim GH. The risk of hyponatremia with desmopressin use for nocturnal polyuria. Am J Nephrol. 2015;41(3):183-90. doi: 10.1159/000381562. Epub 2015 Apr 8. PMID: 25871541.

J10 (ii): Wang CJ, Lin YN, Huang SW, Chang CH. Low dose oral desmopressin for nocturnal polyuria in patients with benign prostatic hyperplasia: a double-blind, placebo controlled, randomized study. J Urol. 2011 Jan;185(1):219-23. doi: 10.1016/j.juro.2010.08.095. Epub 2010 Nov 13. PMID: 21074790.

**Section K: Drugs that predictably increase the risk of falls in older people.**

**K1. Benzodiazepines in patients with recurrent falls (sedative, may cause reduced sensorium, impair balance).**

K1 (i): Huang AR, Mallet L, Rochefort CM, Eguale T, Buckeridge DL, Tamblyn R. Medication-related falls in the elderly: causative factors and preventive strategies. Drugs Aging 2012; 29(5): 359-76. Review. PubMed PMID: 22550966.

K1 (ii): Woolcott JC, Richardson KJ, Wiens MO, Patel B, Marin J, Khan KM, Marra CA. Meta-analysis of the impact of 9 medication classes on falls in elderly persons. Arch Intern Med 2009; 169(21): 1952-60. Review. Erratum in: Arch Intern Med 2010 Mar 8;170(5):477. PubMed PMID: 19933955.

**K2. Antipsychotic drugs in patients with recurrent falls (may cause Parkinsonism).**

K2 (i): Hill KD, Wee R. Psychotropic drug-induced falls in older people: a review of interventions aimed at reducing the problem. Drugs Aging 2012; 29(1): 15-30. Review. PubMed PMID: 22191720.

K2 (ii): Woolcott JC, Richardson KJ, Wiens MO, Patel B, Marin J, Khan KM, Marra CA. Meta-analysis of the impact of 9 medication classes on falls in elderly persons. Arch Intern Med 2009; 169(21): 1952-60. Review. Erratum in: Arch Intern Med 2010; 170(5): 477. PubMed PMID: 19933955.

**K3. Vasodilator drugs in patients with recurrent falls with persistent postural hypotension i.e., systolic BP drop ≥ 20 mmHg and/or diastolic BP drop ≥ 10 mmHg (risk of syncope, falls).**

K3 (i): Aronow WS. Treating hypertension in older adults: safety considerations. Drug Saf 2009; 32(2): 111-8. PMID: 19236118

K3 (ii): Verhaeverbeke I, Mets T. Drug-induced orthostatic hypotension in the elderly: avoiding its onset. Drug Saf 1997; 17(2): 105-18. Review. PubMed PMID: 9285201.

**K4. Hypnotic Z-drugs i.e., zopiclone, zolpidem, zaleplon in patients with recurrent falls (may cause protracted daytime sedation, ataxia).**

K4 (i): Mets MA, Volkerts ER, Olivier B, Verster JC. Effect of hypnotic drugs on body balance and standing steadiness. Sleep Med Rev 2010; 14(4): 259-67. PubMed PMID: 20171127.

K4 (ii): Shuto H, Imakyure O, Matsumoto J, Egawa T, Jiang Y, Hirakawa M, Kataoka Y, Yanagawa T. Medication use as a risk factor for inpatient falls in an acute care hospital: a case-crossover study. Br J Clin Pharmacol 2010; 69(5): 535-42. PubMed PMID: 20573090.

**K5. Anti-epileptic drugs in patients with recurrent falls (may impair sensorium, may adversely affect cerebellar function).**

K5 (i): Haasum Y, Johnell K. Use of antiepileptic drugs and risk of falls in old age: A systematic review. Epilepsy Res. 2017 Dec;138:98-104. doi: 10.1016/j.eplepsyres.2017.10.022. Epub 2017 Oct 31. PMID: 29096135.

K5 (ii): Seppala LJ, Petrovic M, Ryg J, Bahat G, Topinkova E, Szczerbińska K, van der Cammen TJM, Hartikainen S, Ilhan B, Landi F, Morrissey Y, Mair A, Gutiérrez-Valencia M, Emmelot-Vonk MH, Mora MÁC, Denkinger M, Crome P, Jackson SHD, Correa-Pérez A, Knol W, Soulis G, Gudmundsson A, Ziere G, Wehling M, O'Mahony D, Cherubini A, van der Velde N. STOPPFall (Screening Tool of Older Persons Prescriptions in older adults with high fall risk): a Delphi study by the EuGMS Task and Finish Group on Fall-Risk-Increasing Drugs. Age Ageing. 2021 Jun 28;50(4):1189-1199. doi: 10.1093/ageing/afaa249. PMID: 33349863; PMCID: PMC8244563.

**K6. First generation antihistamines in patients with recurrent falls (may impair sensorium).**

K6 (i): Cho H, Myung J, Suh HS, Kang HY. Antihistamine use and the risk of injurious falls or fracture in elderly patients: a systematic review and meta-analysis. Osteoporos Int. 2018 Oct;29(10):2163-2170. doi: 10.1007/s00198-018-4564-z. Epub 2018 Jul 25. PMID: 30046925.

K6 (ii): Seppala LJ, Petrovic M, Ryg J, Bahat G, Topinkova E, Szczerbińska K, van der Cammen TJM, Hartikainen S, Ilhan B, Landi F, Morrissey Y, Mair A, Gutiérrez-Valencia M, Emmelot-Vonk MH, Mora MÁC, Denkinger M, Crome P, Jackson SHD, Correa-Pérez A, Knol W, Soulis G, Gudmundsson A, Ziere G, Wehling M, O'Mahony D, Cherubini A, van der Velde N. STOPPFall (Screening Tool of Older Persons Prescriptions in older adults with high fall risk): a Delphi study by the EuGMS Task and Finish Group on Fall-Risk-Increasing Drugs. Age Ageing. 2021 Jun 28;50(4):1189-1199. doi: 10.1093/ageing/afaa249. PMID: 33349863; PMCID: PMC8244563.

**K7. Opioids in patients with recurrent falls (may impair sensorium).**

K7 (i): Yoshikawa A, Ramirez G, Smith ML, Foster M, Nabil AK, Jani SN, Ory MG. Opioid Use and the Risk of Falls, Fall Injuries and Fractures among Older Adults: A Systematic Review and Meta-Analysis. J Gerontol A Biol Sci Med Sci. 2020 Sep 25;75(10):1989-1995. doi: 10.1093/gerona/glaa038. PMID: 32016284.

K7 (ii): Seppala LJ, van de Glind EMM, Daams JG, Ploegmakers KJ, de Vries M, Wermelink AMAT, van der Velde N; EUGMS Task and Finish Group on Fall-Risk-Increasing Drugs. Fall-Risk-Increasing Drugs: A Systematic Review and Meta-analysis: III. Others. J Am Med Dir Assoc. 2018 Apr;19(4):372.e1-372.e8. doi: 10.1016/j.jamda.2017.12.099. Epub 2018 Mar 2. PMID: 29402646.

**K8. Antidepressants in patients with recurrent falls (may impair sensorium).**

K8 (i): Hart LA, Phelan EA, Yi JY, Marcum ZA, Gray SL. Use of Fall Risk-Increasing Drugs Around a Fall-Related Injury in Older Adults: A Systematic Review. J Am Geriatr Soc. 2020 Jun;68(6):1334-1343. doi: 10.1111/jgs.16369. Epub 2020 Feb 17. PMID: 32064594; PMCID: PMC7299782.

K8 (ii): Hartikainen S, Lönnroos E, Louhivuori K. Medication as a risk factor for falls: critical systematic review. J Gerontol A Biol Sci Med Sci. 2007 Oct;62(10):1172-81. doi: 10.1093/gerona/62.10.1172. PMID: 17921433.

K8 (iii): Leipzig RM, Cumming RG, Tinetti ME. Drugs and falls in older people: a systematic review and meta-analysis: I. Psychotropic drugs. J Am Geriatr Soc. 1999 Jan;47(1):30-9. doi: 10.1111/j.1532-5415.1999.tb01898.x. PMID: 9920227.

**K9. Alpha blockers as antihypertensives in patients with recurrent falls (may cause orthostatic hypotension).**

K9 (i): Verhaeverbeke I, Mets T. Drug-induced orthostatic hypotension in the elderly: avoiding its onset. Drug Saf. 1997 Aug;17(2):105-18. doi: 10.2165/00002018-199717020-00003. PMID: 9285201.

K9 (ii): Mansbart F, Kienberger G, Sönnichsen A, Mann E. Efficacy and safety of adrenergic alpha-1 receptor antagonists in older adults: a systematic review and meta-analysis supporting the development of recommendations to reduce potentially inappropriate prescribing. BMC Geriatr. 2022 Sep 28;22(1):771. doi: 10.1186/s12877-022-03415-7. PMID: 36171560; PMCID: PMC9516834.

**K10. Alpha blockers for prostatic bladder outflow symptoms, other than silodosin in patients with recurrent falls (may cause orthostatic hypotension).**

K10 (i): Lepor H. Alpha blockers for the treatment of benign prostatic hyperplasia. Rev Urol. 2007 Fall;9(4):181-90. PMID: 18231614; PMCID: PMC2213889.

K10 (ii): Seo JH, Han JS, Lee Y, Myong JP, Ha US. Fall risk related to subtype-specific alpha-antagonists for benign prostatic hyperplasia: a nationwide Korean population-based cohort study. World J Urol. 2022 Dec;40(12):3043-3048. doi: 10.1007/s00345-022-04195-w. Epub 2022 Oct 31. PMID: 36315286.

**K11. Centrally acting antihypertensives (may impair sensorium and may cause orthostatic hypotension).**

K11 (i). Potter JF. Hypertension. In: Brocklehurst’s Textbook of Geriatric Medicine & Gerontology, 6^th^ edition, Churchill Livingstone, 2003, p403.

K11 (ii). Khindri S, Jackson S. Hypertension. In: Prescribing for Elderly Patients, S. Jackson, P. Jansen, A. Mangoni, eds., Wiley-Blackwell, Chichester, UK, 2009, pp97-98.

**K12. Antimuscarinics for treatment of overactive bladder or urge incontinence (may impair sensorium).**

K12 (i): Kay GG, Granville LJ. Antimuscarinic agents: implications and concerns in the management of overactive bladder in the elderly. Clin Ther. 2005 Jan;27(1):127-38; quiz 139-40. doi: 10.1016/j.clinthera.2005.01.006. PMID: 15763613.

K12 (ii): Feinberg M. The problems of anticholinergic adverse effects in older patients. Drugs Aging. 1993 Jul-Aug;3(4):335-48. doi: 10.2165/00002512-199303040-00004. PMID: 8369593.

**Section L: Analgesic Drugs.**

**L1. Use of oral or transdermal strong opioids (morphine, oxycodone, fentanyl, buprenorphine, diamorphine, methadone, tramadol, pethidine, pentazocine) as first line therapy for mild pain (WHO analgesic ladder not observed; paracetamol or NSAID not prescribed as first-line therapy).**

L1 (i): World Health Organization (WHO). Cancer pain relief. With a guide to opioid availability, 2nd ed. Geneva 1996: WHO. ISBN 92-4-154482-1.

L1 (ii): Scoping Document for WHO Guidelines for the pharmacological treatment of persisting pain in adults with medical illnesses, Geneva 2012, GRC-08-04-0052A, p 9. <http://www.who.int/medicines/areas/quality_safety/Scoping_WHO_GLs_PersistPainAdults_webversion.pdf/> ; accessed Dec 28, 2012.

**L2. Use of regular (as distinct from PRN) opioids without concomitant laxative (risk of severe constipation).**

L2 (i): Forman WB. Opioid analgesic drugs in the elderly. Clin Geriatr Med 1996; 12(3): 489-500. Review. PubMed PMID: 8853941

L2 (ii): Kalso E, Edwards JE, Moore RA, McQuay HJ. Opioids in chronic non-cancer pain: systematic review of efficacy and safety. Pain 2004; 112(3): 372-80. PubMed PMID: 15561393.

**L3. Long-acting opioids without short-acting opioids for break-through moderate or severe pain (risk of non-control of severe pain)**

L3 (i): Johnson AG, Seideman P, Day RO. Adverse drug interactions with nonsteroidal anti-inflammatory drugs (NSAIDs). Recognition, management and avoidance. Drug Saf 1993; 8(2): 99-127. Review. PubMed PMID: 8452660.

L3 (ii): Zullo A, Hassan C, Campo SM, Morini S. Bleeding peptic ulcer in the elderly: risk factors and prevention strategies. Drugs Aging 2007; 24(10): 815-28. Review. PubMed PMID: 17896831.

L3 (iii): Peng S, Duggan A. Gastrointestinal adverse effects of non-steroidal anti-inflammatory drugs. Expert Opin Drug Saf 2005; 4(2): 157-69. Review. PubMed PMID: 15794710.

L3 (iv): Peura DA. Prevention of nonsteroidal anti-inflammatory drug-associated gastrointestinal symptoms and ulcer complications. Am J Med 2004; 117 Suppl 5A: 63S-71S. Review. PubMed PMID: 15478855.

**L4. Topical lidocaine (lignocaine) patch for treatment of chronic osteoarthritis pain (no clear-cut evidence of efficacy).**

L4 (i): Voute M, Morel V, Pickering G. Topical Lidocaine for Chronic Pain Treatment. Drug Des Devel Ther. 2021 Sep 29;15:4091-4103. doi: 10.2147/DDDT.S328228. PMID: 34616143; PMCID: PMC8487862.

L4 (ii): Bruyère O, Cooper C, Pelletier J-P, Maheu E, Rannou F, Branco J et al. A consensus statement on the European Society for Clinical and Economic Aspects of Osteoporosis and Osteoarthritis (ESCEO) algorithm for the management of knee osteoarthritis— from evidence-based medicine to the real-life setting. Semin Arthritis Rheum 2016; 45:S3–S11

**L5. Gabapentinoids (e.g., gabapentin, pregabalin) for non-neuropathic pain (lack of evidence of efficacy).**

L5 (i): Mathieson S, Lin CC, Underwood M, Eldabe S. Pregabalin and gabapentin for pain. BMJ. 2020 Apr 28;369:m1315. doi: 10.1136/bmj.m1315. PMID: 32345589.

L5 (ii): Goodman CW, Brett AS. Gabapentin and Pregabalin for Pain - Is Increased Prescribing a Cause for Concern? N Engl J Med. 2017 Aug 3;377(5):411-414. doi: 10.1056/NEJMp1704633. PMID: 28767350.

**L6. Paracetamol at doses ≥ 3 g/24 hours in patients with poor nutritional status i.e., BMI < 18 or chronic liver disease (risk of hepatotoxicity).**

L6 (i): Reid O, Ngo J, Lalic S, Su E, Elliott RA. Paracetamol dosing in hospital and on discharge for older people who are frail or have low body weight. Br J Clin Pharmacol. 2022 May 10. doi: 10.1111/bcp.15394. Epub ahead of print. PMID: 35535712.

L6 (ii): O'Neil CK, Hanlon JT, Marcum ZA. Adverse effects of analgesics commonly used by older adults with osteoarthritis: focus on non-opioid and opioid analgesics. Am J Geriatr Pharmacother. 2012 Dec;10(6):331-42. doi: 10.1016/j.amjopharm.2012.09.004. Epub 2012 Oct 2. PMID: 23036838; PMCID: PMC3529168.

**Section M: Antimuscarinic/anticholinergic drug burden.**

**M1: Concomitant use of two or more drugs with antimuscarinic/anticholinergic properties (e.g., bladder antispasmodics, intestinal antispasmodics, tricyclic antidepressants, first generation antihistamines, antipsychotics) (risk of increased antimuscarinic/anticholinergic toxicity).**

M1 (i): Feinberg M. The problems of anticholinergic adverse effects in older patients. Drugs Aging 1993; 3(4): 335-48. Review. PubMed PMID: 8369593.

M1 (ii): Gerretsen P, Pollock BG. Drugs with anticholinergic properties: a current perspective on use and safety. Expert Opin Drug Saf 2011; 10(5): 751-65. Review. PubMed PMID: 21635190.

M1 (iii): Karimi S, Dharia SP, Flora DS, Slattum PW. Anticholinergic burden: clinical implications for seniors and strategies for clinicians. Consult Pharm 2012; 27(8): 564-82. Review. PubMed PMID: 22910177.

**Appendix 2: START Criteria References**

**Section A: Indicated drugs.**

**A1. Where a drug is clearly indicated and considered appropriate in the particular clinical context and there is no clear contraindication, that drug should be initiated as per formulary guidelines for dose and duration.**

A1: No reference. Self-evident.

**Section B: Cardiovascular System**

**B1. Antihypertensive therapy where systolic blood pressure > 140 mmHg and /or diastolic blood pressure > 90 mmHg, unless established moderate or severe physical frailty in whom the threshold for therapy is 150 mmHg systolic pressure and/or 90 mmHg diastolic pressure.**

B1 (i): Ferri C, Ferri L, Desideri G. Management of Hypertension in the Elderly and Frail Elderly. High Blood Press Cardiovasc Prev. 2017 Mar;24(1):1-11. doi: 10.1007/s40292-017-0185-4. Epub 2017 Feb 8. PMID: 28181201.

B1 (ii): Benetos A, Bulpitt CJ, Petrovic M, Ungar A, Agabiti Rosei E, Cherubini A, Redon J, Grodzicki T, Dominiczak A, Strandberg T, Mancia G. An Expert Opinion From the European Society of Hypertension-European Union Geriatric Medicine Society Working Group on the Management of Hypertension in Very Old, Frail Subjects. Hypertension. 2016 May;67(5):820-5. doi: 10.1161/HYPERTENSIONAHA.115.07020. Epub 2016 Mar 14. PMID: 26975708.

B1 (iii): Williamson JD, Supiano MA, Applegate WB, Berlowitz DR, Campbell RC, Chertow GM, Fine LJ, Haley WE, Hawfield AT, Ix JH, Kitzman DW, Kostis JB, Krousel-Wood MA, Launer LJ, Oparil S, Rodriguez CJ, Roumie CL, Shorr RI, Sink KM, Wadley VG, Whelton PK, Whittle J, Woolard NF, Wright JT Jr, Pajewski NM; SPRINT Research Group. Intensive vs Standard Blood Pressure Control and Cardiovascular Disease Outcomes in Adults Aged ≥75 Years: A Randomized Clinical Trial. JAMA 2016; 315(24):2673-82. doi: 10.1001/jama.2016.7050. PMID: 27195814; PMCID: PMC4988796.

B1 (iv): Mühlbauer V, Dallmeier D, Brefka S, Bollig C, Voigt-Radloff S, Denkinger M. The Pharmacological Treatment of Arterial Hypertension in Frail, Older Patients—a Systematic Review. Dtsch Arztebl Int. 2019 Jan 18;116(3):23-30. doi: 10.3238/arztebl.2019.0023. PMID: 30832759; PMCID: PMC6401515.

B1 (v): Mühlbauer V, Dallmeier D, Brefka S, Bollig C, Voigt-Radloff S, Denkinger M. The Pharmacological Treatment of Arterial Hypertension in Frail, Older Patients—a Systematic Review. Dtsch Arztebl Int. 2019 Jan 18;116(3):23-30. doi: 10.3238/arztebl.2019.0023. PMID: 30832759; PMCID: PMC6401515.

**B2. Statin therapy with a documented history of coronary, cerebral or peripheral vascular disease, unless the patient’s status is end-of-life or established moderate or severe frailty.**

B2 (i): Mills EJ, Wu P, Chong G, Ghement I, Singh S, Akl EA, Eyawo O, Guyatt G,Berwanger O, Briel M. Efficacy and safety of statin treatment for cardiovascular disease: a network meta-analysis of 170,255 patients from 76 randomized trials. QJM 2011; 104(2): 109-24. Review. PubMed PMID: 20934984.

B2 (ii): Brugts JJ, Yetgin T, Hoeks SE, Gotto AM, Shepherd J, Westendorp RG, de CraenAJ, Knopp RH, Nakamura H, Ridker P, van Domburg R, Deckers JW. The benefits of statins in people without established cardiovascular disease but with cardiovascular risk factors: meta-analysis of randomised controlled trials. BMJ 2009; 338: b2376. Review. PubMed PMID: 19567909.

B2 (iii): Amarenco P, Labreuche J. Lipid management in the prevention of stroke: review and updated meta-analysis of statins for stroke prevention. Lancet Neurol 2009; 8(5): 453-63. Review. PubMed PMID:19375663.

B2 (iv): Chou R, Dana T, Blazina I, Daeges M, Jeanne TL. Statins for Prevention of Cardiovascular Disease in Adults: Evidence Report and Systematic Review for the US Preventive Services Task Force. JAMA 2016 Nov 15;316(19):2008-2024. doi: 10.1001/jama.2015.15629. Erratum in: JAMA. 2020 Feb 18;323(7):669. PMID: 27838722.

**B3. Angiotensin Converting Enzyme (ACE) inhibitor with coronary artery disease.**

B3 (i): Dagenais GR, Pogue J, Fox K, Simoons ML, Yusuf S. Angiotensin-converting-enzyme inhibitors in stable vascular disease without left ventricular systolic dysfunction or heart failure: a combined analysis of three trials. Lancet 2006; 368(9535): 581-8. Review. PubMed PMID: 16905022.

B3 (ii): Danchin N, Cucherat M, Thuillez C, Durand E, Kadri Z, Steg PG. Angiotensin-converting enzyme inhibitors in patients with coronary artery disease and absence of heart failure or left ventricular systolic dysfunction: an overview of long-term randomized controlled trials. Arch Intern Med 2006; 166(7): 787-96. PubMed PMID: 16606817.

B3 (iii): McAlister FA; Renin Angiotension System Modulator Meta-Analysis Investigators. Angiotensin-converting enzyme inhibitors or angiotensin receptor blockers are beneficial in normotensive atherosclerotic patients: a collaborative meta-analysis of randomized trials. Eur Heart J 2012; 33(4): 505-14. PubMed PMID: 22041554.

**B4. Beta-blocker with symptomatic coronary artery disease.**

B4 (i): Bangalore S, Steg G, Deedwania P, Crowley K, Eagle KA, Goto S, Ohman EM, Cannon CP, Smith SC, Zeymer U, Hoffman EB, Messerli FH, Bhatt DL; REACH Registry Investigators. β-Blocker use and clinical outcomes in stable outpatients with and without coronary artery disease. JAMA. 2012 Oct 3;308(13):1340-9. doi: 10.1001/jama.2012.12559. PMID: 23032550.

B4 (ii): Andreasen C, Andersson C. Current use of beta-blockers in patients with coronary artery disease. Trends Cardiovasc Med. 2018 Aug;28(6):382-389. doi: 10.1016/j.tcm.2017.12.014. Epub 2018 Jan 5. PMID: 29373178.

B4 (iii): Freemantle N, Cleland J, Young P, Mason J, Harrison J. beta Blockade after myocardial infarction: systematic review and meta regression analysis. BMJ. 1999 Jun 26;318(7200):1730–7.

**B5: Angiotensin Converting Enzyme (ACE) inhibitor for heart failure with reduced ejection fraction.**

B5 (i): [SOLVD Investigators, Yusuf S, Pitt B, et al. Effect of enalapril on mortality and the development of heart failure in asymptomatic patients with reduced left ventricular ejection fractions. N Engl J Med 1992; 327:685.](https://www.uptodate.com/contents/initial-pharmacologic-therapy-of-heart-failure-with-reduced-ejection-fraction-in-adults/abstract/26)

B5 (ii): [Cohn JN, Johnson G, Ziesche S, et al. A comparison of enalapril with hydralazine-isosorbide dinitrate in the treatment of chronic congestive heart failure. N Engl J Med 1991; 325:303.](https://www.uptodate.com/contents/initial-pharmacologic-therapy-of-heart-failure-with-reduced-ejection-fraction-in-adults/abstract/27)

B5 (iii): [CONSENSUS Trial Study Group. Effects of enalapril on mortality in severe congestive heart failure. Results of the Cooperative North Scandinavian Enalapril Survival Study (CONSENSUS). N Engl J Med 1987; 316:1429.](https://www.uptodate.com/contents/initial-pharmacologic-therapy-of-heart-failure-with-reduced-ejection-fraction-in-adults/abstract/28)

B5 (iv): [SOLVD Investigators, Yusuf S, Pitt B, et al. Effect of enalapril on survival in patients with reduced left ventricular ejection fractions and congestive heart failure. N Engl J Med 1991; 325:293.](https://www.uptodate.com/contents/initial-pharmacologic-therapy-of-heart-failure-with-reduced-ejection-fraction-in-adults/abstract/29)

B5 (v): [Flather MD, Yusuf S, Køber L, et al. Long-term ACE-inhibitor therapy in patients with heart failure or left-ventricular dysfunction: a systematic overview of data from individual patients. ACE-Inhibitor Myocardial Infarction Collaborative Group. Lancet 2000; 355:1575.](https://www.uptodate.com/contents/initial-pharmacologic-therapy-of-heart-failure-with-reduced-ejection-fraction-in-adults/abstract/30)

**B6. Cardioselective beta-blocker (bisoprolol, nebivolol, metoprolol or carvedilol) for stable heart failure with reduced ejection fraction.**

B6 (i): [Packer M, Bristow MR, Cohn JN, et al. The effect of carvedilol on morbidity and mortality in patients with chronic heart failure. U.S. Carvedilol Heart Failure Study Group. N Engl J Med 1996; 334:1349.](https://www.uptodate.com/contents/initial-pharmacologic-therapy-of-heart-failure-with-reduced-ejection-fraction-in-adults/abstract/48)

B6 (ii): [Leizorovicz A, Lechat P, Cucherat M, Bugnard F. Bisoprolol for the treatment of chronic heart failure: a meta-analysis on individual data of two placebo-controlled studies--CIBIS and CIBIS II. Cardiac Insufficiency Bisoprolol Study. Am Heart J 2002; 143:301.](https://www.uptodate.com/contents/initial-pharmacologic-therapy-of-heart-failure-with-reduced-ejection-fraction-in-adults/abstract/55)

B6 (iii): [Brophy JM, Joseph L, Rouleau JL. Beta-blockers in congestive heart failure. A Bayesian meta-analysis. Ann Intern Med 2001; 134:550.](https://www.uptodate.com/contents/initial-pharmacologic-therapy-of-heart-failure-with-reduced-ejection-fraction-in-adults/abstract/56)

**B7. Mineralocorticoid receptor antagonist (spironolactone, eplerenone) in heart failure without severe renal function impairment i.e. eGFR > 30 ml/min/m^2^.**

B7 (i): [Zannad F, McMurray JJ, Krum H, et al. Eplerenone in patients with systolic heart failure and mild symptoms. N Engl J Med 2011; 364:11.](https://www.uptodate.com/contents/secondary-pharmacologic-therapy-in-heart-failure-with-reduced-ejection-fraction-hfref-in-adults/abstract/4)

B7 (ii): [Pitt B, Zannad F, Remme WJ, et al. The effect of spironolactone on morbidity and mortality in patients with severe heart failure. Randomized Aldactone Evaluation Study Investigators. N Engl J Med 1999; 341:709.](https://www.uptodate.com/contents/secondary-pharmacologic-therapy-in-heart-failure-with-reduced-ejection-fraction-hfref-in-adults/abstract/9)

**B8. SGLT-2 inhibitors (canagliflozin, dapagliflozin, empagliflozin, ertugliflozin) in symptomatic heart failure with or without reduced ejection fraction regardless of diabetes being present or not.**

B8 (i): [McMurray JJV, Solomon SD, Inzucchi SE, et al. Dapagliflozin in Patients with Heart Failure and Reduced Ejection Fraction. N Engl J Med 2019; 381:1995.](https://www.uptodate.com/contents/secondary-pharmacologic-therapy-in-heart-failure-with-reduced-ejection-fraction-hfref-in-adults/abstract/16)

B8 (ii): [Packer M, Anker SD, Butler J, et al. Cardiovascular and Renal Outcomes with Empagliflozin in Heart Failure. N Engl J Med 2020; 383:1413.](https://www.uptodate.com/contents/secondary-pharmacologic-therapy-in-heart-failure-with-reduced-ejection-fraction-hfref-in-adults/abstract/17)

B8 (iii): [Zannad F, Ferreira JP, Pocock SJ, et al. SGLT2 inhibitors in patients with heart failure with reduced ejection fraction: a meta-analysis of the EMPEROR-Reduced and DAPA-HF trials. Lancet 2020; 396:819.](https://www.uptodate.com/contents/secondary-pharmacologic-therapy-in-heart-failure-with-reduced-ejection-fraction-hfref-in-adults/abstract/21)

B8 (iv): Key Takeaways from the 2022 ACC/AHA/HFSA Guideline for the Management of Heart Failure. <https://www.ajmc.com/view/key-takeaways-from-the-2022-acc-aha-hfsa-guideline-for-the-management-of-heart-failure>

**B9: Sacubitril/valsartan in heart failure with reduced ejection fraction causing persistent heart failure symptoms despite optimal dose of ACE inhibitor or Angiotensin Receptor Blocker (Sacubitril/valsartan to replace ACE inhibitor or Angiotensin Receptor Blocker).**

B9 (i): [McMurray JJ, Packer M, Desai AS, et al. Angiotensin-neprilysin inhibition versus enalapril in heart failure. N Engl J Med 2014; 371:993.](https://www.uptodate.com/contents/initial-pharmacologic-therapy-of-heart-failure-with-reduced-ejection-fraction-in-adults/abstract/14)

B9 (ii): [Desai AS, McMurray JJ, Packer M, et al. Effect of the angiotensin-receptor-neprilysin inhibitor LCZ696 compared with enalapril on mode of death in heart failure patients. Eur Heart J 2015; 36:1990.](https://www.uptodate.com/contents/initial-pharmacologic-therapy-of-heart-failure-with-reduced-ejection-fraction-in-adults/abstract/15)

B9 (iii): [Desai AS, Claggett BL, Packer M, et al. Influence of Sacubitril/Valsartan (LCZ696) on 30-Day Readmission After Heart Failure Hospitalization. J Am Coll Cardiol 2016; 68:241.](https://www.uptodate.com/contents/initial-pharmacologic-therapy-of-heart-failure-with-reduced-ejection-fraction-in-adults/abstract/16)

**B10. Beta-blocker for chronic atrial fibrillation with uncontrolled heart rate.**

B10 (i): Hindricks G, Potpara T, Dagres N, Arbelo E, Bax JJ, Blomström-Lundqvist C, Boriani G, Castella M, Dan GA, Dilaveris PE, Fauchier L, Filippatos G, Kalman JM, La Meir M, Lane DA, Lebeau JP, Lettino M, Lip GYH, Pinto FJ, Thomas GN, Valgimigli M, Van Gelder IC, Van Putte BP, Watkins CL; ESC Scientific Document Group. 2020 ESC Guidelines for the diagnosis and management of atrial fibrillation developed in collaboration with the European Association for Cardio-Thoracic Surgery (EACTS): The Task Force for the diagnosis and management of atrial fibrillation of the European Society of Cardiology (ESC) Developed with the special contribution of the European Heart Rhythm Association (EHRA) of the ESC. Eur Heart J. 2021 Feb 1;42(5):373-498. doi: 10.1093/eurheartj/ehaa612. Erratum in: Eur Heart J. 2021 Feb 1;42(5):507. Erratum in: Eur Heart J. 2021 Feb 1;42(5):546-547. Erratum in: Eur Heart J. 2021 Oct 21;42(40):4194. PMID: 32860505.

B10 (ii): Xu T, Huang Y, Zhou H, Bai Y, Huang X, Hu Y, Xu D, Zhang Y, Zhang J. β-blockers and risk of all-cause mortality in patients with chronic heart failure and atrial fibrillation-a meta-analysis. BMC Cardiovasc Disord. 2019 Jun 3;19(1):135. doi: 10.1186/s12872-019-1079-2. PMID: 31159740; PMCID: PMC6547467.

**B11. Intravenous iron for symptomatic heart failure with reduced ejection fraction and iron deficiency.**

B11 (i): Anker SD, Comin Colet J, Filippatos G, Willenheimer R, Dickstein K, Drexler H, Lüscher TF, Bart B, Banasiak W, Niegowska J, Kirwan BA, Mori C, von Eisenhart Rothe B, Pocock SJ, Poole-Wilson PA, Ponikowski P; FAIR-HF Trial Investigators. Ferric carboxymaltose in patients with heart failure and iron deficiency. N Engl J Med. 2009 Dec 17;361(25):2436-48. doi: 10.1056/NEJMoa0908355. Epub 2009 Nov 17. PMID: 19920054.

B11 (ii): Ponikowski P, Kirwan BA, Anker SD, McDonagh T, Dorobantu M, Drozdz J, Fabien V, Filippatos G, Göhring UM, Keren A, Khintibidze I, Kragten H, Martinez FA, Metra M, Milicic D, Nicolau JC, Ohlsson M, Parkhomenko A, Pascual-Figal DA, Ruschitzka F, Sim D, Skouri H, van der Meer P, Lewis BS, Comin-Colet J, von Haehling S, Cohen-Solal A, Danchin N, Doehner W, Dargie HJ, Motro M, Butler J, Friede T, Jensen KH, Pocock S, Jankowska EA; AFFIRM-AHF investigators. Ferric carboxymaltose for iron deficiency at discharge after acute heart failure: a multicentre, double-blind, randomised, controlled trial. Lancet. 2020 Dec 12;396(10266):1895-1904. doi: 10.1016/S0140-6736(20)32339-4. Epub 2020 Nov 13. Erratum in: Lancet. 2021 Nov 27;398(10315):1964. PMID: 33197395.

**Section C: Coagulation System**

**C1. Vitamin K antagonists or direct thrombin inhibitors or factor Xa inhibitors in the presence of chronic or paroxysmal atrial fibrillation.**

C1 (i): Hughes M, Lip GY; Guideline Development Group, National Clinical Guideline for Management of Atrial Fibrillation in Primary and Secondary Care, National Institute for Health and Clinical Excellence. Stroke and thromboembolism inatrial fibrillation: a systematic review of stroke risk factors, risk stratification schema and cost effectiveness data. Thromb Haemost 2008; 99(2): 295-304. Review. PubMed PMID: 18278178.

C1 (ii): Dentali F, Riva N, Crowther M, Turpie AG, Lip GY, Ageno W. Efficacy and safety of the novel oral anticoagulants in atrial fibrillation: a systematic review and meta-analysis of the literature. Circulation 2012; 126(20): 2381-91. Review. PubMed PMID:23071159.

C1 (iii): Hart RG, Pearce LA, Aguilar MI. Meta-analysis: antithrombotic therapy to prevent stroke in patients who have non-valvular atrial fibrillation. Ann Intern Med 2007; 146(12): 857-67. PubMed PMID: 17577005.

C1 (iv): Aguilar MI, Hart R. Oral anticoagulants for preventing stroke in patients with non-valvular atrial fibrillation and no previous history of stroke or transient ischemic attacks. Cochrane Database of Systematic Reviews 2005, Issue 3. Art. No.: CD001927. DOI: 10.1002/14651858.CD001927.pub2.

**C2. Antiplatelet therapy (aspirin or clopidogrel or prasugrel or ticagrelor) with a documented history of coronary, cerebral or peripheral vascular disease.**

C2 (i): Zuckerman IH, Yin X, Rattinger GB, Gottlieb SS, Simoni-Wastila L, Pierce SA,Huang TY, Shenolikar R, Stuart B. Effect of exposure to evidence-based pharmacotherapy on outcomes after acute myocardial infarction in older adults. J Am Geriatr Soc 2012; 60(10): 1854-61. PubMed PMID: 23003000.

C2 (ii): Alonso-Coello P, Bellmunt S, McGorrian C, Anand SS, Guzman R, Criqui MH, AklEA, Olav Vandvik P, Lansberg MG, Guyatt GH, Spencer FA; American College of Chest Physicians. Antithrombotic therapy in peripheral artery disease: Antithrombotic Therapy and Prevention of Thrombosis, 9th ed: American College of Chest Physicians Evidence-Based Clinical Practice Guidelines. Chest 2012; 141(2Suppl): e669S-90S. PubMed PMID: 22315275.

C2 (iii): Fleg JL, Aronow WS, Frishman WH. Cardiovascular drug therapy in the elderly: benefits and challenges. Nat Rev Cardiol 2011; 8(1): 13-28. Review. PubMed PMID: 20978470.

C2 (iv): Vandvik PO, Lincoff AM, Gore JM, Gutterman DD, Sonnenberg FA, Alonso-Coello P,Akl EA, Lansberg MG, Guyatt GH, Spencer FA; American College of Chest Physicians. Primary and secondary prevention of cardiovascular disease: Antithrombotic Therapy and Prevention of Thrombosis, 9th ed: American College of Chest Physicians Evidence-Based Clinical Practice Guidelines. Chest 2012; 141(2Suppl): e637S-68S. Erratum in: Chest 2012; 141(4): 1129. Dosage error in article text. PubMed PMID: 22315274.

**Section D: Central Nervous System.**

**D1. L-DOPA or a dopamine agonist in idiopathic Parkinson’s disease with functional impairment and resultant disability.**

D1 (i): Marjama-Lyons JM, Koller WC. Parkinson's disease. Update in diagnosis and symptom management. Geriatrics 2001; 56(8): 24-5, 29-30, 33-5. Review. PubMed PMID: 11505857.

D1 (ii): Danisi F. Parkinson's disease. Therapeutic strategies to improve patient function and quality of life. Geriatrics 2002; 57(3): 46-50; quiz 52. Review. PubMed PMID: 11899548.

**D2. Non-TCA antidepressant drug for major depression.**

D2(i): Lebowitz BD, Pearson JL, Schneider LS, Reynolds CF 3rd, Alexopoulos GS, Bruce ML, Conwell Y, Katz IR, Meyers BS, Morrison MF, Mossey J, Niederehe G, ParmeleeP. Diagnosis and treatment of depression in late life. Consensus statement update. JAMA 1997; 278(14): 1186-90. Review. PubMed PMID: 9326481.

D2(ii): Mottram P, Wilson K, Strobl J. Antidepressants for depressed elderly. Cochrane

Database Syst Rev. 2006 Jan 25;(1):CD003491. Review. PubMed PMID: 16437456.

**D3. Acetylcholinesterase inhibitor (donepezil, rivastigmine, galantamine) for mild-moderate Alzheimer’s dementia.**

D3(i): Raina P, Santaguida P, Ismaila A, Patterson C, Cowan D, Levine M, Booker L, Oremus M. Effectiveness of cholinesterase inhibitors and memantine for treating dementia: evidence review for a clinical practice guideline. Ann Intern Med 2008; 148(5): 379-97. Review. PubMed PMID: 18316756.

D3(ii): Birks J. Cholinesterase inhibitors for Alzheimer's disease. Cochrane Database Syst Rev 2006 Jan 25;(1):CD005593. Review. PubMed PMID: 16437532.

**D4. Rivastigmine for Dementia with Lewy Bodies or Parkinson’s disease dementia.**

D4 (i): Rolinski M, Fox C, Maidment I, McShane R. Cholinesterase inhibitors for dementia with Lewy bodies, Parkinson's disease dementia and cognitive impairment in Parkinson's disease. Cochrane Database Syst Rev 2012 Mar 14;3:CD006504. doi: 10.1002/14651858.CD006504.pub2. Review. PubMed PMID: 22419314.

D4 (ii): Noufi P, Khoury R, Jeyakumar S, Grossberg GT. Use of Cholinesterase Inhibitors in Non-Alzheimer's Dementias. Drugs Aging. 2019 Aug;36(8):719-731. doi: 10.1007/s40266-019-00685-6. PMID: 31201687.

**D5. Selective serotonin reuptake inhibitor (or SNRI or pregabalin if SSRI contraindicated) for persistent severe anxiety that affects independent functioning and quality of life.**

D5 (i): Ballenger JC. Remission rates in patients with anxiety disorders treated with paroxetine. J Clin Psychiatry 2004; 65(12):1696-707. PubMed PMID: 15641876.

D5 (ii): Allgulander C, Hartford J, Russell J, Ball S, Erickson J, Raskin J, Rynn M. Pharmacotherapy of generalized anxiety disorder: results of duloxetine treatment from a pooled analysis of three clinical trials. Curr Med Res Opin 2007; 23(6): 1245-52. PubMed PMID: 17559726.

D5 (iii): Rickels K, Rynn M, Iyengar M, Duff D. Remission of generalized anxiety disorder: a review of the paroxetine clinical trials database. J Clin Psychiatry 2006; 67(1): 41-7. PubMed PMID: 16426087.

D5 (iv): National Institute for Health and Clinical Excellence. Generalized anxiety disorder and panic disorder (with or without agoraphobia) in adults. Clinical Guideline 113. 2011.

**D6. Dopamine agonist (ropinirole or pramipexole or rotigotine) for Restless Legs Syndrome, once iron deficiency and severe chronic kidney disease (i.e., eGFR < 30 ml/min/m^2^) have been excluded.**

D6 (i): Zintzaras E, Kitsios GD, Papathanasiou AA, Konitsiotis S, Miligkos M, Rodopoulou P, Hadjigeorgiou GM. Randomized trials of dopamine agonists in restless legs syndrome: a systematic review, quality assessment, and meta-analysis. Clin Ther 2010; 32(2): 221-37. Review. PubMed PMID: 20206780.

D6 (ii): Hansen RA, Song L, Moore CG, Gilsenan AW, Kim MM, Calloway MO, Murray MD. Effect of ropinirole on sleep outcomes in patients with restless legs syndrome: meta-analysis of pooled individual patient data from randomized controlled trials. Pharmacotherapy 2009; 29(3): 255-62. PubMed PMID: 19249945.

D6 (iii): Scholz H, Trenkwalder C, Kohnen R, Riemann D, Kriston L, Hornyak M. Dopamine agonists for restless legs syndrome. Cochrane Database Syst Rev. 2011 Mar 16;(3):CD006009. doi: 10.1002/14651858.CD006009.pub2. Review. PubMed PMID: 21412893.

**D7. Propranolol for essential tremor with functional impairment and resultant disability.**

D7 (i): Zappia M, Albanese A, Bruno E, Colosimo C, Filippini G, Martinelli P, Nicoletti A, Quattrocchi G, Abbruzzese G, Berardelli A, Allegra R, Aniello MS, Elia AE, Martino D, Murgia D, Picillo M, Squintani G. Treatment of essential tremor: a systematic review of evidence and recommendations from the Italian Movement Disorders Association. J Neurol. 2013 Mar;260(3):714-40. doi: 10.1007/s00415-012-6628-x. Epub 2012 Aug 11. PMID: 22886006.

D7 (ii): Ferreira JJ, Mestre TA, Lyons KE, Benito-León J, Tan EK, Abbruzzese G, Hallett M, Haubenberger D, Elble R, Deuschl G; MDS Task Force on Tremor and the MDS Evidence Based Medicine Committee. MDS evidence-based review of treatments for essential tremor. Mov Disord. 2019 Jul;34(7):950-958. doi: 10.1002/mds.27700. Epub 2019 May 2. PMID: 31046186.

**Section E: Renal System**

**E1. One-alpha hydroxycholecalciferol or calcitriol supplementation in severe chronic kidney (i.e., eGFR < 30 ml/min/m^2^) disease with hypocalcaemia (corrected serum calcium < 2.10 mmol/l) and associated secondary hyperparathyroidism.**

E1 (i): Moe SM, Drüeke TB. Management of secondary hyperparathyroidism: the importance and the challenge of controlling parathyroid hormone levels without elevating calcium, phosphorus, and calcium-phosphorus product. Am J Nephrol. 2003 Nov-Dec;23(6):369-79. doi: 10.1159/000073945. Epub 2003 Oct 9. PMID: 14551461.

E1 (ii): Yuen NK, Ananthakrishnan S, Campbell MJ. Hyperparathyroidism of Renal Disease. Perm J. 2016 Summer;20(3):15-127. doi: 10.7812/TPP/15-127. Epub 2016 Jul 22. PMID: 27479950; PMCID: PMC4991918.

**E2. Phosphate binder in severe chronic kidney disease (i.e., eGFR < 30 ml/min/m2) if serum phosphate concentration persistently >1.76 mmol/l (5.5 mg/dl) despite adherence to renal diet.**

E2 (i): Floege J. Phosphate binders in chronic kidney disease: a systematic review of recent data. J Nephrol. 2016 Jun;29(3):329-340. doi: 10.1007/s40620-016-0266-9. Epub 2016 Jan 22. PMID: 26800972.

E2 (ii): Barreto FC, Barreto DV, Massy ZA, Drüeke TB. Strategies for Phosphate Control in Patients With CKD. Kidney Int Rep. 2019 Jun 20;4(8):1043-1056. doi: 10.1016/j.ekir.2019.06.002. PMID: 31440695; PMCID: PMC6698320.

**E3. Erythopoietin analogue in severe chronic kidney disease (i.e., eGFR < 30 ml/min/m^2^) with symptomatic anaemia not attributable to haematinic or iron deficiency to achieve a haemoglobin concentration of 10.0 to 12.0 g/dl.**

E3 (i): Mikhail A, Brown C, Williams JA, Mathrani V, Shrivastava R, Evans J, Isaac H, Bhandari S. Renal association clinical practice guideline on Anaemia of Chronic Kidney Disease. BMC Nephrol. 2017 Nov 30;18(1):345. doi: 10.1186/s12882-017-0688-1. PMID: 29191165; PMCID: PMC5709852.

E3 (ii): Locatelli F, Bárány P, Covic A, De Francisco A, Del Vecchio L, Goldsmith D, Hörl W, London G, Vanholder R, Van Biesen W; ERA-EDTA ERBP Advisory Board. Kidney Disease: Improving Global Outcomes guidelines on anaemia management in chronic kidney disease: a European Renal Best Practice position statement. Nephrol Dial Transplant. 2013 Jun;28(6):1346-59. doi: 10.1093/ndt/gft033. Epub 2013 Apr 12. PMID: 23585588.

**E4: Angiotensin receptor blocker (ARB) or Angiotensin Converting Enzyme Inhibitor (ACE-I) in chronic kidney disease with proteinuria i.e., urine albumin excretion >300 mg/24 hours.**

E4 (i): Cheung AK, Chang TI, Cushman WC, Furth SL, Hou FF, Ix JH, Knoll GA, Muntner P, Pecoits-Filho R, Sarnak MJ, Tobe SW, Tomson CRV, Lytvyn L, Craig JC, Tunnicliffe DJ, Howell M, Tonelli M, Cheung M, Earley A, Mann JFE. Executive summary of the KDIGO 2021 Clinical Practice Guideline for the Management of Blood Pressure in Chronic Kidney Disease. Kidney Int. 2021 Mar;99(3):559-569. doi: 10.1016/j.kint.2020.10.026. PMID: 33637203.

E4 (ii): Kidney Disease: Improving Global Outcomes (KDIGO) Glomerular Diseases Work Group. KDIGO 2021 Clinical Practice Guideline for the Management of Glomerular Diseases. Kidney Int. 2021 Oct;100(4S):S1-S276. doi: 10.1016/j.kint.2021.05.021. PMID: 34556256.

**Section F: Gastrointestinal System criteria.**

**F1. Proton Pump Inhibitor with severe gastro-oesophageal reflux disease or peptic oesophageal stricture requiring dilatation.**

F1 (i): Hungin AP, Raghunath A. Managing gastro-oesophageal reflux disease in the older patient. Digestion 2004; 69 Suppl 1: 17-24. Review. PubMed PMID: 15001831.

F1 (ii): Pilotto A, Franceschi M, Paris F. Recent advances in the treatment of GERD in the elderly: focus on proton pump inhibitors. Int J Clin Pract 2005; 59(10): 1204-9. Review. PubMed PMID: 16178989.

F1 (iii): Metz DC. Managing gastroesophageal reflux disease for the lifetime of the patient: evaluating the long-term options. Am J Med 2004; 117, Suppl 5A :49S-55S. PubMed PMID: 15478853.

**F2. Proton pump inhibitor with initiation of low-dose aspirin and previous history of peptic ulcer or reflux oesophagitis.**

F2 (i): Mo C, Sun G, Lu ML, Zhang L, Wang YZ, Sun X, Yang YS. Proton pump inhibitors in prevention of low-dose aspirin-associated upper gastrointestinal injuries. World J Gastroenterol. 2015 May 7;21(17):5382-92. doi: 10.3748/wjg.v21.i17.5382. PMID: 25954113.

F2 (ii): Szabó IL, Mátics R, Hegyi P, Garami A, Illés A, Sarlós P, Bajor J, Szűcs A, Mosztbacher D, Márta K, Szemes K, Csekő K, Kővári B, Rumbus Z, Vincze Á. PPIs Prevent Aspirin-Induced Gastrointestinal Bleeding Better than H2RAs. A Systematic Review and Meta-analysis. J Gastrointestin Liver Dis. 2017 Dec;26(4):395-402. doi: 10.15403/jgld.2014.1121.264.hra. PMID: 29253055.

F3 (iii): Valkhoff VE, Sturkenboom MC, Kuipers EJ. Risk factors for gastrointestinal bleeding associated with low-dose aspirin. Best Pract Res Clin Gastroenterol. 2012 Apr;26(2):125-40. doi: 10.1016/j.bpg.2012.01.011. PMID: 22542151.

**F3. Proton pump inhibitor with short-term (< 2 weeks) or longer-term (> 2 weeks) NSAID.**

F3 (i): Medlock S, Eslami S, Askari M, Taherzadeh Z, Opondo D, de Rooij SE, Abu-Hanna A. Co-prescription of gastroprotective agents and their efficacy in elderly patients taking nonsteroidal anti-inflammatory drugs: a systematic review of observational studies. Clin Gastroenterol Hepatol. 2013 Oct;11(10):1259-1269.e10. doi: 10.1016/j.cgh.2013.05.034. Epub 2013 Jun 21. PMID: 23792548.

F3 (ii): Rostom A, Dube C, Wells G, Tugwell P, Welch V, Jolicoeur E, McGowan J. Prevention of NSAID-induced gastroduodenal ulcers. Cochrane Database Syst Rev. 2002;(4):CD002296. doi: 10.1002/14651858.CD002296. PMID: 12519573.

**F4. Fibre supplements (e.g., bran, ispaghula, methylcellulose, sterculia) for diverticulosis with a history of constipation.**

F4 (i): Aldoori WH, Giovannucci EL, Rimm EB, Wing AL, Trichopoulos DV, Willett WC. A prospective study of diet and the risk of symptomatic diverticular disease in men. Am J Clin Nutr 1994; 60(5): 757-64. PubMed PMID: 7942584.

F4 (ii): Ünlü C, Daniels L, Vrouenraets BC, Boermeester MA. A systematic review of high-fibre dietary therapy in diverticular disease. Int J Colorectal Dis 2012; 27(4): 419-27. Review. PubMed PMID: 21922199.

F4 (iii): Rocco A, Compare D, Caruso F, Nardone G. Treatment options for uncomplicated diverticular disease of the colon. J Clin Gastroenterol 2009; 43(9): 803-8. Review. PubMed PMID: 19652620.

**F5. Osmotic laxative (e.g., lactulose, macrogol, sorbitol) for chronic persistent idiopathic or secondary benign constipation.**

F5 (i): Klaschik E, Nauck F, Ostgathe C. Constipation--modern laxative therapy. Support Care Cancer. 2003 Nov;11(11):679-85. doi: 10.1007/s00520-003-0525-x. Epub 2003 Sep 20. PMID: 14505158.

F5 (ii): Emmanuel A, Mattace-Raso F, Neri MC, Petersen KU, Rey E, Rogers J. Constipation in older people: A consensus statement. Int J Clin Pract. 2017 Jan;71(1). doi: 10.1111/ijcp.12920. Epub 2016 Dec 9. PMID: 27933718.

F5 (iii): Mounsey A, Raleigh M, Wilson A. Management of Constipation in Older Adults. Am Fam Physician. 2015 Sep 15;92(6):500-4. PMID: 26371734.

**F6. Probiotics used with antibiotics in patients who are not immunocompromised or severely debilitated for the prevention of Clostridioides difficile-associated diarrhoea.**

F6 (i): Ma Y, Yang JY, Peng X, Xiao KY, Xu Q, Wang C. Which probiotic has the best effect on preventing Clostridium difficile-associated diarrhea? A systematic review and network meta-analysis. J Dig Dis. 2020 Feb;21(2):69-80. doi: 10.1111/1751-2980.12839. PMID: 31875427.

F6 (ii): Goldenberg JZ, Yap C, Lytvyn L, Lo CK, Beardsley J, Mertz D, Johnston BC. Probiotics for the prevention of Clostridium difficile-associated diarrhea in adults and children. Cochrane Database Syst Rev. 2017 Dec 19;12(12):CD006095. doi: 10.1002/14651858.CD006095.pub4. PMID: 29257353.

F6 (iii): Pattani R, Palda VA, Hwang SW, Shah PS. Probiotics for the prevention of antibiotic-associated diarrhea and Clostridium difficile infection among hospitalized patients: systematic review and meta-analysis. Open Med. 2013 May 28;7(2):e56-67. PMID: 24348885; PMCID: PMC3863752.

**F7. Helicobacter pylori eradication therapy in HP-associated active peptic ulcer disease.**

F7 (i): Rokkas T, Rokka A, Portincasa P. A systematic review and meta-analysis of the role of Helicobacter pylori eradication in preventing gastric cancer. Ann Gastroenterol. 2017;30(4):414-423. doi: 10.20524/aog.2017.0144. Epub 2017 Apr 7. PMID: 28655977.

F7 (ii): Wong CS, Chia CF, Lee HC, Wei PL, Ma HP, Tsai SH, Wu CH, Tam KW. Eradication of Helicobacter pylori for prevention of ulcer recurrence after simple closure of perforated peptic ulcer: a meta-analysis of randomized controlled trials. J Surg Res. 2013 Jun 15;182(2):219-26. doi: 10.1016/j.jss.2012.10.046. Epub 2012 Nov 9. PMID: 23158404.

F7 (iii): Kuipers EJ. Helicobacter pylori and the risk and management of associated diseases: gastritis, ulcer disease, atrophic gastritis and gastric cancer. Aliment Pharmacol Ther. 1997 Apr;11 Suppl 1:71-88. doi: 10.1046/j.1365-2036.11.s1.5.x. PMID: 9146793.

F7 (iv): Dooley CP, Cohen H, Fitzgibbons PL, Bauer M, Appleman MD, Perez-Perez GI, Blaser MJ. Prevalence of Helicobacter pylori infection and histologic gastritis in asymptomatic persons. N Engl J Med. 1989 Dec 7;321(23):1562-6. doi: 10.1056/NEJM198912073212302. PMID: 2586553.

**Section G: Respiratory System criteria.**

**G1. Long-acting muscarinic antagonist (LAMA e.g., tiotropium, aclidinium, umeclidinium, glycopyrronium) or long-acting beta 2 agonist (LABA e.g., bambuterol, formoterol, indacaterol, olodaterol, salmeterol) for symptomatic COPD of GOLD 1 or 2 severity and chronic asthma.**

G1 (i): Singh D, Agusti A, Anzueto A, Barnes PJ, Bourbeau J, Celli BR, Criner GJ, Frith P, Halpin DMG, Han M, López Varela MV, Martinez F, Montes de Oca M, Papi A, Pavord ID, Roche N, Sin DD, Stockley R, Vestbo J, Wedzicha JA, Vogelmeier C. Global Strategy for the Diagnosis, Management, and Prevention of Chronic Obstructive Lung Disease: the GOLD science committee report 2019. Eur Respir J. 2019 May 18;53(5):1900164. doi: 10.1183/13993003.00164-2019. PMID: 30846476.

G1 (ii): Kew KM, Dias S, Cates CJ. Long-acting inhaled therapy (beta-agonists, anticholinergics and steroids) for COPD: a network meta-analysis. Cochrane Database Syst Rev. 2014 Mar 26;(3):CD010844. doi: 10.1002/14651858.CD010844.pub2. PMID: 24671923.

G1 (iii): Reddel HK, Bacharier LB, Bateman ED, Brightling CE, Brusselle GG, Buhl R, Cruz AA, Duijts L, Drazen JM, FitzGerald JM, Fleming LJ, Inoue H, Ko FW, Krishnan JA, Levy ML, Lin J, Mortimer K, Pitrez PM, Sheikh A, Yorgancioglu AA, Boulet LP. Global Initiative for Asthma Strategy 2021: executive summary and rationale for key changes. Eur Respir J. 2021 Dec 31;59(1):2102730. doi: 10.1183/13993003.02730-2021. PMID: 34667060; PMCID: PMC8719459.

**G2. Regular i.e. daily inhaled corticosteroid (e.g., beclomethasone, budesonide, ciclesonide, fluticasone, mometasone) for moderate-severe asthma or COPD of GOLD 3 or 4 severity, where FEV1 <50% of predicted value and repeated exacerbations requiring treatment with oral corticosteroids.**

G2 (i): Spencer S, Evans DJ, Karner C, Cates CJ.Inhaled corticosteroids versus long-acting beta 2-agonists for chronic obstructive pulmonary disease. Cochrane Database Syst Rev. 2011 Oct 5;(10):CD007033. doi: 10.1002/14651858.CD007033.pub2. Review. Update in: Cochrane Database Syst Rev 2011; (12):CD007033. PubMed PMID: 21975759.

G2 (ii): Gaebel K, McIvor RA, Xie F, Blackhouse G, Robertson D, Assasi N, Hernandez P,

Goeree R. Triple therapy for the management of COPD: a review. COPD 2011; 8(3): 206-43. Review. PubMed PMID: 21513437.

G2 (iii): Singh D, Agusti A, Anzueto A, Barnes PJ, Bourbeau J, Celli BR, Criner GJ, Frith P, Halpin DMG, Han M, López Varela MV, Martinez F, Montes de Oca M, Papi A, Pavord ID, Roche N, Sin DD, Stockley R, Vestbo J, Wedzicha JA, Vogelmeier C. Global Strategy for the Diagnosis, Management, and Prevention of Chronic Obstructive Lung Disease: the GOLD science committee report 2019. Eur Respir J. 2019 May 18;53(5):1900164. doi: 10.1183/13993003.00164-2019. PMID: 30846476.

**G3. Home continuous oxygen with documented chronic hypoxaemia (i.e., pO2 < 8.0 kPa or 60 mmHg or SaO2 < 89%).**

G3 (i): Cranston JM, Crockett AJ, Moss JR, Alpers JH. Domiciliary oxygen for chronic obstructive pulmonary disease. Cochrane Database Syst Rev 2005 Oct 19; (4):CD001744. Review. PubMed PMID: 16235285.

G3 (ii): Weitzenblum E, Chaouat A, Kessler R. [Long-term oxygen therapy for chronic respiratory failure. Rationale, indications, modalities]. Rev Pneumol Clin 2002; 58(4 Pt 1): 195-212. Review. French. PubMed PMID: 12407284.

**Section H: Musculoskeletal System criteria.**

**H1. Disease-modifying anti-rheumatic drug (DMARD) with chronic, active and disabling rheumatoid arthritis.**

H1(i): Saag KG, Teng GG, Patkar NM, Anuntiyo J, Finney C, Curtis JR, Paulus HE,Mudano A, Pisu M, Elkins-Melton M, Outman R, Allison JJ, Suarez Almazor M,Bridges SL Jr, Chatham WW, Hochberg M, MacLean C, Mikuls T, Moreland LW, O'DellJ, Turkiewicz AM, Furst DE; American College of Rheumatology. American College of Rheumatology 2008 recommendations for the use of nonbiologic and biologic disease-modifying antirheumatic drugs in rheumatoid arthritis. Arthritis Rheum 2008; 59(6): 762-84. PubMed PMID: 18512708.

H1 (ii): Köller MD, Aletaha D, Funovits J, Pangan A, Baker D, Smolen JS. Response of elderly patients with rheumatoid arthritis to methotrexate or TNF inhibitors compared with younger patients. Rheumatology (Oxford) 2009; 48(12): 1575-80.PubMed PMID: 19812228.

H1 (iii): Fleischmann R, Baumgartner SW, Weisman MH, Liu T, White B, Peloso P. Long term safety of etanercept in elderly subjects with rheumatic diseases. Ann Rheum Dis 2006; 65(3): 379-84. PubMed PMID: 16150792.

**H2. Bisphosphonates and vitamin D and calcium in patients taking long-term systemic corticosteroid therapy for prevention of steroid-induced osteoporosis.**

H2 (i): Homik J, Suarez-Almazor ME, Shea B, Cranney A, Wells G, Tugwell P. Calcium and vitamin D for corticosteroid-induced osteoporosis. Cochrane Database Syst Rev 2000; (2): CD000952. Review. PubMed PMID: 10796394.

H2 (ii): Homik J, Cranney A, Shea B, Tugwell P, Wells G, Adachi R, Suarez-Almazor M. Bisphosphonates for steroid induced osteoporosis. Cochrane Database Syst Rev 2000; (2):CD001347. Review. PubMed PMID: 10796432.

H2 (iii): Iwamoto J, Takeda T, Sato Y. Effects of antifracture drugs in postmenopausal, male and glucocorticoid-induced osteoporosis--usefulness of alendronate and risedronate. Expert Opin Pharmacother 2007; 8(16): 2743-56. Review. PubMedPMID: 17956196.

**H3. Vitamin D in patients with known osteoporosis and/or previous fragility fracture(s) and/or Bone Mineral Density T-scores below -2.5 in one or multiple sites.**

H3 (i): Avenell A, Gillespie WJ, Gillespie LD, O'Connell D. Vitamin D and vitamin D analogues for preventing fractures associated with involutional and post-menopausal osteoporosis. Cochrane Database Syst Rev 2009 Apr15; (2):CD000227. doi: 10.1002/14651858.CD000227.pub3. Review. PubMed PMID:19370554.

H3 (ii): Bischoff-Ferrari HA, Willett WC, Orav EJ, Lips P, Meunier PJ, Lyons RA,Flicker L, Wark J, Jackson RD, Cauley JA, Meyer HE, Pfeifer M, Sanders KM,Stähelin HB, Theiler R, Dawson-Hughes B. A pooled analysis of vitamin D dose requirements for fracture prevention. N Engl J Med 2012; 367(1):40-9. Erratum in: N Engl J Med. 2012 Aug 2;367(5):481. Oray, Endel J [corrected to Orav, Endel J]. PubMed PMID: 22762317.

H3 (iii): Montero-Odasso M, van der Velde N, Martin FC, Petrovic M, Tan MP, Ryg J, Aguilar-Navarro S, Alexander NB, Becker C, Blain H, Bourke R, Cameron ID, Camicioli R, Clemson L, Close J, Delbaere K, Duan L, Duque G, Dyer SM, Freiberger E, Ganz DA, Gómez F, Hausdorff JM, Hogan DB, Hunter SMW, Jauregui JR, Kamkar N, Kenny RA, Lamb SE, Latham NK, Lipsitz LA, Liu-Ambrose T, Logan P, Lord SR, Mallet L, Marsh D, Milisen K, Moctezuma-Gallegos R, Morris ME, Nieuwboer A, Perracini MR, Pieruccini-Faria F, Pighills A, Said C, Sejdic E, Sherrington C, Skelton DA, Dsouza S, Speechley M, Stark S, Todd C, Troen BR, van der Cammen T, Verghese J, Vlaeyen E, Watt JA, Masud T; Task Force on Global Guidelines for Falls in Older Adults. World guidelines for falls prevention and management for older adults: a global initiative. Age Ageing. 2022 Sep 2;51(9):afac205. doi: 10.1093/ageing/afac205. PMID: 36178003; PMCID: PMC9523684.

**H4. Bone anti-resorptive or anabolic therapy (e.g., bisphosphonate, teriparatide, denosumab) in patients with documented osteoporosis (Bone Mineral Density T-scores below -2.5 in one or multiple sites) and/or previous history of fragility fracture(s) – where no pharmacological or clinical status contraindication exists such as poor one-year life expectancy.**

H4 (i): Wells GA, Cranney A, Peterson J, Boucher M, Shea B, Robinson V, Coyle D, Tugwell P. Alendronate for the primary and secondary prevention of osteoporotic fractures in postmenopausal women. Cochrane Database Syst Rev 2008 Jan23; (1): CD001155. doi: 10.1002/14651858.CD001155.pub2. Review. PubMed PMID: 18253985.

H4 (ii): O'Donnell S, Cranney A, Wells GA, Adachi JD, Reginster JY. Strontium ranelate for preventing and treating postmenopausal osteoporosis. Cochrane Database Syst Rev 2006 Oct 18;(4):CD005326. Review. PubMed PMID: 17054253.

H4 (iii): Nakamura T, Tsujimoto M, Hamaya E, Sowa H, Chen P. Consistency of fracture risk reduction in Japanese and Caucasian osteoporosis patients treated with teriparatide: a meta-analysis. J Bone Miner Metab 2012; 30(3): 321-5. PubMed PMID: 21938382.

H4 (iv): von Keyserlingk C, Hopkins R, Anastasilakis A, Toulis K, Goeree R, Tarride JE,Xie F. Clinical efficacy and safety of denosumab in postmenopausal women with low bone mineral density and osteoporosis: a meta-analysis. Semin Arthritis Rheum 2011; 41(2):178-86. PubMed PMID: 21616520.

**H5. Vitamin D supplement in older people with confirmed 25-hydroxycolecalciferol deficiency (< 20 micrograms/L, < 50 nmol/L) who are housebound or experiencing falls or with osteopenia (Bone Mineral Density T-score is less than -1.0 but above -2.5 in one or multiple sites).**

H5 (i): Cameron ID, Gillespie LD, Robertson MC, Murray GR, Hill KD, Cumming RG, Kerse N. Interventions for preventing falls in older people in care facilities and hospitals. Cochrane Database Syst Rev 2012 Dec 12;12:CD005465. doi:10.1002/14651858.CD005465.pub3. Review. PubMed PMID: 23235623.

H5 (ii): Michael YL, Whitlock EP, Lin JS, Fu R, O'Connor EA, Gold R; US Preventive Services Task Force. Primary care-relevant interventions to prevent falling in older adults: a systematic evidence review for the U.S. Preventive Services Task Force. Ann Intern Med 2010; 153(12): 815-25. Review. PubMed PMID: 21173416.

H5 (iii): Kalyani RR, Stein B, Valiyil R, Manno R, Maynard JW, Crews DC. Vitamin D treatment for the prevention of falls in older adults: systematic review and meta-analysis. J Am Geriatr Soc 2010; 58(7): 1299-310. Review. PubMed PMID:20579169.

H5 (iv): Chevalley T, Brandi ML, Cashman KD, Cavalier E, Harvey NC, Maggi S, Cooper C, Al-Daghri N, Bock O, Bruyère O, Rosa MM, Cortet B, Cruz-Jentoft AJ, Cherubini A, Dawson-Hughes B, Fielding R, Fuggle N, Halbout P, Kanis JA, Kaufman JM, Lamy O, Laslop A, Yerro MCP, Radermecker R, Thiyagarajan JA, Thomas T, Veronese N, de Wit M, Reginster JY, Rizzoli R. Role of vitamin D supplementation in the management of musculoskeletal diseases: update from an European Society of Clinical and Economical Aspects of Osteoporosis, Osteoarthritis and Musculoskeletal Diseases (ESCEO) working group. Aging Clin Exp Res. 2022 Nov;34(11):2603-2623.

**H6. Anti-resorptive treatment after discontinuation of at least two doses of denosumab (rebound increased bone turnover markers, BMD loss, and increased risk of vertebral fracture following denosumab discontinuation).**

H6 (i): Bone HG, Bolognese MA, Yuen CK, Kendler DL, Miller PD, Yang YC, Grazette L, San Martin J, Gallagher JC. Effects of denosumab treatment and discontinuation on bone mineral density and bone turnover markers in postmenopausal women with low bone mass. J Clin Endocrinol Metab. 2011 Apr;96(4):972-80. doi: 10.1210/jc.2010-1502. Epub 2011 Feb 2. PMID: 21289258.

H6 (ii): Tsourdi E, Zillikens MC, Meier C, Body JJ, Gonzalez Rodriguez E, Anastasilakis AD, Abrahamsen B, McCloskey E, Hofbauer LC, Guañabens N, Obermayer-Pietsch B, Ralston SH, Eastell R, Pepe J, Palermo A, Langdahl B. Fracture risk and management of discontinuation of denosumab therapy: a systematic review and position statement by ECTS. J Clin Endocrinol Metab. 2020 Oct 26:dgaa756. doi: 10.1210/clinem/dgaa756. Epub ahead of print. PMID: 33103722.

**H7. Anti-resorptive treatment after discontinuation of teriparatide/abaloparatide treatment for osteoporosis.**

H7 (i): Ebina K, Hashimoto J, Kashii M, Hirao M, Kaneshiro S, Noguchi T, Tsukamoto Y, Yoshikawa H. The effects of switching daily teriparatide to oral bisphosphonates or denosumab in patients with primary osteoporosis. J Bone Miner Metab. 2017 Jan;35(1):91-98. doi: 10.1007/s00774-015-0731-x. Epub 2016 Jan 13. PMID: 26762133.

H7 (ii): Cosman F, Miller PD, Williams GC, Hattersley G, Hu MY, Valter I, Fitzpatrick LA, Riis BJ, Christiansen C, Bilezikian JP, Black D. Eighteen Months of Treatment With Subcutaneous Abaloparatide Followed by 6 Months of Treatment With Alendronate in Postmenopausal Women With Osteoporosis: Results of the ACTIVExtend Trial. Mayo Clin Proc. 2017 Feb;92(2):200-210. doi: 10.1016/j.mayocp.2016.10.009. PMID: 28160873

H7 (iii): Kurland ES, Heller SL, Diamond B, McMahon DJ, Cosman F, Bilezikian JP. The importance of bisphosphonate therapy in maintaining bone mass in men after therapy with teriparatide [human parathyroid hormone(1-34)]. Osteoporos Int. 2004 Dec;15(12):992-7. doi: 10.1007/s00198-004-1636-z. Epub 2004 Jun 3. PMID: 15175844.

**H8. Xanthine-oxidase inhibitors (e.g. allopurinol, febuxostat) with a history of recurrent episodes of gout.**

H8 (i): Fravel MA, Ernst ME. Management of gout in the older adult. Am J Geriatr Pharmacother 2011; 9(5): 271-85. Review. PubMed PMID: 21849262.

H8 (ii): Zhang W, Doherty M, Bardin T, Pascual E, Barskova V, Conaghan P, Gerster J,Jacobs J, Leeb B, Lioté F, McCarthy G, Netter P, Nuki G, Perez-Ruiz F, Pignone A,Pimentão J, Punzi L, Roddy E, Uhlig T, Zimmermann-Gòrska I; EULAR Standing Committee for International Clinical Studies Including Therapeutics. EULAR evidence based recommendations for gout. Part II: Management. Report of a task force of the EULAR Standing Committee for International Clinical Studies Including Therapeutics (ESCISIT). Ann Rheum Dis. 2006; 65(10): 1312-24. Review. PubMed PMID: 16707532.

H8 (iii): Tayar JH, Lopez-Olivo MA, Suarez-Almazor ME. Febuxostat for treating chronic gout. Cochrane Database Syst Rev. 2012 Nov 14;11:CD008653. doi:10.1002/14651858.CD008653.pub2. Review. PubMed PMID: 23152264.

**H9. Folic acid supplement in patients taking methotrexate.**

H9 (i): Visser K, Katchamart W, Loza E, Martinez-Lopez JA, Salliot C, Trudeau J,Bombardier C, Carmona L, van der Heijde D, Bijlsma JW, Boumpas DT, Canhao H,Edwards CJ, Hamuryudan V, Kvien TK, Leeb BF, Martín-Mola EM, Mielants H,Müller-Ladner U, Murphy G, Østergaard M, Pereira IA, Ramos-Remus C, Valentini G, Zochling J, Dougados M. Multinational evidence-based recommendations for the use of methotrexate in rheumatic disorders with a focus on rheumatoid arthritis: integrating systematic literature research and expert opinion of a broad international panel of rheumatologists in the 3E Initiative. Ann Rheum Dis 2009; 68(7): 1086-93. PubMed PMID: 19033291.

H9 (ii): Ortiz Z, Shea B, Suarez Almazor M, Moher D, Wells G, Tugwell P. Folic acid and folinic acid for reducing side effects in patients receiving methotrexate for rheumatoid arthritis. Cochrane Database Syst Rev 2000; (2):CD000951. Review. PubMed PMID: 10796393.

**Section I: Urogenital System.**

**I1. Selective alpha-1 receptor blocker (e.g., tamsulosin, silodosin) for lower urinary tract symptoms related to benign prostatic hyperplasia where prostatectomy is not considered necessary or appropriate or safe.**

I1 (i): Lowe FC. Role of the newer alpha-adrenergic-receptor antagonists in the treatment of benign prostatic hyperplasia-related lower urinary tract symptoms. Clin Ther 2004; 26(11): 1701-13. Review. PubMed PMID: 15639685.

I1 (ii): Schwinn DA, Roehrborn CG. Alpha1-adrenoceptor subtypes and lower urinary tract symptoms. Int J Urol 2008; 15(3):193-9. Review. PubMed PMID: 18304211.

I1(iii): Dunn CJ, Matheson A, Faulds DM. Tamsulosin: a review of its pharmacology and therapeutic efficacy in the management of lower urinary tract symptoms. Drugs Aging 2002; 19(2):135-61. Review. PubMed PMID: 11950378.

**I2. 5-alpha reductase inhibitor (e.g., finasteride, dutasteride) for lower urinary tract symptoms related to benign prostatic hyperplasia where prostatectomy is not considered necessary or appropriate or safe.**

I2 (i): Tacklind J, Fink HA, Macdonald R, Rutks I, Wilt TJ. Finasteride for benign prostatic hyperplasia. Cochrane Database Syst Rev. 2010 Oct 6;(10): CD006015. doi: 10.1002/14651858.CD006015.pub3. Review. PubMed PMID: 20927745.

I2 (ii): O'Leary MP, Roehrborn CG, Black L. Dutasteride significantly improves quality of life measures in patients with enlarged prostate. Prostate Cancer Prostatic Dis 2008; 11(2):129-33. PubMed PMID: 17592479.

I2 (iii): Roehrborn CG. BPH progression: concept and key learning from MTOPS, ALTESS, COMBAT, and ALF-ONE. BJU Int 2008; 101 Suppl 3: 17-21. Review. PubMed PMID: 18307681.

**I3. Topical vaginal oestrogen or vaginal oestrogen pessary for symptomatic atrophic vaginitis.**

I3 (i): Lynch C. Vaginal estrogen therapy for the treatment of atrophic vaginitis. J Womens Health (Larchmt) 2009; 18(10): 1595-606. Review. PubMed PMID: 19788364.

I3 (ii): Bachmann G, Bouchard C, Hoppe D, Ranganath R, Altomare C, Vieweg A, Graepel J, Helzner E. Efficacy and safety of low-dose regimens of conjugated estrogens cream administered vaginally. Menopause 2009; 16(4): 719-27.PubMed PMID: 19436223.

I3 (iii): Mainini G, Scaffa C, Rotondi M, Messalli EM, Quirino L, Ragucci A. Local estrogen replacement therapy in postmenopausal atrophic vaginitis: efficacy and safety of low dose 17beta-estradiol vaginal tablets. Clin Exp Obstet Gynecol 2005; 32(2): 111-3. PubMed PMID: 16108394.

**I4. Topical vaginal oestrogen or vaginal oestrogen pessary in women for recurrent urinary tract infections.**

I4 (i): Dueñas-Garcia OF, Sullivan G, Hall CD, Flynn MK, OʼDell K. Pharmacological Agents to Decrease New Episodes of Recurrent Lower Urinary Tract Infections in Postmenopausal Women. A Systematic Review. Female Pelvic Med Reconstr Surg. 2016 Mar-Apr;22(2):63-9. doi: 10.1097/SPV.0000000000000244. PMID: 26825411.

I4 (ii): Perrotta C, Aznar M, Mejia R, Albert X, Ng CW. Oestrogens for preventing recurrent urinary tract infection in postmenopausal women. Cochrane Database Syst Rev. 2008 Apr 16;(2):CD005131. doi: 10.1002/14651858.CD005131.pub2. PMID: 18425910.

I4 (iii): Raz R, Stamm WE. A controlled trial of intravaginal estriol in postmenopausal women with recurrent urinary tract infections. N Engl J Med. 1993 Sep 9;329(11):753-6. doi: 10.1056/NEJM199309093291102. PMID: 8350884.

**I5. Phosphodiesterase type-5 inhibitors (e.g., avanafil, sildenafil, tadalafil, vardenafil) for persistent erectile dysfunction that causes distress.**

I5 (i): Fink HA, Mac Donald R, Rutks IR, Nelson DB, Wilt TJ. Sildenafil for male erectile dysfunction: a systematic review and meta-analysis. Arch Intern Med. 2002 Jun 24;162(12):1349-60. doi: 10.1001/archinte.162.12.1349. PMID: 12076233.

I5 (ii): Corbin JD, Francis SH. Pharmacology of phosphodiesterase-5 inhibitors. Int J Clin Pract. 2002 Jul-Aug;56(6):453-9. PMID: 12166544.

I5 (iii): Huang SA, Lie JD. Phosphodiesterase-5 (PDE5) Inhibitors In the Management of Erectile Dysfunction. P T. 2013 Jul;38(7):407-19. PMID: 24049429; PMCID: PMC3776492.

**Section J: Endocrine System criteria.**

**J1. ACE inhibitor or Angiotensin Receptor Blocker (if intolerant of ACE inhibitor) in diabetes with evidence of renal disease i.e., dipstick proteinuria or microalbuminuria (>30 mg/24 hours) unless evidence of severe CKD (eGFR < 30 ml/min/m^2^).**

J1 (i): Lv J, Perkovic V, Foote CV, Craig ME, Craig JC, Strippoli GF. Antihypertensive agents for preventing diabetic kidney disease. Cochrane Database Syst Rev 2012 Dec 12;12:CD004136. doi: 10.1002/14651858.CD004136.pub3. Review. PubMed PMID:23235603.

J1 (ii): Strippoli GF, Bonifati C, Craig M, Navaneethan SD, Craig JC. Angiotensin converting enzyme inhibitors and angiotensin II receptor antagonists for preventing the progression of diabetic kidney disease. Cochrane Database Syst Rev 2006 Oct 18;(4):CD006257. Review. PubMed PMID: 17054288.

J1 (iii): Blacklock CL, Hirst JA, Taylor KS, Stevens RJ, Roberts NW, Farmer AJ. Evidence for a dose effect of renin-angiotensin system inhibition on progression of microalbuminuria in Type 2 diabetes: a meta-analysis. Diabet Med 2011; 28(10): 1182-7. PubMed PMID: 21627686.

**Section K: Analgesics criteria.**

**K1. High-potency opioids in moderate-severe non-arthritis pain, where paracetamol, NSAIDs or low-potency opioids are not appropriate to the pain severity or have been ineffective.**

K1 (i): Papaleontiou M, Henderson CR Jr, Turner BJ, Moore AA, Olkhovskaya Y, Amanfo L,Reid MC. Outcomes associated with opioid use in the treatment of chronic non-cancer pain in older adults: a systematic review and meta-analysis. J Am Geriatr Soc 2010; 58(7): 1353-69. Review. PubMed PMID: 20533971.

K1 (ii): van Ojik AL, Jansen PA, Brouwers JR, van Roon EN. Treatment of chronic pain in older people: evidence-based choice of strong-acting opioids. Drugs Aging 2012; 29(8): 615-25. Review. PubMed PMID: 22765848.

**K2. Laxatives in patients receiving opioids regularly i.e., other than PRN use.**

K2 (i): Cook SF, Lanza L, Zhou X, Sweeney CT, Goss D, Hollis K, Mangel AW, Fehnel SE. Gastrointestinal side effects in chronic opioid users: results from a population-based survey. Aliment Pharmacol Ther 2008; 27(12): 1224-32. PubMed PMID: 18363893.

K2 (ii): Chodosh J, Ferrell BA, Shekelle PG, Wenger NS. Quality indicators for pain management in vulnerable elders. Ann Intern Med 2001; 135(8 Pt 2): 731-5. PubMed PMID: 11601956.

**K3. Topical 5% lidocaine (lignocaine) patch for localized neuropathic pain, e.g. post-herpetic neuralgia.**

K3 (i): Finnerup NB, Attal N, Haroutounian S, McNicol E, Baron R, Dworkin RH, Gilron I, Haanpää M, Hansson P, Jensen TS, Kamerman PR, Lund K, Moore A, Raja SN, Rice ASC, Rowbotham M, Sena E, Wallace M. Pharmacotherapy for neuropathic pain in adults: a systematic review and meta-analysis. Lancet Neurol. 2015;14(2):162–73.

K3 (ii): Dworkin RH, O’Connor AB, Backonja M, Farrar JT, Finnerup NB, Jensen TS, Kalso EA, Loeser JD, Miaskowski C, Nurmikko TJ, Portenoy RK, Rice ASC, Stacey BR, Treede RD, Turk DC, Wallace MS. Pharmacologic management of neuropathic pain: Evidence-based recommendations. Pain. 2007;132(3):237–51.

**Section L: Vaccines criteria.**

**L1: Seasonal influenza vaccine annually.**

L1 (i): Lam S, Jodlowski TZ. Vaccines for older adults. Consult Pharm 2009; 24(5): 380-91. Review. PubMed PMID: 19555147.

L1 (ii): Nichol KL, Nordin JD, Nelson DB, Mullooly JP, Hak E. Effectiveness of influenza vaccine in the community-dwelling elderly. N Engl J Med 2007; 357(14): 1373-81. PubMed PMID: 17914038.

L1 (iii): Michel JP, Chidiac C, Grubeck-Loebenstein B, Johnson RW, Lambert PH, Maggi S, Moulias R, Nicholson K, Werner H. Advocating vaccination of adults aged 60 years and older in Western Europe: statement by the Joint Vaccine Working Group of the European Union Geriatric Medicine Society and the International Association of Gerontology and Geriatrics-European Region. Rejuvenation Res 2009; 12(2): 127-35. PubMed PMID: 19415978.

L1 (iv): Leibovici Weissman Y, Cooper L, Sternbach N, Ashkenazi-Hoffnung L, Yahav D. Clinical efficacy and safety of high dose trivalent influenza vaccine in adults and immunosuppressed populations - A systematic review and meta-analysis. J Infect. 2021 Oct;83(4):444-451. doi: 10.1016/j.jinf.2021.08.028. Epub 2021 Aug 20. PMID: 34425161.

**L2: Pneumococcal vaccine at least once according to national guidelines.**

L2 (i): Fedson DS, Liss C. Precise answers to the wrong question: prospective clinical trials and the meta-analyses of pneumococcal vaccine in elderly and high-risk adults. Vaccine 2004; 22(8): 927-46. PubMed PMID: 15161070.

L2 (ii): Vila-Córcoles A, Ochoa-Gondar O, Hospital I, Ansa X, Vilanova A, Rodríguez T, Llor C; EVAN Study Group. Protective effects of the 23-valent pneumococcal polysaccharide vaccine in the elderly population: the EVAN-65 study. Clin Infect Dis 2006; 43(7): 860-8. PubMed PMID: 16941367.

L2 (iii): Vila-Corcoles A, Ochoa-Gondar O. Preventing pneumococcal disease in the elderly: recent advances in vaccines and implications for clinical practice. Drugs Aging 2013; 30(5): 263-76. Review. PubMed PMID: 23420119.

**L3. Varicella-zoster vaccine according to national guidelines.**

L3 (i): Gagliardi AM, Andriolo BN, Torloni MR, Soares BG, de Oliveira Gomes J, Andriolo RB, Canteiro Cruz E. Vaccines for preventing herpes zoster in older adults. Cochrane Database Syst Rev. 2019 Nov 7;2019(11):CD008858. doi: 10.1002/14651858.CD008858.pub4. PMID: 31696946.

L3 (ii): Tseng HF, Smith N, Harpaz R, Bialek SR, Sy LS, Jacobsen SJ. Herpes zoster vaccine in older adults and the risk of subsequent herpes zoster disease. JAMA. 2011 Jan 12;305(2):160-6. doi: 10.1001/jama.2010.1983. PMID: 21224457.

**L4. SARS-CoV2 vaccine according to national guidelines.**

L4 (i): Creech CB, Walker SC, Samuels RJ. SARS-CoV-2 Vaccines. JAMA. 2021 Apr 6;325(13):1318-1320. doi: 10.1001/jama.2021.3199. PMID: 33635317.

L4 (ii): Ghazy RM, Ashmawy R, Hamdy NA, Elhadi YAM, Reyad OA, Elmalawany D, Almaghraby A, Shaaban R, Taha SHN. Efficacy and Effectiveness of SARS-CoV-2 Vaccines: A Systematic Review and Meta-Analysis. Vaccines (Basel). 2022 Feb 23;10(3):350. doi: 10.3390/vaccines10030350. PMID: 35334982
